# The adaptive nature of the foam proteome produced by *Mahanarva spectabilis* (Hemiptera: Cercopidae) when infesting forage grasses with different levels of antibiosis-type resistance

**DOI:** 10.1038/s41598-026-36784-9

**Published:** 2026-02-03

**Authors:** Angelo José Rinaldi, Monique Silva Bonjour, Edvaldo Barros, Alexander Machado Auad, Gabriely Teixeira Bhering Faria, Maria Goreti Almeida Oliveira, Humberto Josué de Oliveira Ramos, Jorge Fernando Pereira

**Affiliations:** 1 Laboratório de Enzimologia e Bioquímica de Proteínas e Peptídeos, Departamento de Bioquímica e Biologia Molecular, Universidade Federal de Viçosa, BIOAGRO/INCT-IPP, Viçosa, MG Brazil; 2 Núcleo de Análise de Biomoléculas (NuBioMol), Centro de Ciências Biológicas, Universidade Federal de Viçosa, Viçosa, MG Brazil; 3 Embrapa Gado de Leite, Juiz de Fora, MG Brazil

**Keywords:** Forage plants, Insect pest, Plant defence, Spittlebug, Biochemistry, Ecology, Ecology, Microbiology, Plant sciences

## Abstract

The spittlebug *Mahanarva spectabilis* (Distant, 1909) (Hemiptera: Cercopidae) produces a stable extracellular foam during its nymphal stage, which plays a critical role in survival and host plant interactions. In this study, we present the first comprehensive proteomic characterization of foam secreted by *M. spectabilis* nymphs, using a shotgun LC–MS/MS approach. We analyzed the foam produced by nymphs feeding on four forage cultivars showing different levels of antibiosis-type resistance against *M. spectabilis*, as follows: *Cenchrus purpureus* cv. Pioneiro (moderately resistant) and cv. Roxo de Botucatu (susceptible); *Urochloa brizantha* cv. Marandu (resistant), and *Urochloa decumbens* cv. Basilisk (susceptible). A total of 196 proteins were identified, including a substantial fraction of unannotated proteins with high abundance, suggesting specialized foam-specific functions. Functional annotation revealed enrichment in hydrolases, oxidoreductases, and binding proteins, highlighting potential roles in microbial regulation, stress response, and structural maintenance. Comparative analysis revealed consistent up-regulation of cytoskeletal and metabolic proteins in resistant/moderately resistant hosts, alongside repression of proteins related to carbohydrate and lipid metabolism. Multivariate and GO-based analyses confirmed host genotype-dependent modulation of foam composition. The findings demonstrate that higher levels of resistance to *M. spectabilis* are associated with two key strategies: (1) the suppression of metabolic pathways, likely limiting nutrient availability to the insect, and (2) the activation of defence-related proteins, such as antioxidant enzymes, which enhance the plant’s ability to cope with stress. These findings underscore the dynamic and adaptive nature of the foam proteome, reflecting both environmental and physiological constraints. Our results provide new insights into the molecular basis of foam function and its relevance for insect survival, offering promising avenues for the development of novel strategies targeting foam-mediated defence mechanisms.

**Supplementary Information:**

The online version contains supplementary material available at 10.1038/s41598-026-36784-9.

## Introduction

Spittlebugs (Hemiptera: Cercopidae) are important pests in tropical forage grasses, particularly due to the damage caused by both adult and nymphal stages^1^. Among the different species of spittlebugs, *Mahanarva spectabilis* (Distant, 1909) (Hemiptera: Cercopidae) is one of the most aggressive affecting pastures in South America, causing substantial losses in biomass and forage quality^2,3^. This economic impact underscores the critical need for effective pest management strategies, highlighting the importance of understanding insect-plant interactions in these agricultural systems^4^. A distinctive feature of spittlebug nymphs is the production of a foamy secretion, commonly referred to as spittle or foam, which envelops the insect during its development on the host plant. This foam serves as a physical and biochemical barrier, providing protection against environmental stressors such as dehydration, temperature fluctuations, and natural enemies^5,6^. Beyond physical protection, the foam seems to be a dynamic interface determined by the interaction between the insect and its host plant.

Previous studies have shown that the susceptibility of forage grasses to *Mahanarva spectabilis* nymph infestation varies significantly among genotypes. In particular, elephant grass [*Cenchrus purpureus* (syn. *Pennisetum purpureum*)] cv. Pioneiro (here designated as PIO) and *C. purpureus* cv. Roxo de Botucatu (ROXO) have differences in resistance levels where ROXO is susceptible and PIO shows significantly higher antibiosis-type resistance being considered moderately resistant^7,8^; besides, *Urochloa brizantha* (syn. *Brachiaria brizantha*) cv. Marandu (BRI) is characterized as resistant whereas *U. decumbens* cv. Basilisk (DEC) is classified as susceptible^9,10^. The differences in the antibiosis-type resistance levels are theoretically associated with the biochemical composition of the sap that influences the nymph survival (the greater the resistance level the higher the nymph mortality). Plant resistance mechanisms, such as the production of secondary metabolites or defence proteins, can also directly or indirectly modulate insect secretions^11^.

The biochemical and molecular composition of the foam secreted by nymphs of different species of spittlebugs has been the subject of limited investigation, despite its critical ecological and physiological functions. For instance, Sahayaraj et al.^5^ demonstrated that the foam produced by *Poophilus costalis* was rich in fatty acids, with octadecanoic acid representing over 88% of the lipid fraction. The foam contained free sugars and amino acids, had a role in temperature buffering and also exhibited antimicrobial activities against bacteria, highlighting its role in microbial defence^5^. Auad et al.^12^ identified genotype-dependent variations in *Mahanarva spectabilis* foam proteins via SDS-PAGE, notably revealing a 53–57 kDa protein band consistently associated with susceptible hosts. Tonelli et al.^6^ performed a gas chromatography-mass spectrometry (GC–MS) based metabolomic analysis of the foam produced by *Mahanarva fimbriolata* when infesting sugarcane. Their study identified a diverse set of compounds, including saturated fatty acids such as palmitic and stearic acid, as well as carbohydrates and amino acids. They also found total protein represents 320 ± 50 µg ml^–1^ of the foam. The foam metabolites were proposed to contribute to its thermal stability and modulation, supporting the hypothesis that the secretion acts as a thermoregulatory and protective barrier for nymphs under abiotic stress conditions^6^. Additionally, the spittlebug foam also shows adhesive properties that protects the insect against environmental stresses and predators, as demonstrated in *Aphrophora alni*^13^.

This multifunctionality of the foam produced by spittlebugs underscores the need to understand its molecular composition, particularly in the context of host plant resistance. This reinforces the concept that insect secretions are complex biological matrices with diverse functions beyond simple physical protection, often reflecting adaptations to specific ecological niches and host interactions^14^. Also, the foam biochemical composition differs depending on the species of spittlebug and its host plant. However, although some of these studies evaluated foam produced by different spittlebug species infesting different plant species, no protein identification or functional annotation was performed, leaving the biological roles of the foam proteins unresolved. This knowledge gap is particularly relevant because forage grasses, such as *Urochloa brizantha*, *Urochloa decumbens*, and *Cenchrus purpureus*, exhibit varying levels of antibiosis-type resistance to *M. spectabilis* infestation. Thus, uncovering how the foam proteome varies across different host plants could reveal critical insights into insect-plant interactions and the strategies used by the insect to manipulate or resist plant defence. The proteomic analysis of insect secretions, therefore, offers a powerful tool to dissect the molecular dialogue occurring at the insect-plant interface, providing valuable targets for integrated pest management^15^.

To date, no study has provided a comprehensive proteomic characterization of the foam in *Mahanarva* species, nor has any investigation explored the functional roles of its proteins or their regulation by different host plant genotypes. In this study, we aimed to address this gap through two main objectives: (1) to functionally characterize the protein content of the foam produced by *M. spectabilis* nymphs infesting different forage grasses using liquid chromatography-tandem mass spectrometry (LC–MS/MS) based shotgun proteomics; and (2) to evaluate the effect of host plant genotype on both the qualitative and quantitative profiles of the foam proteins.

## Results

### Proteomic identification and functional classification by gene ontology

**Fig. 1:**
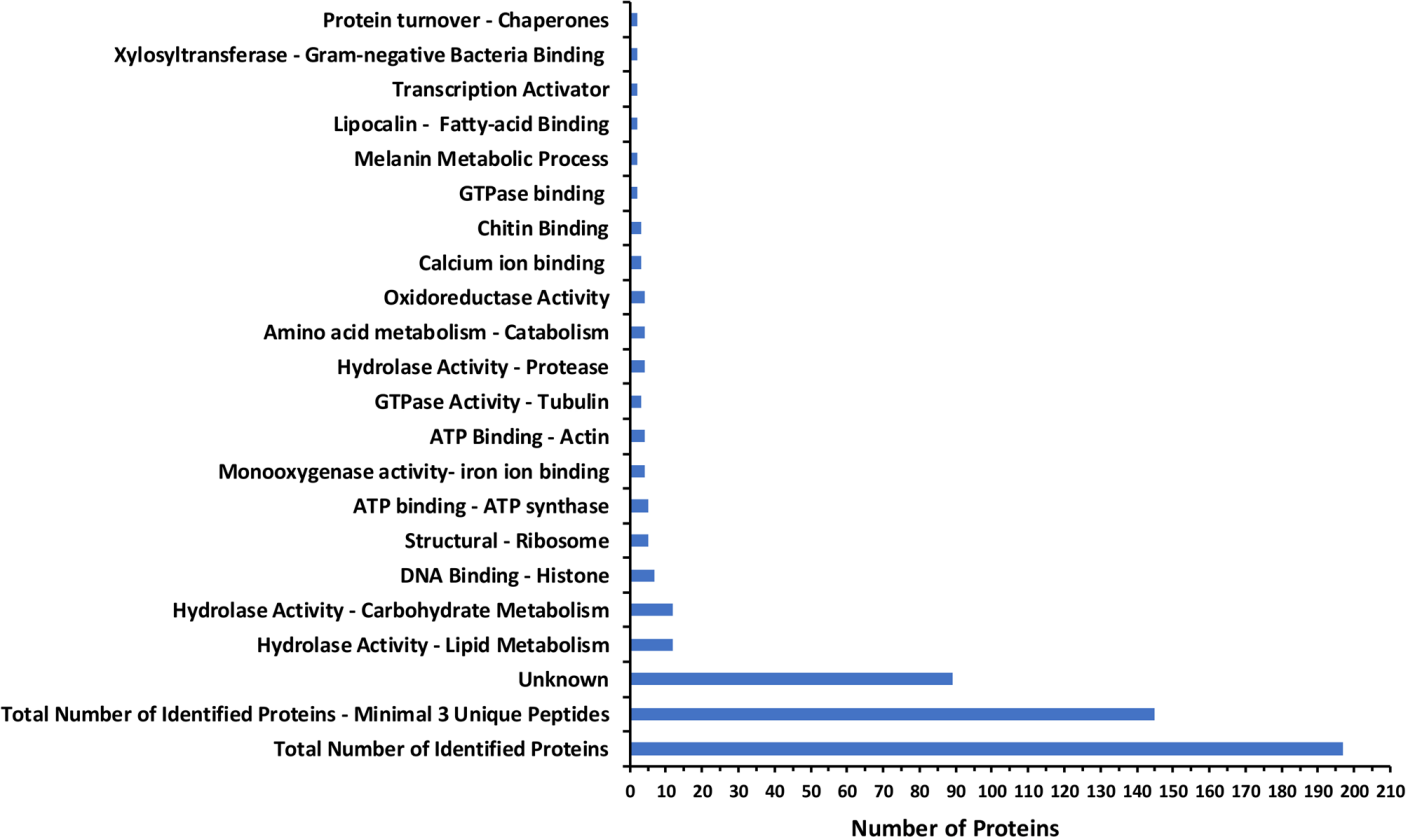
Gene Ontology annotations of proteins identified using a shotgun LC–MS/MS approach from the foam produced by *Mahanarva spectabilis* nymphs infesting *Urochloa brizantha* cv. Marandu, *Urochloa decumbens* cv Basilisk, and elephant grass (*Cenchrus purpureus*) cultivars Pioneiro and Roxo de Botucatu. The most frequent terms for molecular functions and biological processes are indicated. For more details, see the Supplementary Table 1.

A shotgun proteomic approach using LC–MS/MS enabled the identification of 196 proteins in the foam secreted by *M spectabilis* nymphs infesting different forage grasses (Fig. 1). Among them, 143 proteins were detected with at least three unique peptides and an FDR (False Discovery Rate) < 1%. A global overview revealed that only a subset of proteins exhibited identifiable functional domains or characterized molecular signatures (Supplementary Fig. S1a). Notably, approximately 45% of the identified proteins lacked orthologs in the UniProt database and were classified as functionally unknown (Supplementary Fig. S1b). Interestingly, these unknown proteins showed the highest peptide coverage (which does not imply functional relevance, but indicates they were abundant and consistently detected across runs), suggesting their relative abundance and possible importance in foam composition (Supplementary Table S1). The relative protein abundance was positively correlated with the number of peptides identified by LC–MS/MS.

Among the proteins showing homology and Gene Ontology (GO) characterization, the most frequently identified molecular function terms included enzymatic activities such as hydrolase, oxidoreductase, and binding functions (Fig. 1; Supplementary Fig. S1). The key biological processes represented include metabolic processes, response to stress, and transport-related activities. These categories indicate the presence of proteins involved in the insect’s metabolic adaptations, defence mechanisms, and interactions with the external environment. Despite the higher relative abundance of proteins classified as unknown, many proteins were associated with specific GO terms, revealing that certain categories are significantly overrepresented, suggesting their importance in foam composition.

### Changes in protein profiles were genotype-specific

**Fig. 2:**
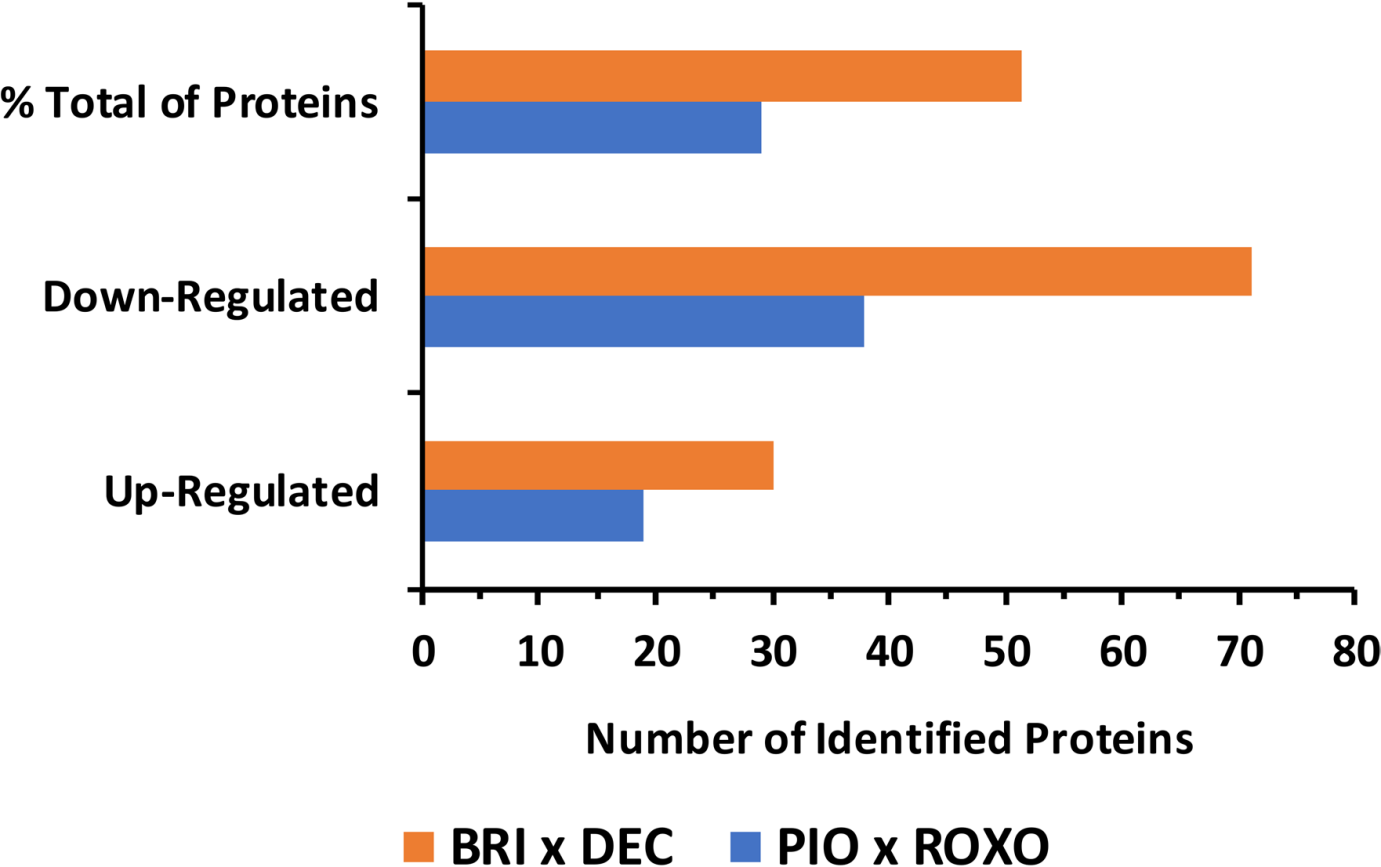
Deregulated proteins identified in the foam produced by *Mahanarva spectabilis* nymphs infesting *Urochloa brizantha* cv. Marandu (BRI), *Urochloa decumbens* cv Basilisk (DEC), and elephant grass (*Cenchrus purpureus*) cultivars Pioneiro (PIO) and Roxo de Botucatu (ROXO). Differentially expressed proteins were categorized according to up- and down-regulation when comparing BRI × DEC (orange bars) and PIO x ROXO (blue bars).

Despite the list of identified proteins in the foam produced by *Mahanarva spectabilis* nymphs varied across different plant genotypes, the profile of differentially expressed proteins was distinct (Fig. 2). The pairwise comparison of BRI vs PIO showed differences in the number of differentially expressed proteins classified as up-regulated and down-regulated, compared to DEC and ROXO. Likewise, the number of down-regulated proteins was higher than the number of up-regulated proteins in both contrasts.

Figure 2 shows the number of deregulated proteins identified in the foam produced by *Mahanarva spectabilis* nymphs infesting plants with different resistance levels. In the BRI x DEC contrast, which compares a resistant (BRI) and a susceptible (DEC) genotype, there is a higher number of differentially expressed proteins, with a notable prevalence of down-regulated proteins compared to up-regulated ones. In the PIO x ROXO contrast, comparing moderately resistant (PIO) and susceptible (ROXO) genotypes, fewer differentially expressed proteins were identified, with a more balanced ratio of up-regulated and down-regulated proteins.

**Fig. 3:**
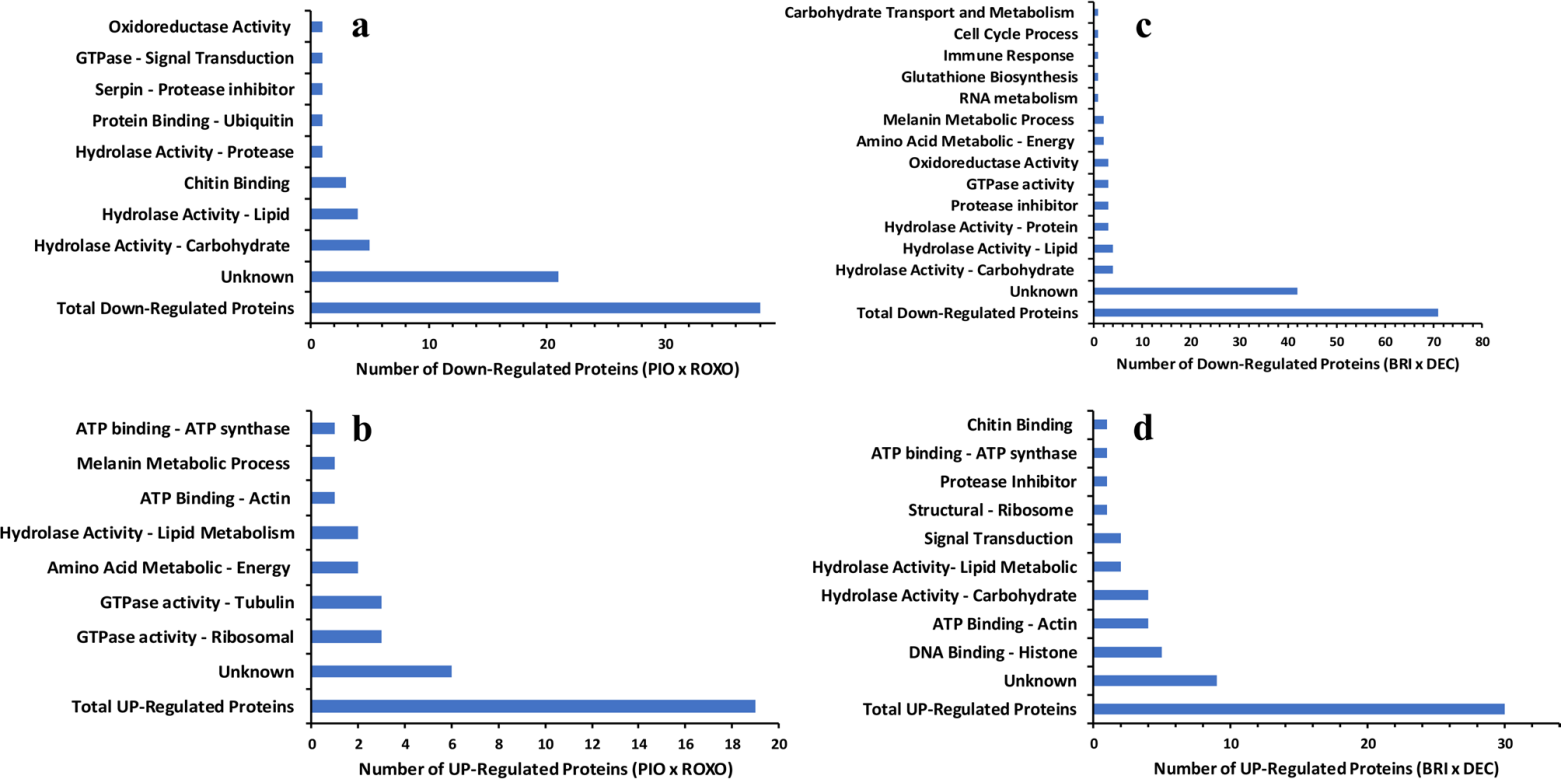
Gene Ontology annotations of deregulated proteins identified from foam produced by *Mahanarva spectabilis* nymphs infesting *Urochloa brizantha* cv. Marandu (BRI), *Urochloa decumbens* cv Basilisk (DEC), and elephant grass (*Cenchrus purpureus*) cultivars Pioneiro (PIO) and Roxo de Botucatu (ROXO). (**a**) (top left side) shows the down-regulated proteins and (**b**) (bottom left side) shows the up-regulated proteins from the pair-wise comparation PIO vs ROXO. (**c**) (top right side) shows the down-regulated proteins and (**d**) (bottom right side) shows the up-regulated proteins from the pair-wise comparation BRI vs DEC.

Figure 3A,B illustrate the GO annotations and the number of deregulated proteins identified from the foam produced by *M. spectabilis* nymphs infesting *Cenchrus purpureus* cultivars Pioneiro (PIO, moderately resistant) and Roxo de Botucatu (ROXO, susceptible). Figure 3A represents the down-regulated proteins in PIO compared to ROXO and indicated several GO terms are associated with metabolic processes, cellular components, and molecular functions. In contrast, Fig. 3B shows the up-regulated proteins in PIO vs ROXO and highlights GO terms linked to stress response, defence mechanisms, and signal transduction pathways.

Figure 3C,D illustrate the GO annotations and the number of deregulated proteins for comparison BRI vs DEC. Figure 3C represents down-regulated proteins indicating GO terms related to metabolic processes, cellular organization, and catalytic activity are affected. In contrast, Fig. 3D shows up-regulated proteins in BRI vs DEC and highlights GO terms associated with defence response, stress adaptation, and signalling pathways.

**Fig. 4:**
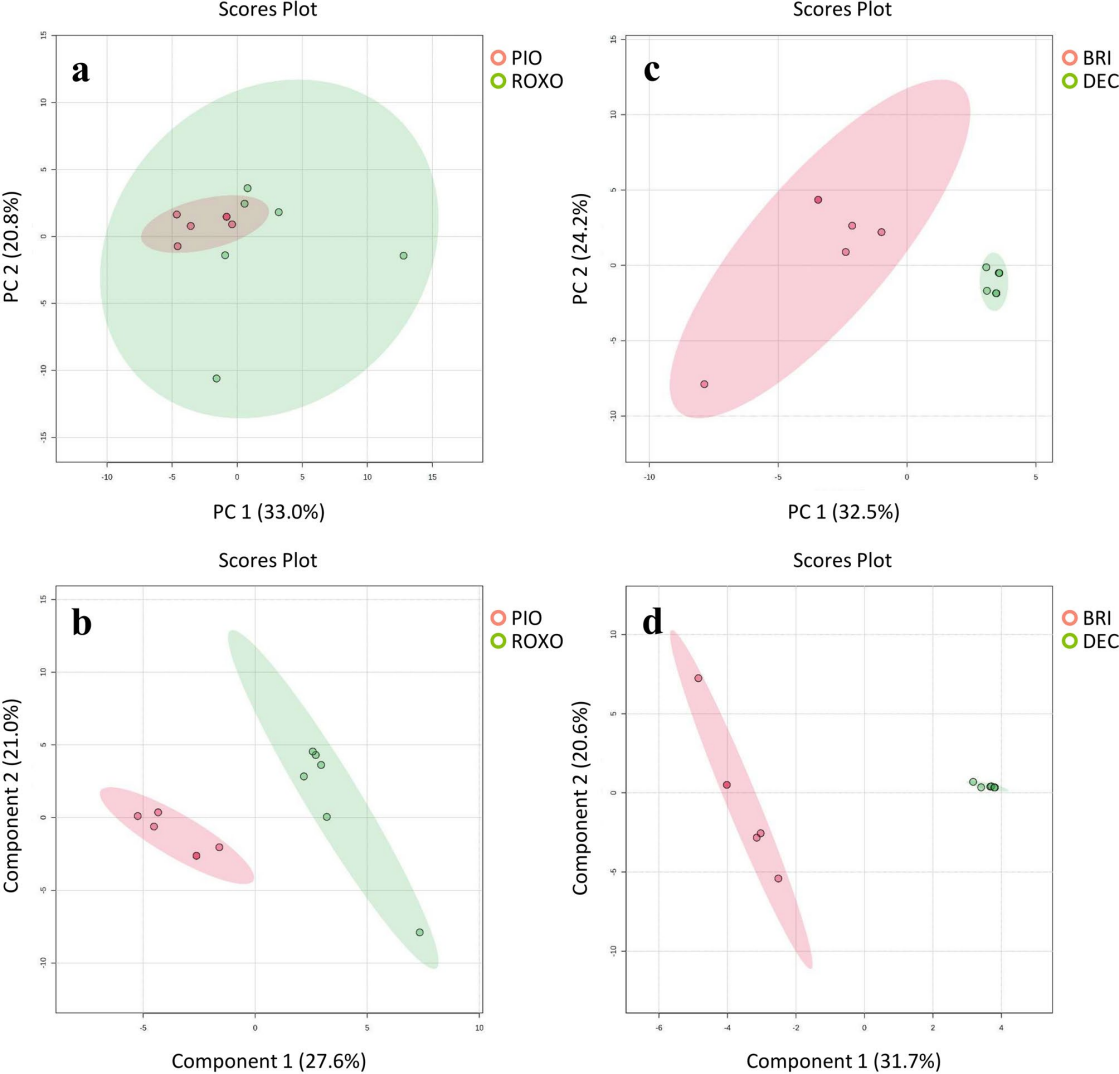
Multivariate analysis of the identified proteins from foam produced by *Mahanarva spectabilis* nymphs infesting *Urochloa brizantha* cv. Marandu (BRI), *Urochloa decumbens* cv Basilisk (DEC), and elephant grass (*Cenchrus purpureus*) cultivars Pioneiro (PIO) and Roxo de Botucatu (ROXO). (**a**) (top left side) shows the Principal Component Analysis (PCA) and (**b**) (bottom left side) indicates the analysis of Two-dimensional Scores Plot by Partial Least Squares Discriminant Analysis (PLS-DA) using the most discriminant proteins from PIO vs ROXO. PIO is considered moderately-resistant while ROXO is susceptible to *M. spectabilis*. (**c**) (top right side) shows the PCA and **d** (bottom right side) indicates the PLS-DA using the most discriminant proteins from BRI × DEC. BRI is resistant while DEC is susceptible to *M. spectabilis*.

Principal Component Analysis (PCA) is an unsupervised technique that reduces the dimensionality of complex proteomic data while preserving variance allowing a better view of patterns, similarities, and differences between samples. In the PCA plot, samples from different groups [e.g., Pioneiro (PIO) and Roxo de Botucatu (ROXO)] are projected onto principal components (PC1 and PC2). It is possible to verify that the samples from the same group cluster closely together, which reflects a consistent proteomic response, despite some separation between the groups (Fig. 4A), as indicated by component 1 (33%), suggesting that genotype affects the foam proteins. PLS-DA (Partial Least Squares Discriminant Analysis) is a supervised technique that maximizes group separation and identifies the variables (proteins) that contribute most to the differences between cultivars. It enhances classification by incorporating class labels, making it useful for distinguishing resistant and susceptible genotypes. A clear separation between groups in the PLS-DA scores plot indicates significant differences in the proteomic response of PIO (moderately resistant) and ROXO (susceptible) to the insect infestation (Fig. 4B).

The PCA plot shows distinct clusters for the BRI (resistant) and DEC (susceptible), indicating that the foam proteomic profiles are significantly different (Fig. 4C). Separation along the principal components, PC2, indicates the degree of variation, with larger distances between groups (31.7%). This was more evident in the PLS-DA plot (Fig. 4D), highlighting the separation of BRI and DEC samples and suggesting distinct proteomic responses, with specific proteins playing crucial roles in resistance or susceptibility.

**Fig. 5:**
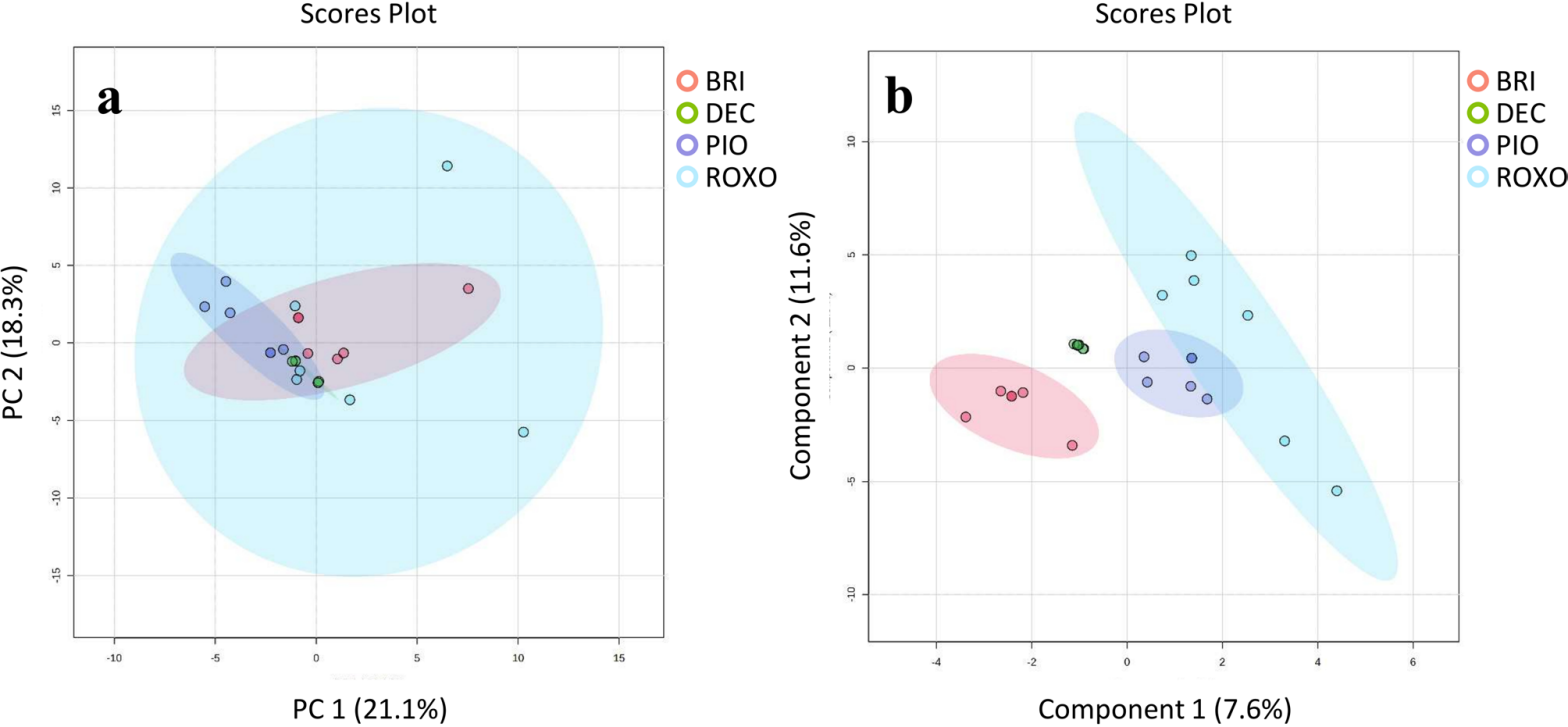
Multivariate analysis comparing the identified proteins from foam produced by *Mahanarva spectabilis* nymphs infesting *Urochloa brizantha* cv. Marandu (BRI), *Urochloa decumbens* cv Basilisk (DEC), and elephant grass (*Cenchrus purpureus*) cultivars Pioneiro (PIO) and Roxo de Botucatu (ROXO). (**a**) (left side) shows the Principal Component Analysis (PCA) and (**b**) (right side) indicates the analysis of Two-dimensional Scores Plot by Partial Least Squares Discriminant Analysis (PLS-DA) using the most discriminant proteins. BRI is resistant, PIO is considered moderately-resistant, while DEC and ROXO are susceptible to *M. spectabilis*.

PCA of the four genotypes (PIO, ROXO, BRI, and DEC) revealed partial overlap among samples, suggesting some shared aspects of foam protein expression across cultivars (Fig. 5A). However, the PLS-DA plot showed low-level separation (7.6%) between moderately resistant/resistant and susceptible groups (Fig. 5B).

### Fold change analysis

**Fig. 6:**
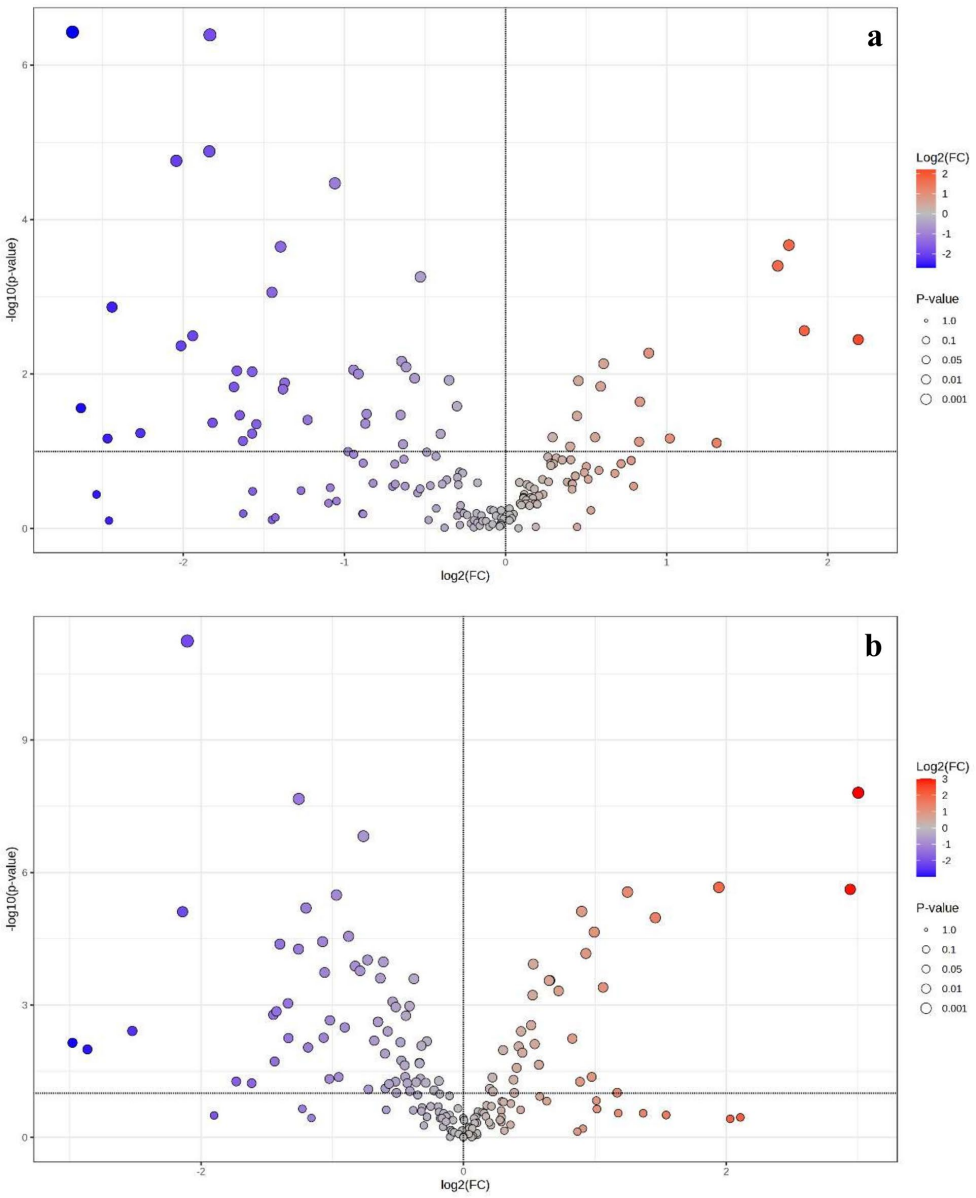
Fold change analysis (FC) of log2 ratio between identified proteins (inferred from transcriptomic data) from foam produced by *Mahanarva spectabilis* nymphs infesting different forage grasses. In (**a**) Volcano Plot (pair-wise comparation PIO × ROXO) representing the up-regulated and down-regulated proteins identified from foam when the nymphs were infesting elephant grass (*Cenchrus purpureus*) cultivars Pioneiro (PIO) and Roxo de Botucatu (ROXO). In (**b**) Volcano Plot (pair-wise comparation BRI × DEC) for foam from *Urochloa brizantha* cv. Marandu (BRI) and *Urochloa decumbens* cv Basilisk (DEC). Positive and negative log2(FC) values indicated up-regulation and down-regulation, respectively. Up-regulated indicates higher protein abundance in PIO related to ROXO or in BRI related to DEC. The term ‘proteins’ is used as an analogy to inferred protein abundance based on transcriptomic data, as gene expression levels often correlate with protein abundance in biological systems.

Volcano plots were used to visualize the log2 fold change (log2FC) in protein abundance between moderately resistant/resistant and susceptible genotypes (Fig. 6). Figure 6A displays the PIO vs. ROXO comparison, while Fig. 6B presents BRI vs. DEC. These volcano plots represent the log2 fold change (FC) of significatively deregulated proteins, where positive values indicate proteins with higher abundance in resistant/moderately genotypes, and negative values indicate higher abundance in susceptible genotypes (Fig. 6A,B). In the PIO vs. ROXO contrast (Fig. 6A), proteins with positive log2FC values were more abundant in the PIO (moderately resistant) genotype, indicating upregulation potentially associated with defensive or stress-response roles. Proteins with negative log2FC values were down-regulated in PIO and thus more abundant in ROXO (susceptible), possibly reflecting unregulated physiological pathways exploited by the insect.

Similarly, in the BRI vs. DEC contrast (Fig. 6B), a greater number of proteins showed negative fold changes, confirming a pattern of overall down-regulation in BRI (resistant) compared to DEC (susceptible). Nonetheless, several upregulated proteins were observed in BRI, which may correspond to specific defence-related mechanisms activated in response to herbivory.

### Functional annotation of proteins identified in the protective foam of *Mahanarva spectabilis* nymphs

Shotgun proteomics analysis enabled the identification of multiple proteins inferred from transcriptomic data in the foam secreted by *M. spectabilis* nymphs. Among these, a subset of five proteins displayed partial functional annotations based on TrEMBL (Translated EMBL) entries, despite being predominantly classified as “uncharacterized proteins” (Supplementary Table S1). These proteins presented high peptide coverage (ranging from 53 to 113 peptides) and large molecular masses (from ~ 45 to 190 kDa), indicating their abundance and potential relevance in the foam matrix. Although SwissProt, Pfam, and KEGG annotations were mostly unavailable, the presence of conserved domains and peptide richness suggests that these proteins may play structural, enzymatic, or protective roles. The annotations available via TrEMBL point to proteins with sequence similarity to entries from *Clastoptera* species, supporting their putative hemipteran origin. Despite lacking precise functional descriptors, the biochemical characteristics of these proteins (high molecular weight and frequent peptide matches—which refers to the alignment of MS/MS-derived peptide spectra with predicted peptides from the transcriptome) suggest roles related to foam stability, antimicrobial defence, or interaction with environmental stressors.

Clustering analysis (Supplementary Fig. S2; Supplementary Fig. S3) grouped genotypes by host plant genus, with distinct clustering of *Cenchrus* and *Urochloa* cultivars. Furthermore, correlation plots based on protein (Supplementary Fig. S4A) and peptide (Supplementary Fig. S4B) quantification across biological replicates revealed strong linear relationships, with Pearson correlation coefficients indicating high reproducibility of the proteomic data. This consistency reinforces the reliability of the experimental design and validates the observed differences between treatments. The similarity between protein and peptide abundance profiles suggests that the quantitative differences identified in the foam proteomes are robust and biologically meaningful. Some of the proteins identified in Supplementary Table S1 may correspond to the differentially expressed genes observed in the PIO vs. ROXO and BRI vs. DEC comparisons (Fig. 6), suggesting a potential link between transcriptomic and proteomic responses in the foam matrix.

### Functional annotation of up-regulated proteins in PIO vs. ROXO

The functional annotation of proteins inferred from up-regulated transcripts in the foam of *M. spectabilis* nymphs associated with the moderately resistant genotype PIO revealed a diverse set of molecular functions and biological processes (Supplementary Table S2). These proteins likely reflect physiological adjustments in response to greater levels of plant resistance, as observed in the PIO vs. ROXO comparison (Fig. 6A). Among the most recurrent functions, there was a strong enrichment of proteins involved in GTPase activity and elongation factor activity, including GTP-binding proteins and components of the translation machinery. These proteins are often associated with cytoskeletal dynamics and protein synthesis regulation. Another relevant category included proteins associated with amino acid metabolism, specifically enzymes with carbamoyltransferase activity, pointing to metabolic reprogramming as part of the insect’s response strategy. Additionally, components of ATP synthesis and energy metabolism (e.g., ATP synthase subunits) were also present. Some up-regulated proteins were linked to hydrolase activity and lipid metabolism, while others were involved in melanin biosynthetic processes, which may relate to oxidative stress management or microbial defence mechanisms within the foam environment.

At the top of the fold-change ranking, the most up-regulated proteins included: Tubulin alpha chains (log2FC = 2.19), with annotations related to GTPase activity, cytoskeleton organization, and microtubule**-**based processes; ATP synthase subunit alpha (log2FC = 1.85), involved in energy production and proton transport; Translation elongation factor Tu (log2FC = 1.69–1.76), linked to protein biosynthesis; Amino acid metabolism enzymes (log2FC ≈ 1.31), annotated with carbamoyltransferase and amino acid binding functions. These proteins correspond to genes exhibiting statistically significant differential expression accordingly to predefined log2 fold-change and *p*-value criteria, as highlighted in the volcano plot (Fig. 6A), reinforcing the link between transcriptomic and inferred proteomic responses in the foam matrix. The log2FC values and functional annotations were maintained and verified for their correspondence with the data in Supplementary Table S2: Tubulin alpha chains (log2FC = 2.19), ATP synthase subunit alpha (log2FC = 1.85), Translation elongation factor Tu (log2FC = 1.69–1.76), and enzymes of amino acid metabolism (log2FC ≈ 1.31).

### Functional annotation of up-regulated proteins in BRI vs. DEC

The functional annotation of proteins inferred from up-regulated transcripts in the foam secreted by *M. spectabilis* nymphs when feeding on the resistant genotype BRI revealed a heterogeneous set of biological functions, indicative of physiological and molecular adaptations likely associated with host-induced stress (Supplementary Table S3). These findings complement the up-regulated protein profiles observed in the PIO vs. ROXO comparison (see Functional annotation of up-regulated proteins in PIO vs. ROXO) and align with the differential expression patterns observed in the BRI vs. DEC comparison (Fig. 6B). Among the most frequent annotations, several proteins were associated with ATP binding and cytoskeletal organization, including actin-related proteins and mitochondrial ATP synthase subunits. These proteins play essential roles in cellular energy regulation**,** vesicle transport, and structural stability. Proteins annotated with DNA binding and nucleosome assembly functions were also up-regulated, including histone components involved in chromatin organization. Notably, proteins with chitin-binding activity and localization in the extracellular region were also identified, implying potential involvement in interactions with microbial populations or structural reinforcement of the foam matrix.

Additional proteins were related to hydrolase activity, particularly those acting on O-glycosyl compounds, pointing to roles in carbohydrate metabolism and possibly in foam remodeling or degradation of plant-derived molecules. At the top of the fold-change distribution, the most up-regulated proteins included: Actin-related protein 1 (log2FC = 3.00), associated with cytoskeletal organization; ATP synthase subunit alpha, mitochondrial (log2FC = 2.94), involved in energy production; Actin, muscle isoform (log2FC = 1.94), linked to structural stability; and Rac GTPase-activating protein 1 (log2FC = 1.46), related to GTPase activity and cellular signaling. These proteins correspond to genes exhibiting statistically significant differential expression highlighted in the volcano plot (Fig. 6B), such as Actin-related protein 1 (log2FC = 3.00) and ATP synthase subunit alpha (log2FC = 2.94), reinforcing the link between transcriptomic and inferred proteomic responses in the foam matrix. The log2FC values and functional annotations were maintained and verified for their correspondence with the data in Supplementary Table S3: Actin-related protein 1 (log2FC = 3.00), ATP synthase subunit alpha (log2FC = 2.94), Actin, muscle isoform (log2FC = 1.94), and Rac GTPase-activating protein 1 (log2FC = 1.46).

### Functional annotation of down-regulated proteins in PIO vs. ROXO

The functional annotation of proteins inferred from significantly down-regulated transcripts in the foam produced by *M. spectabilis* nymphs infesting the moderately resistant genotype PIO revealed a notable reduction in enzymes involved in carbohydrate and lipid metabolism, extracellular interactions, and oxidative stress response (Supplementary Table S4). These findings complement the differential expression patterns observed in the PIO vs. ROXO comparison (Fig. 6A) and reflect physiological adaptations to host plant moderate resistance. The most frequently suppressed functional class included hydrolases that act on O-glycosyl compounds, enzymes typically associated with the breakdown of complex carbohydrates (e.g., cellulases, glycosidases). Several proteins involved in lipid metabolism, such as phosphoric diester hydrolases and triglyceride lipases, were also down-regulated. These enzymes are generally involved in membrane remodeling, energy mobilization, or secretion dynamics. Another notable group of down-regulated proteins was annotated with chitin-binding activity and localization in the extracellular region. Additionally, proteins related to serine-type endopeptidase activity (e.g., e.g., Serpin-like secreted protease inhibitor, log2FC = − 2.27)] and superoxide dismutase (SOD) (a key antioxidant enzyme) were also reduced in abundance.

The proteins with the highest fold-change suppression (most negative log2FC values) included: Inter-alpha-trypsin inhibitor heavy chain H4-like (log2FC = − 2.69), possibly involved in extracellular matrix stabilization or protease inhibition; a hydrolase with activity on O-glycosyl compounds (log2FC = − 2.63), likely involved in carbohydrate degradation and down-regulated in resistant host conditions; a serpin-like secreted protease inhibitor (log2FC = − 2.27), suggesting reduced protease regulation or defensive secretion; and a triglyceride lipase (log2FC = -1.83), potentially involved in lipid mobilization or nutrient processing. These proteins correspond to genes exhibiting statistically significant differential expression accordingly to predefined log2 fold-change and *p*-value criteria, as highlighted in the volcano plot (Fig. 6A), reinforcing the link between transcriptomic and inferred proteomic responses in the foam matrix. The log2FC values and functional annotations were maintained and verified for their correspondence with the data in Supplementary Table S4: Inter-alpha-trypsin inhibitor heavy chain H4-like (log2FC = − 2.69), hydrolase with activity on O-glycosyl compounds (log2FC = − 2.63), serpin-like secreted protease inhibitor (log2FC = − 2.27), and triglyceride lipase (log2FC = − 1.83).

### Functional annotation of down-regulated proteins in BRI vs. DEC

The functional analysis of proteins inferred from significantly down-regulated transcripts in the foam of *M. spectabilis* nymphs infesting the resistant genotype BRI indicates suppression of diverse biological processes, particularly those linked to hydrolytic activity, nucleotide metabolism, and lipid processing (Supplementary Table S5). These findings complement the differential expression patterns observed in the BRI vs. DEC comparison (Fig. 6B) and reflect physiological adaptations to host plant resistance. Several down-regulated proteins were annotated with hydrolase activity, especially those acting on O-glycosyl compounds and nucleotides (e.g., a hydrolase related to glycoside hydrolysis, log2FC = − 2.98). These enzymes are typically involved in degradation of carbohydrates, nucleic acids, or lipids. Proteins associated with melanin biosynthesis and oxidative pigment metabolism were also down-regulated, including enzymes related to phenol and aromatic compound processing (e.g., a protein annotated with melanin biosynthetic processes, log2FC = − 2.52). Additionally, annotations related to RNA modification, such as pseudouridine synthases (log2FC = − 1.73), point to possible reductions in RNA maturation or stability in BRI foam.

Among the proteins with the strongest down-regulation (most negative log2FC values), the following stand out: a hydrolase related to glycoside hydrolysis (log2FC = − 2.98), possibly involved in carbohydrate degradation and nutrient mobilization; a protein annotated with melanin biosynthetic processes (log2FC = − 2.52), related to oxidative stress buffering or foam coloration; a pseudouridine synthase (log2FC = − 1.73), implicated in RNA processing and modification; and a protein involved in lipid metabolic processes (log2FC = − 1.42), likely relevant to membrane or secretion remodeling. These proteins correspond to genes exhibiting statistically significant differential expression accordingly to predefined log2 fold-change and *p*-value criteria, as highlighted in the volcano plot (Fig. 6B), reinforcing the link between transcriptomic and inferred proteomic responses in the foam matrix. The log2FC values and functional annotations were maintained and verified for their correspondence with the data in Supplementary Table S5: a hydrolase related to glycoside hydrolysis (log2FC = -2.98), a protein annotated with melanin biosynthetic processes (log2FC = − 2.52), a pseudouridine synthase (log2FC = − 1.73), and a protein involved in lipid metabolic processes (log2FC = − 1.42).

## Discussion

The characterization of the foam proteome secreted by *M. spectabilis* nymphs offers a unique opportunity to understand the molecular mechanisms underlying the interaction between spittlebug and forage grasses. This knowledge is essential to develop new strategies to disrupt foam-mediated protection and to enhance the efficacy of plant resistance traits against spittlebugs, which is especially important due to the economic losses caused by *M. spectabilis* in tropical forage systems, with can led to forage yield reductions up to 43%^16^. This integrative approach bridges molecular entomology and applied plant protection, providing a valuable foundation for the design of novel biotechnological products to pest management.

This proteomic survey of *M. spectabilis* foam represents, to our knowledge, the most comprehensive characterization of the proteinaceous components of this unique secretion. The analysis highlights the complexity of the foam proteome, identifying 196 proteins, of which 45% lack homologs in reference databases and are classified as functionally unknown. These uncharacterized proteins, with high peptide coverage (53–113 peptides), suggest the evolution of lineage-specific or foam-specific molecules potentially involved in structural maintenance, microbial defence, or biochemical signalling within the foam microenvironment^5^. Recent genomic and transcriptomic studies, such as Zhang et al.^17^ on *Callitettix versicolor*, have begun to elucidate the genetic basis of foam secretion, identifying 606 species-specific gene families and 66 expanded gene families associated with carbohydrate and lipid metabolism, which are essential for foam production. These findings underscore the conserved nature of metabolic pathways supporting foam in spittlebugs and provide a foundation for interpreting our proteomic data.

Similar to the foam of *Poophilus costalis*, which contains chitinases with antifungal properties^5^, our findings suggest that *M. spectabilis* foam proteins may contribute to microbial defence, complementing their structural roles. Similar proteomic approaches have characterized bioactive proteins in other insect secretions, such as the salivary proteome of aphid (*Macrosiphum euphorbiae*)^18^ and silk protein mixtures in caterpillar (*Bombyx mori*)^19^, highlighting the potential of such secretions as adaptive interfaces in insect physiology. In the foam produced by *M. spectabilis*, the detection of protease inhibitors has been associated with modulation of microbial proliferation and suppression of exogenous proteases, thereby reducing degradation of secreted foam components. We also observed proteins with predicted extracellular localization, which reinforces their likely involvement in protective biochemical interactions occurring at the foam surface, where microbial exposure is highest. All of this suggests that *M. spectabilis* foam shares adaptive functions, like microbial defence, but exhibits unique genotype-dependent modulation, distinguishing it from other insect secretions. The presence of hydrolases, oxidoreductases, and binding proteins suggests active biochemical interactions occurring within the foam (i.e., biochemical and structural associations among secreted proteins and between these proteins and the aqueous matrix), potentially linked to microbial regulation, detoxification, and nutrient recycling. Additionally, proteins related to chitin-binding, ATP-binding, and stress responses were frequently identified, indicating roles in maintaining foam structure, supporting energy homeostasis, and mediating defence processes. Together, these protein functions align with the ecological role of the foam as a stable and protective microenvironment essential for nymphal development. Since the nymphal stage of *M. spectabilis* is particularly vulnerable due to its sessile nature and exposure to environmental stressors^20^, the foam likely evolved as a multifunctional secretion combining structural, biochemical, and immunological protection. Among the proteins potentially associated with immune-related processes, we identified chitin-binding proteins and also several proteins contained domains related to oxidoreductase activity, protease inhibition and extracellular stabilization. Oxidoreductases are commonly involved in detoxification and oxidative stress control, mechanisms frequently activated during immune response.

Oxidoreductases and the presence of stress-response proteins suggest a protective role against environmental stressors. The foam’s surface constitutes a continuously moistened biochemical interface with the plant and soil, making it susceptible to microbial colonization. The identified proteins, including protease inhibitors, oxidoreductases, and apolipophorin-like proteins, are consistent with a need for antimicrobial modulation at this interface. In insects, protease inhibitors can inactivate exogenous microbial proteases^21,22^, oxidoreductases help manage oxidative imbalance associated with microbial challenge^23^, and apolipophorins are involved in innate immune signalling and lipid-mediated defence^24^. Thus, their abundance in the foam proteome likely reflects an adaptation to protect the nymph’s microenvironment from potential pathogens. Antioxidant enzymes, such as peroxidases, could help mitigate oxidative damage caused by exposure to environmental factors. Enzymes related to metabolic processes indicate that the foam might support the insect’s physiological functions by modulating nutrient availability and waste processing. Proteins involved in binding activities (e.g., ATP and ion binding) could contribute to the structural stability of the foam matrix, potentially enhancing its physical properties as a protective barrier. Hydrolase enzymes may play a role in degrading plant compounds or modifying the foam composition for adaptability. Some proteins may be involved in plant–insect interactions, helping the nymphs manipulate signals to suppress plant defence or optimize nutrient uptake.

The presence of conserved domains such as lipocalin-like folds, peroxidase/oxidoreductase signatures and serine-protease inhibitor motifs suggest that these proteins participate in three distinct functional axes: (i) stabilization of the foam matrix through lipid-binding scaffolds; (ii) modulation of oxidative processes via redox enzymes; and (iii) regulation of proteolytic activity through inhibitory domains. These functions are aligned with insect defence and interface stabilization of the foam. The presence of these proteins in the foam matrix supports the hypothesis that the secretion is not only a physical barrier but also a biochemically active environment. This aligns with previous reports indicating that insect-produced foam can regulate microenvironmental conditions (humidity and temperature), offer protection from predators and parasitoids, and potentially contain compounds with antimicrobial properties. Further biochemical and structural studies, including domain prediction and functional assays, are warranted to clarify the precise roles of these abundant, yet functionally ambiguous, proteins within the protective foam.

Our findings reveal a complex proteomic modulation in the foam secreted by *M. spectabilis* nymphs in response to different host plant genotypes, especially when comparing moderately resistant/resistant (PIO and BRI) with susceptible (ROXO and DEC) cultivars. The observed patterns of up- and down-regulated proteins suggest that host resistance imposes significant metabolic and physiological challenges on the insect, resulting in adaptive biochemical responses. Proteomic profiling, visualized through volcano plots (PIO vs. ROXO; BRI vs. DEC), demonstrates distinct patterns of protein expression that reflect physiological adaptations and metabolic adjustments to plant-induced stress. In both moderately resistant/resistant genotypes, up-regulated proteins were primarily associated with cytoskeletal organization (e.g., actin, tubulin alpha chains with log2FC = 2.19 in PIO, actin-related protein 1 with log2FC = 3.00 in BRI), ATP-dependent processes (e.g., ATP synthase subunit alpha with log2FC = 1.85 in PIO and 2.94 in BRI), and translation machinery components (e.g., translation elongation factor Tu with log2FC = 1.69–1.76 in PIO). These functions suggest that nymphs respond to plant resistance by enhancing cellular restructuring, energy mobilization, and protein synthesis to maintain homeostasis and sustain secretory activity under adverse conditions^25^.

A notable shared feature across moderately resistant/resistant genotypes was the up-regulation of proteins associated with cytoskeletal remodeling, energy production n (e.g., ATP synthase subunits), and translation machinery. These components likely support structural integrity and cellular resilience, enabling nymphs to maintain viability under stressful conditions. The presence of elongation factors, ATP synthase subunits, and actin-related proteins in both PIO and BRI highlights a conserved strategy involving activation of stress-response pathways and cytoskeletal dynamics, potentially enabling nymphs to maintain viability under stressful conditions. These findings, revealed by our proteomic analysis, expand the current understanding beyond previously described genotype-dependent foam profiles. Simultaneously, down-regulated proteins were often hydrolases and enzymes linked to lipid and carbohydrate metabolism. This suppression implies a potential disruption of foam structural maintenance or degradation of host-derived substrates. The down-regulation of metabolic enzymes in moderately resistant/resistant genotypes could reduce foam’s adhesive or antimicrobial properties, potentially limiting predator defence, warranting further investigation. These metabolic constraints may affect nutrient processing, especially in foam-associated microenvironments, thereby limiting nymph development and survival.

Our proteomic analysis revealed a significant presence of proteins involved in carbohydrate and lipid metabolism, such as O-glycosyl hydrolases and lipases, which are essential for maintaining foam structure and function. These findings are consistent with genomic insights from Zhang et al.^17^, who identified expanded gene families in *C. versicolor* associated with these metabolic pathways, suggesting a conserved mechanism for foam production across spittlebug species. Concurrently, both resistant and moderately resistant genotypes exhibited down-regulation of proteins related to carbohydrate and lipid metabolism, such as O-glycosyl hydrolases (log2FC = − 2.63 in PIO, − 1.44 in BRI), triglyceride lipases (log2FC = − 1.83 in PIO), and enzymes involved in pigment biosynthesis and oxidative balance (e.g., melanin biosynthesis proteins with log2FC = − 2.52 in BRI). The suppression of hydrolases, serpins (log2FC = − 2.27 in PIO), and superoxide dismutase (SOD) suggests a reduction in extracellular degradation capacity, redox control, and regulatory proteolysis—functions that typically support nutrient acquisition and foam stability in favorable environments^26^ like ROXO and DEC. This suppression implies a disruption of foam structural maintenance or degradation of host-derived substrates, potentially limiting nutrient processing in foam-associated microenvironments and thereby constraining nymph development and survival. These metabolic constraints align with the hypothesis that resistant genotypes reduce the nutritional or biochemical suitability of the host plant, as supported by Cardona et al.^20^.

Interestingly, unique responses were also observed. In BRI, chromatin-associated proteins (e.g., histones, pseudouridine synthases with log2FC = − 1.73) were differentially expressed, pointing to changes in transcriptional or post-transcriptional regulation potentially affecting gene expression or RNA processing in response to host-induced stress. In PIO, the up-regulation of proteins involved in melanin biosynthesis and chitin-binding proteins may reflect immunological or protective adaptations, possibly enhancing foam stability or microbial resistance^5^. These distinct proteomic signatures, support the hypothesis that resistant host plants modulate the composition of *M. spectabilis* foam as part of a plant–insect interaction mechanism. From the insect’s perspective, the up-regulation of cytoskeletal, energetic, and translational proteins represents an attempt to compensate for physiological stress, while the down-regulation of metabolic enzymes and extracellular effectors likely reflects limited resource availability or direct suppression by plant-derived factors, such as secondary metabolites or defence compounds.

The influence of host plant genotype on foam composition, as observed in our study, underscores the foam’s role as a dynamic interface mediating plant–insect interaction, which is aligned with the view of the foam as a functional interface mediating insect-plant interaction. The observed proteomic adaptations, such as up-regulation of cytoskeletal proteins in resistant genotypes, may influence the long-term survival and evolution of *M. spectabilis*, potentially expanding its host range or reflecting co-evolutionary pressures with resistant plants, suggesting dynamic interactions in tropical ecosystems. This is supported by recent genomic studies, such as Zhang et al.^17^, which demonstrate that foam secretion is genetically regulated, with specific gene families playing critical roles in metabolism. Thus, the modulation of foam by resistant genotypes may involve complex interactions between insect genetics and plant biochemical responses^17^. Studies on other spittlebugs reinforce that foam not only acts as a physical barrier but also contains active compounds potentially involved in thermal insulation, pathogen defence, and detoxification^5,6^.

Altogether, our results support the hypothesis that resistant genotypes not only reduce the nutritional or biochemical suitability of the host plant but also modulate insect secretions such as foam. The influence of host plant genotype on foam composition, as observed here, aligns with the view of foam as a functional interface mediating insect-plant interactions, consistent with observations by Auad et al.^12^. The foam’s proteome, thus, may act as a sensitive indicator of host-imposed stress and insect physiological state. The presence of distinct resistance-associated proteomic profiles reinforces the concept of foam as a key trait in insect survival, and potentially, a novel target for biocontrol or breeding strategies^27^.

## Conclusion

This study provides the first comprehensive proteomic characterization of foam secreted by *M. spectabilis* nymphs, revealing a complex and dynamic molecular interface shaped by host plant resistance. Comparative analysis across resistant (*Urochloa brizantha* cv. Marandu), moderately resistant (*Cenchrus purpureus* cv. Pioneiro) and susceptible (*C. Purpureus* cv. Roxo de Botucatu and *U. decumbens* cv. Basilisk) grass genotypes demonstrated genotype-dependent modulation, with resistant/moderately resistant hosts inducing up-regulation of cytoskeletal and energy-related proteins (e.g., tubulin alpha and ATP synthase) and suppression of metabolic enzymes (e.g., O-glycosyl hydrolases). These results indicate that resistant/moderately resistant genotypes impose metabolic constraints on the insect, limiting nutrient processing and foam functionality, while triggering adaptive stress responses. The foam’s proteomic plasticity highlights its role as a sensitive indicator of plant–insect interactions, with implications for *M. spectabilis* survival and co-evolutionary dynamics in tropical forage systems. These findings contribute to our understanding of plant–insect interactions and the role of foam proteins in insect adaptation and survival. They also offer novel targets to control *M. spectabilis*, such as RNAi-based biocontrol or resistant cultivar breeding targeting key foam proteins. Future research should focus on functional validation of uncharacterized proteins and integration with genomic data to elucidate regulatory mechanisms, paving the way for sustainable strategies to mitigate spittlebug damage in agriculture.

## Materials and methods

### Assays of plant infestation and foam sampling

Foam samples produced by fourth and fifth instar nymphs of *Mahanarva spectabilis* were obtained at Embrapa Dairy Cattle (Juiz de Fora, MG, Brazil) as follows. *(1) Plant material:* The vegetative propagules of *Cenchrus purpureus* (syn. *Pennisetum purpureum*) cv. Pioneiro (here designated as PIO) and cv. Roxo de Botucatu (ROXO) were collected as described by Barros et al.^28^. PIO shows statistically superior antibiosis-type resistance when comparing to ROXO^7,8^ and PIO was considered here as moderately resistant in comparison with ROXO, which is a susceptible genotype. Seeds of *Urochloa brizantha* (syn. *Brachiaria brizantha*) cv. Marandu (BRI) and *Urochloa decumbens* (syn. *Brachiaria decumbens*) cv. Basilisk (DEC), which are considered as resistant and susceptible^9,10^, were obtained at Embrapa Dairy Cattle. Supplementary Fig. S5 illustrates the different levels of resistance against nymphs of *Mahanarva* sp. (measured as percentage of nymphal survival where lower survival indicates higher resistance level) in the four cultivars evaluated here. Vegetative propagules and seeds of the cultivars were propagated (3 to 5 plants per pot) in 1 L pots containing a soil mixture and commercially available Plantmax substrate. *(2) Obtaining nymphs:* Adults of *M. spectabilis* were collected at the Embrapa Dairy Cattle experimental field (21°33′22′′ S and 43°16′15′′ W) and kept in *Urochloa ruzizienses* (syn. *Brachiaria ruziziensis*) cv. Kennedy that is highly susceptible to *M. spectabilis*. Plants were kept in cylindrical acrylic cages (30 × 30 × 60 cm^3^) where the base of the plant and the floor of the cage were covered with moist gaze, which was used as an oviposition substrate for the spittlebugs. The eggs deposited on the substrate were collected, washed with water on a series of sieves, placed in Petri dishes (10 cm diameter) lined with filter paper and incubated in BOD type climatic chamber (28 ± 2 °C, 70 ± 10% relative humidity, 14 h photophase). Once the eggs reached the S4 phase (close to the nymph hatching point), they were placed in *U. ruzizienses* cv. Kennedy for nymph development. After 35 days, the fourth and fifth instar nymphs were collected. *(3) Foam sampling:* Ten nymphs were placed on 12 to 16-months-old plants of BRI, DEC, PIO and ROXO covered with organza bags to prevent their escape, maintained at 25º C and 70% relative humidity. After 24 h of nymph infestation, twelve foam samples (three biological replicates per genotype) were collected using a spatula and transferred to a 50 ml Falcon tube. Each replicate contained foam collected from plants of three pots. Immediately after being collected, the foam was frozen in liquid nitrogen and kept at -80 °C. The samples were then transported (in a styrofoam box containing dry ice) to the Center of Analysis of Biomolecules (NuBioMol) at the Federal University of Viçosa (Viçosa, MG, Brazil). The foam samples were centrifuged at 10,000 × g for 10 min and transferred to new 50 mL Falcon tubes. The resulting supernatants were then transferred to 1.5 mL microtubes. Each sample volume was standardized to 500 µL using Milli-Q water, and the samples were dried in a speed vacuum concentrator (Concentrator Plus, Eppendorf, Germany).

### Protein extraction and quantification

Proteins were solubilized in 100 µL of 10% sodium dodecyl sulfate (SDS) buffer. After homogenization, the samples were centrifuged at 12,000 × g for 15 min at 4 °C, and the supernatants were collected. Protein concentration was determined using the bicinchoninic acid (BCA) assay, with bovine serum albumin (BSA) as the standard, measured at 562 nm using a microplate reader spectrophotometer (SpectraMax M2, Molecular Devices, USA).

### Denaturing electrophoresis (SDS-PAGE – “Short Run”)

Aliquots containing 50 µg of protein from each sample were loaded onto a 12% polyacrylamide gel (10 cm × 10 cm, 1.5 mm thick) under denaturing conditions, following the protocol described by Laemmli^29^. Electrophoresis was performed using a “short run” method, allowing protein separation in approximately 30 min at 120 V. After electrophoresis, gels were fixed in 10% methanol and 5% acetic acid for 2 h and stained with Coomassie Brilliant Blue G250.

### In-gel digestion

Protein bands containing all stained proteins were excised from the SDS-PAGE gels, segmented uniformly, and transferred to 1.5 mL microtubes. Gel fragments were destained by successive washes with 50% acetonitrile and 25 mM ammonium bicarbonate at room temperature, as described by Shevchenko et al.^30^. The destaining solution was removed, and the gels were dehydrated twice with 100% acetonitrile for 5 min each, then dried in a speed vacuum concentrator for 15 min. Proteins were reduced in a solution of 65 mM DTT (Dithiothreitol) and 100 mM ammonium bicarbonate at 56 °C for 30 min in a thermostatic bath. Subsequently, alkylation was performed using 200 mM iodoacetamide and 100 mM ammonium bicarbonate for 30 min at room temperature, protected from light. The gels were then washed twice with 100 mM ammonium bicarbonate for 10 min and dehydrated with 100% acetonitrile for 5 min. This washing cycle was repeated three times before drying in a vacuum concentrator (SpeedVac) (Eppendorf Concentrator Plus, Germany) for 15 min to ensure complete solvent evaporation.

For enzymatic digestion, the gels were rehydrated with 20 µL of a solution containing 25 ng µL⁻^1^ trypsin (Proteomics grade, Sigma-Aldrich, USA), 40 mM ammonium bicarbonate (pH 8.0), and 10% acetonitrile. The gels were incubated on ice for 45 min to facilitate enzyme penetration. Next, 50 µL of 50 mM ammonium bicarbonate was added, and the samples were incubated overnight at 37 °C in a thermostatic bath.

Tryptic peptides were extracted from the gel slices by adding extraction buffer and applying ultrasonic agitation for 10 min, allowing peptide diffusion into the solvent. Samples were then centrifuged at 200×*g* for 2 min, and the supernatant was transferred to new microtubes. To increase peptide recovery, 40 µL of a solution containing 5% formic acid and 50% acetonitrile was added to the remaining gel fragments, followed by a second cycle of ultrasonic agitation for 10 min. After centrifugation, the recovered supernatants were pooled. The peptide extraction procedure was repeated by adding fresh extraction buffer to the remaining gel fragments, followed by ultrasonic agitation and centrifugation, after which the additional supernatant was collected and pooled with the previous extract. The peptide solutions were dried in a speed vacuum concentrator (SpeedVac) (Eppendorf Concentrator Plus, Germany) and stored at -20 °C.

### Protein identification by LC–MS/MS

The dried peptide samples were reconstituted in 50 µL of 0.1% formic acid and analysed using a nano-UHPLC system (UltiMate R 3000, Dionex, San Jose, USA) equipped with an Acclaim PepMap100 C18 Nano-Trap column (100 µm i.d. × 20 mm, 5 µm, 100 Å; Thermo Scientific, Waltham, MA, USA) and an analytical Acclaim PepMap100 C18 RSLC column (75 µm i.d. × 150 mm, 2 µm, 100 Å; Thermo Scientific, Waltham, MA, USA), in tandem with the trap column, operating at a constant flow rate of 0.3 µL min⁻^1^.

The solvents used were: (A) 0.1% formic acid (HPLC grade, JTBaker, Mexico) and (B) 80% acetonitrile with 0.1% formic acid (HPLC grade, JTBaker, Mexico). A multistep gradient was applied as follows: an initial conditioning step with 3.8% B for 3 min, followed by a linear increase from 3.8% to 30% B over 120 min, then from 30 to 55% B by 150 min. A final ramp to 99% B was completed at 162 min, followed by reconditioning with 3.8% B until 180 min.

Spectral data were acquired using a Q-Exactive mass spectrometer (Thermo Scientific, Bremen, Germany) operating in full-scan/MS2 mode. The nanospray flex ion source (Thermo Scientific) was set to 3.8 kV in positive mode, with a capillary temperature of 250 °C and S-lens RF level set to 55. The Data-Dependent Acquisition (DDA) method was configured to select the top 12 ions with charge states between + 2 and + 4 within a 1.2 m/z isolation window. Survey scans were acquired at a resolution of 70,000 with a mass ran.

### Transcriptome sequencing and protein database construction

Total RNA extracted from *M. spectabilis* was used to generate a transcriptomic database for protein identification. After RNA extraction using TRIzol reagent^31^, RNA quality was assessed using NanoDrop (Thermo Fisher Scientific, USA), Qubit 2.0 fluorometer (Thermo Fisher Scientific, USA), and Agilent 2100 Bioanalyzer (Agilent Technologies, USA). Only high-quality RNA samples were processed for mRNA library construction. Polyadenylated mRNA was isolated using oligo(dT) magnetic beads, randomly fragmented, and reverse-transcribed into cDNA using random hexamers. Second-strand synthesis was performed, and the resulting cDNA was purified, end-repaired, A-tailed, and ligated to adapters. Fragments of 300–400 bp were selected and PCR-amplified to generate cDNA libraries. Libraries were quantified with Qubit and Agilent Bioanalyzer, and sequenced using the Illumina platform.

Raw reads were processed to remove adaptors and low-quality sequences, generating clean FASTQ files. High-quality reads were assembled de novo into contigs using optimized transcriptome assemblers. Assembled contigs were then functionally annotated by aligning predicted protein sequences against public databases (NR—Non-redundant Protein Database, Swiss-Prot—Curated protein database of UniProt, COG—Clusters of Orthologous Genes, KOG—Eukaryotic Orthologous Groups, and KEGG—Kyoto Encyclopedia of Genes and Genomes^32–34^) using DIAMOND. Gene Ontology (GO) terms were assigned via InterProScan and Pfam domains were identified using HMMER, facilitating the construction of a comprehensive reference protein database for downstream proteomic analysis.

### Bioinformatics and statistical analysis

Raw mass spectrometry data were processed and analysed using a label-free quantification (LFQ) workflow. Protein identification was performed using a target-decoy strategy with a false discovery rate (FDR) cutoff of 1% at the peptide and protein levels. Proteins identified with at least three unique peptides were considered for downstream analyses.

Functional annotation of proteins was conducted using multiple bioinformatics resources. Gene Ontology (GO) terms for Molecular Function, Biological Process, and Cellular Component were assigned based on BLASTp searches against UniProtKB, followed by classification through InterProScan and eggNOG-mapper (v2). KEGG Orthology (KO) and Pfam domains were also retrieved when available to refine functional categorization.

Differential expression analysis between treatments was performed using log2 fold-change (log2FC) calculations and raw *p*-values derived from spectral count comparisons. Proteins with log2FC > 1 or < -1 and *p* < 0.05 were considered significantly up- or down-regulated, respectively. Volcano plots were generated to visualize these expression patterns.

Multivariate statistical analyses were applied to assess global patterns of proteome variation. Principal Component Analysis (PCA) and Partial Least Squares Discriminant Analysis (PLS-DA) were carried out using the MetaboAnalyst platform. Clustering and heatmap visualizations were based on Z-score-normalized LFQ intensities, and one-way ANOVA was applied to detect statistically significant differences across genotypes.

Correlation analyses were performed between peptide and protein quantification datasets to evaluate consistency and reproducibility across biological replicates. Pearson correlation coefficients and regression plots were calculated using built-in R functions and visualized to validate the robustness of the proteomic measurements.

## Supplementary Information

Below is the link to the electronic supplementary material.

Supplementary Material 1

## Data Availability

The datasets used and/or analysed during the current study are available from the corresponding author on reasonable request.
