# Supplementary material for: The adaptive nature of the foam proteome produced by Mahanarva spectabilis (Hemiptera: Cercopidae) when infesting forage grasses with different levels of antibiosis-type resistance
Source: Sci Rep. 2026 Feb 3;16:7114. doi: 10.1038/s41598-026-36784-9 (PMC12921220; doi:10.1038/s41598-026-36784-9)
Supplement: Supplementary file 1 — Supplementary Material 1 [file 41598_2026_36784_MOESM1_ESM.pdf]

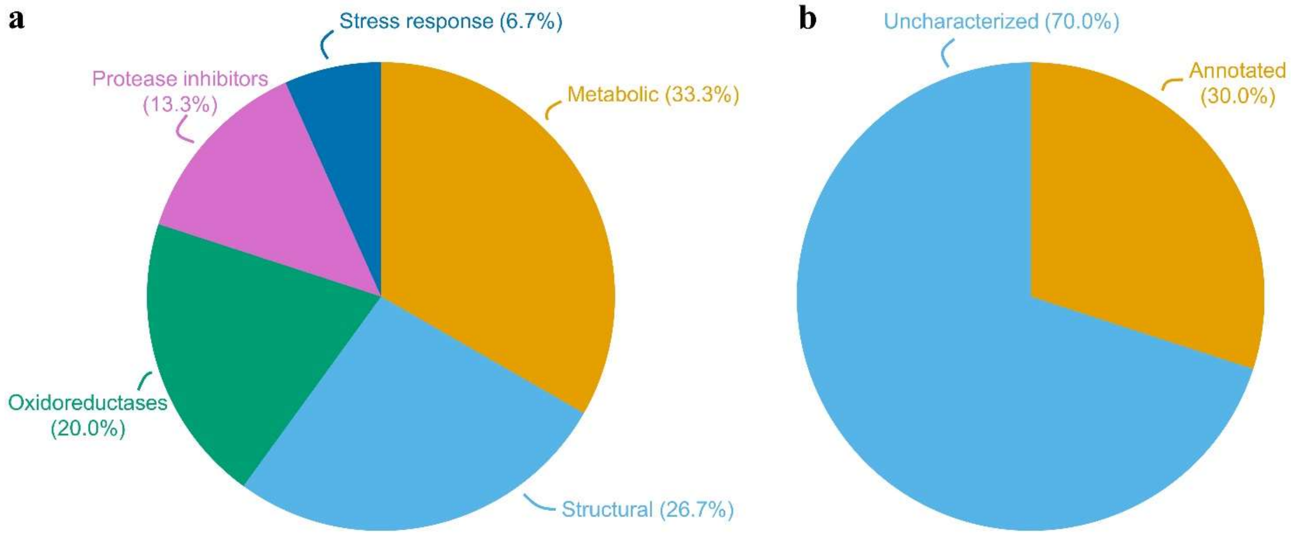

Supplementary Fig. S1. Representation of the number of proteins obtained from the foam produced by *Mahanarva spectabilis* nymphs infesting four forage cultivars (*Urochloa brizantha* cv. Marandu, *Urochloa decumbens* cv. Basilisk, and the elephant grass cultivars Pioneiro and Roxo de Botucatu). (a) Functional distribution of the annotated proteins into five major functional categories based on domain-level features and predicted biological roles: metabolic enzymes (33.3%), structural proteins (26.7%), oxidoreductases (20.0%), protease inhibitors (13.3%) and proteins associated with stress responses (6.7%). (b) Percentage distribution of annotated and uncharacterized proteins. The majority of identified proteins lacked full functional annotation (70%), whereas 30% presented identifiable domains or functional signatures. These annotated proteins correspond to predicted structural, metabolic, redox-related, or regulatory components inferred from domain-level information.

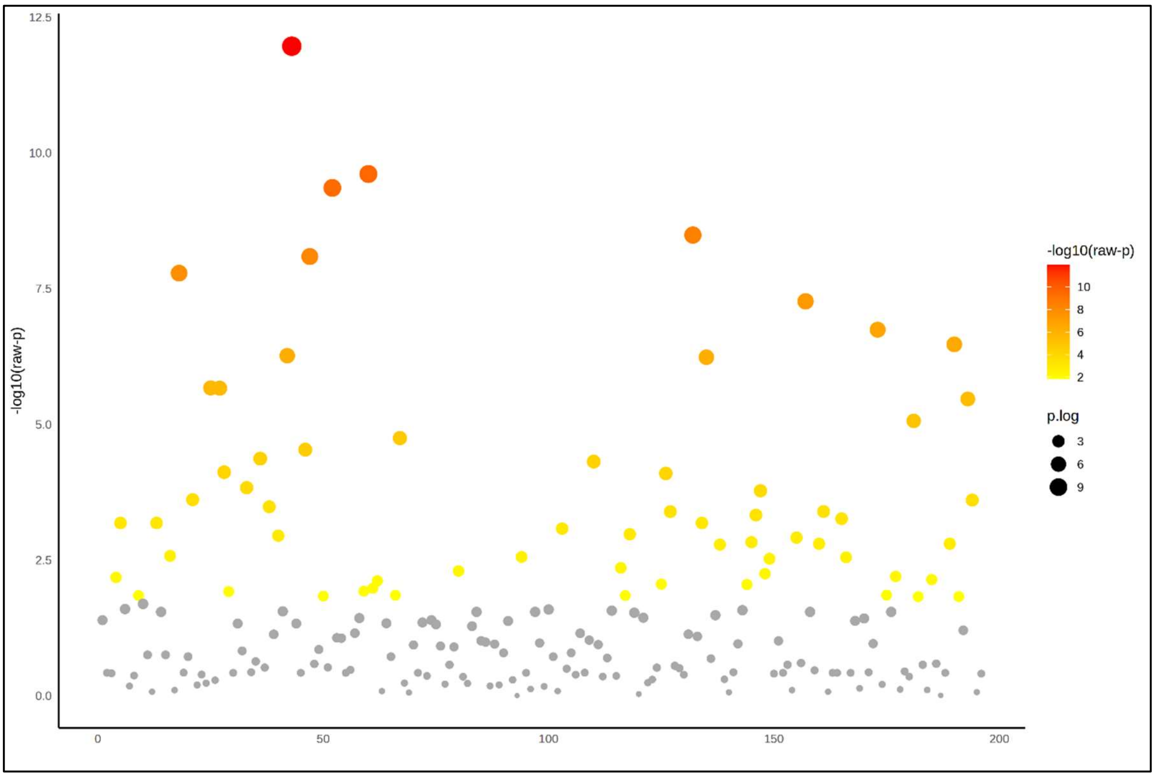

Supplementary Fig. S2. One-way ANOVA of the multigroup comparison of the identified proteins from foam produced by *Mahanarva spectabilis* nymphs infesting *Urochloa brizantha* cv. Marandu, *Urochloa decumbens* cv Basilisk, and the elephant grass (*Cenchrus purpureus*) cultivar Pioneiro and cultivar Roxo de Botucatu.

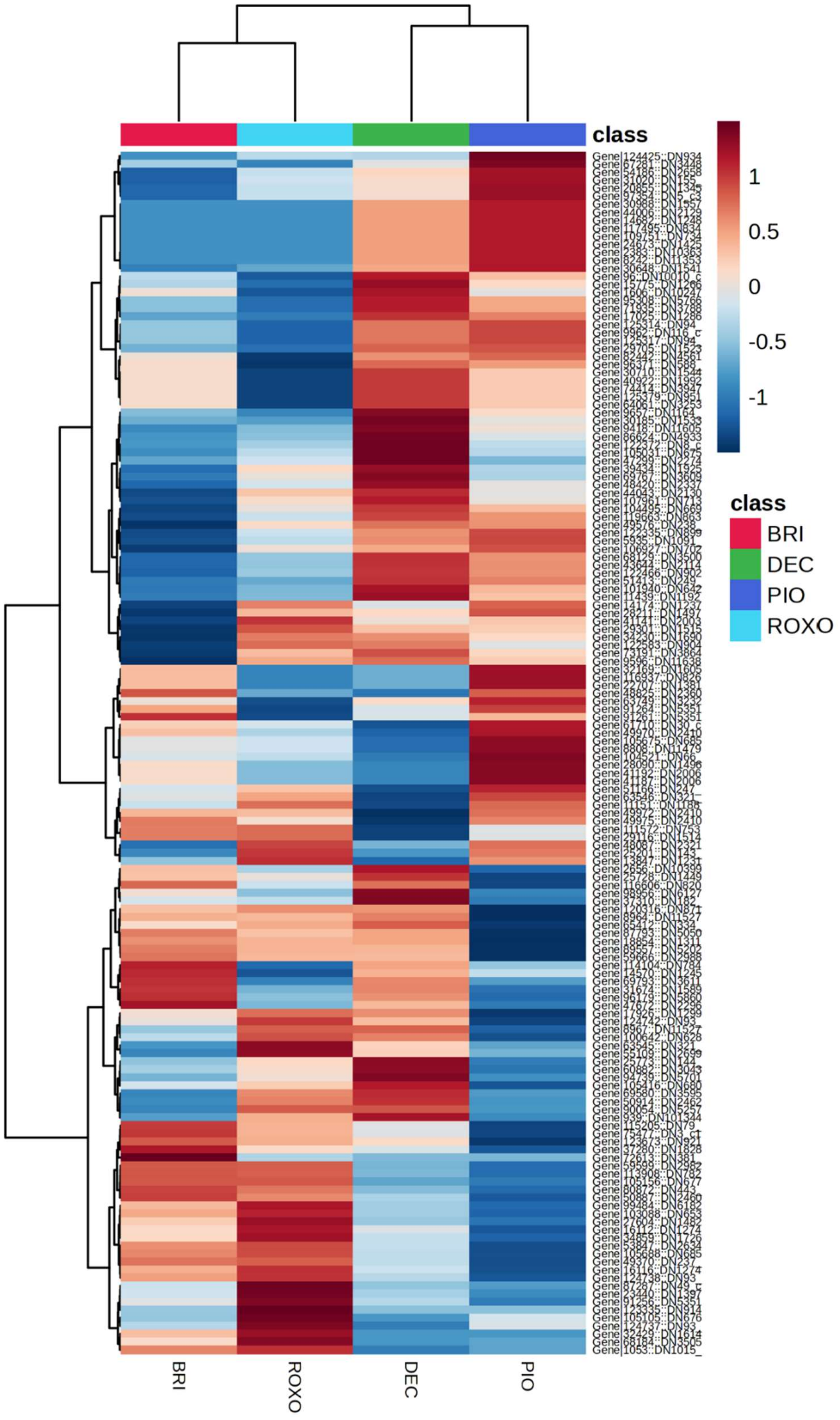

Supplementary Fig. S3. Hierarchical clustering by heatmap using top 150 identified proteins from foam produced by *Mahanarva spectabilis* nymphs infesting *Urochloa brizantha* cv. Marandu (BRI), *Urochloa decumbens* cv Basilisk (DEC), and the elephant grass (*Cenchrus purpureus*) cultivar Pioneiro (PIO) and cultivar Roxo de Botucatu (ROXO).

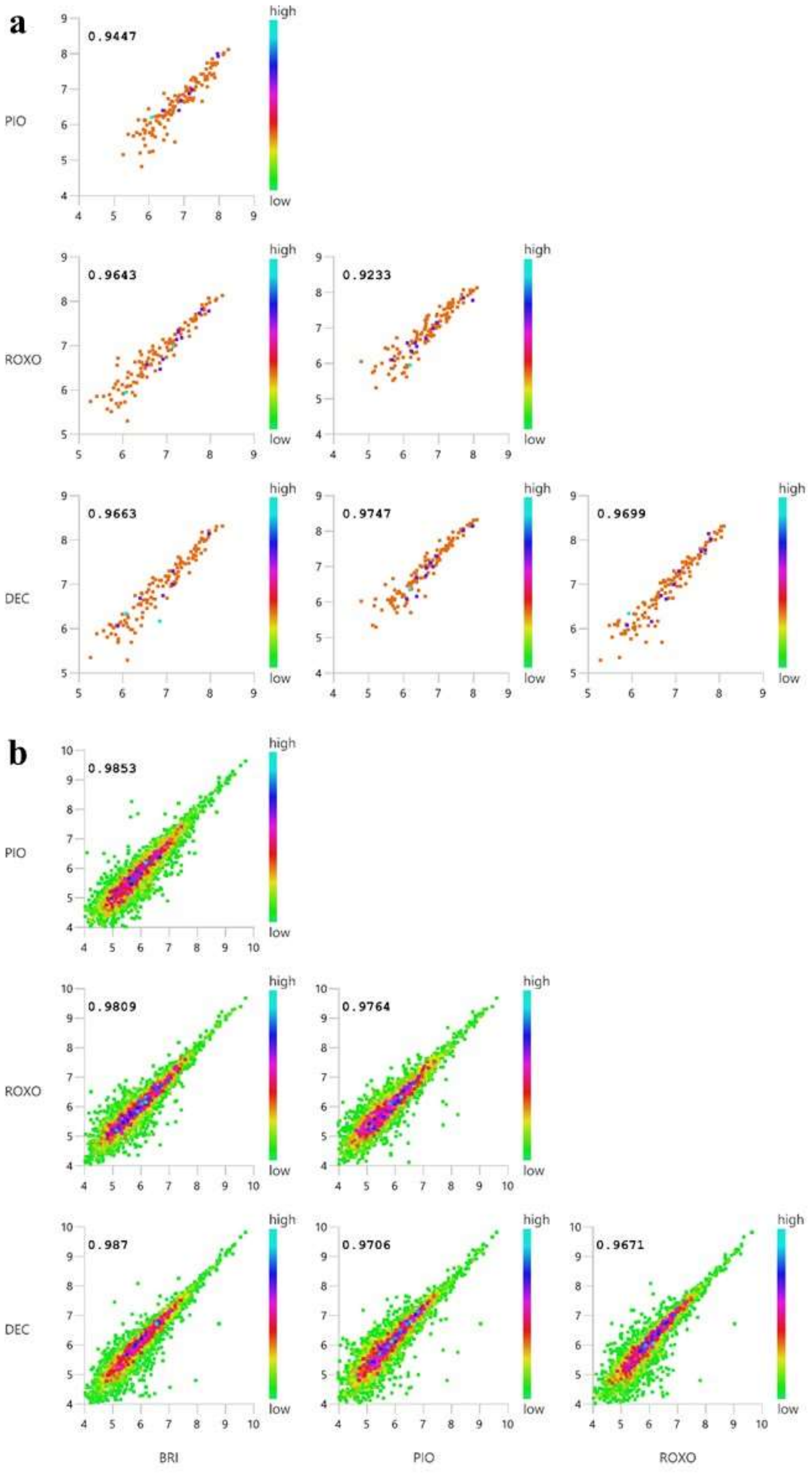

Supplementary Fig. S4. Correlation plots of protein (a) and peptide (b) identified from foam produced by *Mahanarva spectabilis* nymphs infesting *Urochloa brizantha* cv. Marandu (BRI), *Urochloa decumbens* cv Basilisk (DEC), and the elephant grass (*Cenchrus purpureus*) cultivar Pioneiro (PIO) and cultivar Roxo de Botucatu (ROXO). The Pearson's correlation score indicates the reproducibility of the experiment based on a selected pair of treatment.

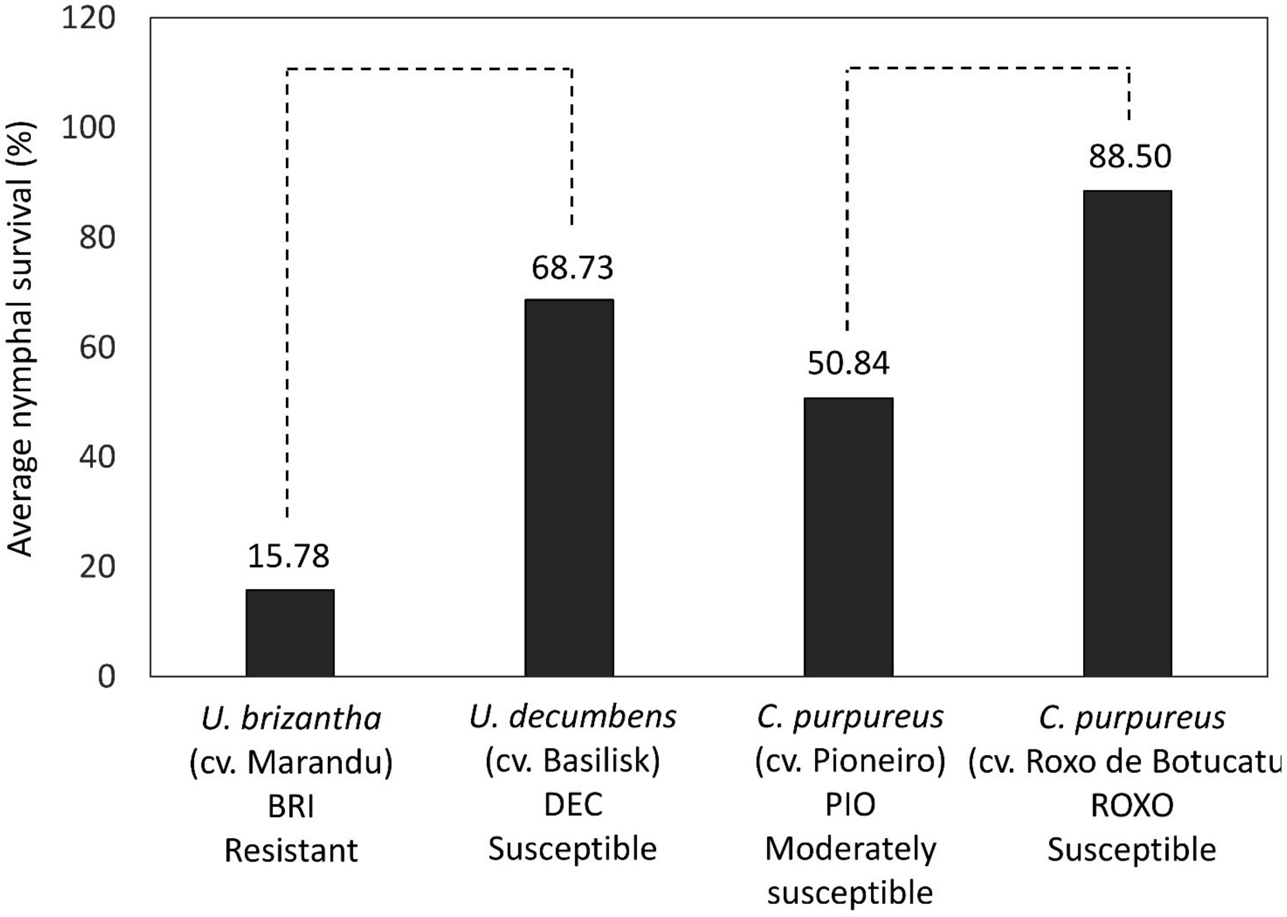

Supplementary Fig. S5. Percentage survival of spittlebug nymphs (*Mahanarva* sp.) in the four cultivars of tropical forage grasses evaluated in this study. The bars represent the average percentage of nymphal survival as retrieved from previous studies[7,8,9,10]. The higher the nymphal survival the lower the resistance level of the cultivars. The main comparisons performed here were made between the cultivars indicated by the dotted lines, i.e. between *Urochloa brizantha* cv. Marandu (BRI, resistant) and *U. decumbens* cv. Basilisk (DEC, susceptible) and between elephantgrass (*Cenchrus purpureus*) cv. Pioneiro (moderately resistant) and cv. Roxo de Botucatu (susceptible).

Supplementary Table S1. List of the 196 proteins identified by LC-MS/MS in the foam produced by *Mahanarva spectabilis* nymphs infesting *Urochloa brizantha* cv. Marandu, *Urochloa decumbens* cv Basilisk, and the elephant grass (*Cenchrus purpureus*) cultivar Pioneiro and cultivar Roxo de Botucatu. This list includes number of peptides, molecular mass and functional annotation obtained in the database GO, COG, KEGG, Pfam, SwissProt and eggNOG. Permission has been obtained from Kanehisa Laboratories for using KEGG pathway database[32,33,34].

Accession	#Peptides	#Unique	Average Mass	GO_annotation_edit	COG_class	KEGG_annotation	Pfam_annotation	Swissprot_annotation	eggNOG_class
Gene|122372::TRINITY_DN8_c0_g1::g.122372::m.122372|DN8_c0_g1:66-1559(-)	214	79	50572	Unknown	--	--	--	--	--
Gene|37310::TRINITY_DN182_c0_g1::g.37310::m.37310|DN182_c0_g1:86-4939(+)	113	107	182521	Unknown	--	--	--	--	--
Gene|105156::TRINITY_DN677_c0_g1::g.105156::m.105156|DN677_c0_g1:73-2916(+)	104	104	106270	Unknown	--	--	--	--	--
Gene|105027::TRINITY_DN675_c0_g1::g.105027::m.105027|DN675_c0_g1:96-1106(-)	103	81	34923	Unknown	--	--	--	--	--
Gene|14174::TRINITY_DN1237_c0_g1::g.14174::m.14174|DN1237_c0_g1:78-2423(-)	102	100	88435	Unknown	--	--	--	--	--
Gene|55109::TRINITY_DN2699_c0_g1::g.55109::m.55109|DN2699_c0_g1:2-11059(+)	95	91	412060	Unknown	--	--	--	--	--
Gene|105031::TRINITY_DN675_c1_g1::g.105031::m.105031|DN675_c1_g1:3-431(-)	66	40	14445	Unknown	--	--	--	--	--
Gene|49576::TRINITY_DN238_c0_g1::g.49576::m.49576|DN238_c0_g1:93-2378(-)	58	57	87976	Unknown	--	--	--	--	--
Gene|32429::TRINITY_DN1614_c0_g1::g.32429::m.32429|DN1614_c0_g1:3-347(+)	58	4	11632	Unknown	--	--	--	--	--
Gene|115205::TRINITY_DN79_c0_g1::g.115205::m.115205|DN79_c0_g1:285-5393(+)	57	57	190720	Unknown	--	--	--	--	--
Gene|82896::TRINITY_DN45_c1_g1::g.82896::m.82896|DN45_c1_g1:3-2114(+)	55	47	79066	Unknown	--	--	--	--	--
Gene|60882::TRINITY_DN3043_c0_g1::g.60882::m.60882|DN3043_c0_g1:111-4721(-)	53	52	174606	Unknown	--	--	--	--	--
Gene|114104::TRINITY_DN784_c0_g1::g.114104::m.114104|DN784_c0_g1:79-2442(-)	53	51	84049	Unknown	--	--	--	--	--
Gene|86182::TRINITY_DN4893_c0_g1::g.86182::m.86182|DN4893_c0_g1:230-1672(-)	52	51	54168	Unknown	--	--	--	--	--
Gene|48420::TRINITY_DN2337_c1_g1::g.48420::m.48420|DN2337_c1_g1:2206-4263(+)	48	46	77035	Unknown	--	--	--	--	--
Gene|28910::TRINITY_DN1508_c0_g1::g.28910::m.28910|DN1508_c0_g1:102-1517(+)	48	42	52136	Unknown	--	--	--	--	--
Gene|63546::TRINITY_DN321_c1_g1::g.63546::m.63546|DN321_c1_g1:75-803(-)	47	28	24902	Unknown	--	--	--	--	--
Gene|52303::TRINITY_DN254_c0_g1::g.52303::m.52303|DN254_c0_g1:1012-4950(-)	45	45	145860	Unknown	--	--	--	--	--
Gene|103375::TRINITY_DN656_c0_g1::g.103375::m.103375|DN656_c0_g1:1-426(+)	42	8	14598	Unknown	--	--	--	--	--
Gene|1606::TRINITY_DN102479_c0_g1::g.1606::m.1606|DN102479_c0_g1:1355-4060(-)	41	41	102901	Unknown	--	--	--	--	--
Gene|107961::TRINITY_DN713_c0_g1::g.107961::m.107961|DN713_c0_g1:185-1354(-)	41	41	43221	Protease Inhibitor	[O]	K13963|8.9e-64|mde:101895742|K13963 serpin B | (RefSeq) serine protease inhibitor 42Dd isoform X1	Serpin (serine protease inhibitor)	Antitrypsin OS=Bombyx mori OX=7091 PE=1 SV=2	V
Gene|91256::TRINITY_DN5351_c0_g1::g.91256::m.91256|DN5351_c0_g1:1-2286(+)	41	40	78382	Unknown	--	--	--	--	--
Gene|41141::TRINITY_DN2003_c0_g1::g.41141::m.41141|DN2003_c0_g1:2070-3482(-)	39	39	51195	Unknown	--	--	--	--	--
Gene|25201::TRINITY_DN143_c1_g1::g.25201::m.25201|DN143_c1_g1:2-358(+)	37	21	11712	Unknown	--	--	--	--	--
Gene|29705::TRINITY_DN1523_c0_g1::g.29705::m.29705|DN1523_c0_g1:3-329(-)	35	8	11610	Unknown	--	--	--	--	--

Gene|103377::TRINITY_DN656_c0_g2::g.103377::m.103377|DN656_c0_g2:1-363(+)	35	8	12404	Unknown	--	--	--	--	--
Gene|31674::TRINITY_DN15891_c0_g2::g.31674::m.31674|DN15891_c0_g2:45-8231(-)	34	33	312116	Unknown	--	--	--	--	--
Gene|51413::TRINITY_DN249_c0_g1::g.51413::m.51413|DN249_c0_g1:109-3054(-)	33	33	109915	Unknown	--	--	--	--	--
Gene|65412::TRINITY_DN334_c1_g1::g.65412::m.65412|DN334_c1_g1:1-282(-)	33	6	9948	Unknown	--	--	--	--	--
Gene|25773::TRINITY_DN144_c0_g1::g.25773::m.25773|DN144_c0_g1:220-1704(-)	32	30	53803	Unknown	--	--	--	--	--
Gene|65410::TRINITY_DN334_c0_g1::g.65410::m.65410|DN334_c0_g1:2-232(-)	32	9	7927	Unknown	--	--	--	--	--
Gene|100644::TRINITY_DN628_c2_g1::g.100644::m.100644|DN628_c2_g1:2-283(-)	32	8	9689	Unknown	--	--	--	--	--
Gene|31020::TRINITY_DN155_c0_g1::g.31020::m.31020|DN155_c0_g1:69-344(-)	29	11	9307	Unknown	--	--	--	--	--
Gene|13847::TRINITY_DN1231_c0_g1::g.13847::m.13847|DN1231_c0_g1:130-1839(-)	26	26	63876	Hydrolase Activity - Lipid Metabolism	[I]	K12298|4.7e-123|hst:105189116|K12298 bile salt-stimulated lipase [EC:3.1.1.3 3.1.1.13] | (RefSeq) venom carboxylesterase-6-like	Carboxylesterase family;; alpha/beta hydrolase fold	Esterase FE4 OS=Myzus persicae OX=13164 PE=1 SV=1	G
Gene|124738::TRINITY_DN93_c0_g2::g.124738::m.124738|DN93_c0_g2:118-1986(-)	26	24	67986	Chitin Binding	--	K03900|3.5e-23|dan:6506118|K03900 von Willebrand factor | (RefSeq) hemocytin	WSC domain;; EGF-like domain	--	--
Gene|127572::TRINITY_DN98623_c0_g1::g.127572::m.127572|DN98623_c0_g1:1-1323(+)	25	25	47676	Protease Inhibitor	--	K23624|4.7e-84|dnx:107166649|K23624 papilin | (RefSeq) papilin-like isoform X1	Kunitz/Bovine pancreatic trypsin inhibitor domain	Papilin OS=Drosophila melanogaster OX=7227 GN=Ppn PE=1 SV=2	--
Gene|9596::TRINITY_DN116380_c0_g1::g.9596::m.9596|DN116380_c0_g1:2-3931(+)	25	25	144771	Unknown	--	--	--	--	--
Gene|8964::TRINITY_DN115271_c1_g1::g.8964::m.8964|DN115271_c1_g1:9-455(+)	25	20	16711	Unknown	--	--	--	--	--
Gene|73191::TRINITY_DN38649_c0_g1::g.73191::m.73191|DN38649_c0_g1:698-2857(-)	24	23	80616	Unknown	--	--	--	--	--
Gene|80872::TRINITY_DN443_c4_g1::g.80872::m.80872|DN443_c4_g1:133-1854(-)	24	21	62263	Chitin Binding	--	K17545|7.6e-36|crg:105343229|K17545 serine/threonine-protein kinase ULK4 [EC:2.7.11.1] | (RefSeq) serine/threonine-protein kinase ULK4-like	WSC domain;; EGF-like domain;; Glycoside hydrolase family 5 C-terminal domain	Xylosyltransferase sqv-6 OS=Caenorhabditis briggsae OX=6238 GN=sqv-6 PE=2 SV=1	--
Gene|91261::TRINITY_DN5351_c1_g1::g.91261::m.91261|DN5351_c1_g1:1-1473(-)	23	23	50742	Unknown	--	--	Protein of unknown function (DUF725)	--	--
Gene|16112::TRINITY_DN1274_c0_g1::g.16112::m.16112|DN1274_c0_g1:1-1236(+)	23	17	45553	Unknown	--	K00771|2.4e-14|api:100163472|K00771 protein xylosyltransferase [EC:2.4.2.26] | (RefSeq) xylosyltransferase oxt	WSC domain	--	--
Gene|27604::TRINITY_DN148298_c1_g1::g.27604::m.27604|DN148298_c1_g1:168-1202(-)	23	17	38963	Hydrolase Activity - Lipid Metabolism	--	K01771|3.1e-50|fcd:110844986|K01771 1-phosphatidylinositol phosphodiesterase [EC:4.6.1.13] | (RefSeq) uncharacterized protein LOC110844986	--	--	S
Gene|103088::TRINITY_DN6538_c0_g1::g.103088::m.103088|DN6538_c0_g1:68-2533(-)	22	22	90929	Protease Inhibidor	[R]	K19014|6.4e-89|oaa:100091835|K19014 inter-alpha-trypsin inhibitor heavy chain H1 | (RefSeq) inter-alpha-trypsin inhibitor heavy chain H3-like isoform X1	Vault protein inter-alpha-trypsin domain;; von Willebrand factor type A domain;; von Willebrand factor type A domain	--	S
Gene|124742::TRINITY_DN93_c1_g1::g.124742::m.124742|DN93_c1_g1:3-1805(-)	22	17	65901	Hydrolase Activity - Carbohydrate Metabolism	--	K00771|3.6e-14|aag:5569146|K00771 protein xylosyltransferase [EC:2.4.2.26] | (RefSeq) xylosyltransferase oxt	WSC domain;; Glycoside hydrolase family 5 C-terminal domain	--	--
Gene|11151::TRINITY_DN118823_c0_g1::g.11151::m.11151|DN118823_c0_g1:3-311(+)	22	8	10374	Unknown	--	--	--	--	--
Gene|49975::TRINITY_DN24104_c2_g1::g.49975::m.49975|DN24104_c2_g1:139-1269(+)	22	3	41785	ATP Binding – Actin	[Z]	K05692|4.6e-217|dpx:DAPPUDRAFT_228751|K05692 actin beta/gamma 1 | (RefSeq) ACT1B; cytoplasmatic actin	Actin	Actin, muscle OS=Manduca sexta OX=7130 PE=2 SV=1	Z
Gene|61710::TRINITY_DN30_c0_g1::g.61710::m.61710|DN30_c0_g1:353-1483(+)	21	2	41821	ATP Binding – Actin	[Z]	K05692|2.4e-218|aag:5574526|K05692 actin beta/gamma 1 | (RefSeq) actin-5C	Actin	Actin-5C OS=Drosophila melanogaster OX=7227 GN=Act5C PE=1 SV=4	Z
Gene|106927::TRINITY_DN702_c0_g1::g.106927::m.106927|DN702_c0_g1:258-1313(+)	20	15	39611	Hydrolase Activity - Lipid Metabolism	[U]	K01771|2.0e-69|fcd:110844986|K01771 1-phosphatidylinositol phosphodiesterase	Phosphatidylinositol-specific phospholipase C, X domain	--	T

						[EC:4.6.1.13] | (RefSeq) uncharacterized protein LOC110844986			
Gene|51166::TRINITY_DN247_c0_g1::g.51166::m.51166|DN247_c0_g1:3-287(+)	20	7	9529	Unknown	--	--	--	--	--
Gene|86624::TRINITY_DN49332_c0_g1::g.86624::m.86624|DN49332_c0_g1:195-2168(-)	19	19	74657	Oxidoreductase Activity - Copper Ion Binding	[DPM]	K00423|3.7e-119|dpx:DAPPUDRAFT_317026|K00423 L-ascorbate oxidase [EC:1.10.3.3] | (RefSeq) hypothetical protein	Multicopper oxidase;; Multicopper oxidase;; Multicopper oxidase	--	Q
Gene|105416::TRINITY_DN680_c0_g1::g.105416::m.105416|DN680_c0_g1:115-1419(-)	19	19	49192	Unknown	--	--	--	--	--
Gene|105105::TRINITY_DN676_c0_g1::g.105105::m.105105|DN676_c0_g1:113-754(-)	18	18	24534	Unknown	--	--	--	--	--
Gene|87287::TRINITY_DN49_c0_g1::g.87287::m.87287|DN49_c0_g1:303-857(-)	18	18	19498	Superoxide Dismutase Activity - Metal Ion Binding	[P]	K04565|6.7e-33|dhe:111604418|K04565 superoxide dismutase, Cu-Zn family [EC:1.15.1.1] | (RefSeq) superoxide dismutase [Cu-Zn]	Copper/zinc superoxide dismutase (SODC)	Superoxide dismutase [Cu-Zn] OS=Drosophila virilis OX=7244 GN=Sod1 PE=3 SV=2	Q
Gene|69767::TRINITY_DN36093_c0_g1::g.69767::m.69767|DN36093_c0_g1:2-2098(+)	17	17	76478	Unknown	--	--	--	--	--
Gene|102840::TRINITY_DN650_c0_g1::g.102840::m.102840|DN650_c0_g1:67-741(-)	17	16	23807	Unknown	--	--	--	--	--
Gene|116606::TRINITY_DN8201_c0_g1::g.116606::m.116606|DN8201_c0_g1:56-1201(-)	17	15	43296	Hydrolase Activity - Lipid Metabolism	[I]	K03927|5.7e-72|zne:110841418|K03927 carboxylesterase 2 [EC:3.1.1.1 3.1.1.84 3.1.1.56] | (RefSeq) LOW QUALITY PROTEIN: uncharacterized protein LOC110841418	Carboxylesterase family;; alpha/beta hydrolase fold;; Carboxylesterase family	Esterase E4 OS=Myzus persicae OX=13164 PE=1 SV=1	G
Gene|120316::TRINITY_DN8712_c0_g1::g.120316::m.120316|DN8712_c0_g1:2-1453(+)	16	16	54284	Unknown	--	--	--	--	--
Gene|48087::TRINITY_DN2321_c0_g1::g.48087::m.48087|DN2321_c0_g1:3-1766(+)	16	16	64416	Unknown	--	--	--	--	--
Gene|100642::TRINITY_DN628_c0_g1::g.100642::m.100642|DN628_c0_g1:2-310(-)	16	9	10435	Unknown	--	--	--	--	--
Gene|44023::TRINITY_DN21296_c0_g3::g.44023::m.44023|DN21296_c0_g3:218-823(-)	15	15	23269	Lipocalin - Fatty-acid Binding	--	--	Lipocalin / cytosolic fatty-acid binding protein family;; Triabin	--	--
Gene|44019::TRINITY_DN21296_c0_g1::g.44019::m.44019|DN21296_c0_g1:119-724(+)	15	15	23269	Lipocalin - Fatty-acid Binding	--	--	Lipocalin / cytosolic fatty-acid binding protein family;; Triabin	--	--
Gene|91264::TRINITY_DN5351_c1_g2::g.91264::m.91264|DN5351_c1_g2:3-857(+)	15	15	30245	Unknown	--	--	--	--	--
Gene|15775::TRINITY_DN1266_c0_g1::g.15775::m.15775|DN1266_c0_g1:45-1472(+)	15	15	51516	Unknown	--	--	--	--	--
Gene|9657::TRINITY_DN1164_c0_g1::g.9657::m.9657|DN1164_c0_g1:72-1391(+)	15	15	51297	Unknown	--	--	--	--	--
Gene|119663::TRINITY_DN86362_c0_g1::g.119663::m.119663|DN86362_c0_g1:95-1357(+)	15	15	48076	Melanin Metabolic Process	[G]	K22203|5.4e-61|mde:101889119|K22203 dopachrome tautomerase [EC:5.3.3.12] | (RefSeq) L-dopachrome tautomerase yellow-f2	Major royal jelly protein	Major royal jelly protein 1 OS=Apis mellifera OX=7460 GN=MRJP1 PE=1 SV=1	G
Gene|23440::TRINITY_DN13974_c0_g1::g.23440::m.23440|DN13974_c0_g1:3-1604(-)	15	14	58891	Hydrolase Activity - Carbohydrate Metabolism	--	K00771|1.4e-14|tca:657929|K00771 protein xylosyltransferase [EC:2.4.2.26] | (RefSeq) xylosyltransferase oxt	WSC domain;; EGF-like domain	--	U
Gene|44043::TRINITY_DN21301_c0_g1::g.44043::m.44043|DN21301_c0_g1:17-1510(-)	14	14	56874	Hydrolase Activity - Carbohydrate Metabolism	[G]	K01229|4.8e-125|tgu:100229941|K01229 lactase-phlorizin hydrolase [EC:3.2.1.108 3.2.1.62] | (RefSeq) LCT; lactase-phlorizin hydrolase	Glycosyl hydrolase family 1	Myrosinase 1 OS=Brevicoryne brassicae OX=69196 PE=1 SV=1	G
Gene|72613::TRINITY_DN381_c1_g1::g.72613::m.72613|DN381_c1_g1:1-864(+)	14	13	32127	Xylosyltransferase - Gram-negative Bacteria Binding	--	K00771|4.2e-21|lak:106159130|K00771 protein xylosyltransferase [EC:2.4.2.26] | (RefSeq) xylosyltransferase oxt	WSC domain	Xylosyltransferase sqv-6 OS=Caenorhabditis briggsae OX=6238 GN=sqv-6 PE=2 SV=1	S
Gene|17025::TRINITY_DN128683_c0_g2::g.17025::m.17025|DN128683_c0_g2:518-2014(-)	13	13	51101	Unknown	--	--	--	--	--
Gene|105688::TRINITY_DN6854_c0_g1::g.105688::m.105688|DN6854_c0_g1:124-1113(-)	13	12	35871	Hydrolase Activity - Lipid Metabolism	--	K19404|6.3e-38|lhu:105678253|K19404 phosphatidic acid-selective phospholipase A1 [EC:3.1.1.-] | (RefSeq) pancreatic triacylglycerol lipase-like	Lipase	Phospholipase A1 OS=Vespa velutina OX=202808 PE=1 SV=1	O

Gene|39089::TRINITY_DN19110_c0_g2::g.39089::m.39089|DN19110_c0_g2:3-1280(+)	13	11	47596	Unknown	--	--	--	--	--
Gene|18854::TRINITY_DN1311_c0_g1::g.18854::m.18854|DN1311_c0_g1:1-330(+)	13	8	12104	Unknown	--	--	--	--	--
Gene|8967::TRINITY_DN115271_c2_g1::g.8967::m.8967|DN115271_c2_g1:2-295(+)	12	12	11366	Unknown	--	--	--	--	--
Gene|68132::TRINITY_DN35000_c0_g2::g.68132::m.68132|DN35000_c0_g2:34-1029(+)	12	12	35938	Unknown	--	--	--	--	--
Gene|123673::TRINITY_DN92162_c1_g1::g.123673::m.123673|DN92162_c1_g1:133-1203(-)	11	11	40461	Hydrolase Activity - Lipid Metabolism	--	K01771|1.7e-59|fcd:110844986|K01771 1-phosphatidylinositol phosphodiesterase [EC:4.6.1.13] | (RefSeq) uncharacterized protein LOC110844986	--	--	T
Gene|104495::TRINITY_DN6696_c1_g1::g.104495::m.104495|DN6696_c1_g1:54-710(+)	11	11	24046	Unknown	--	--	--	--	--
Gene|49370::TRINITY_DN237_c0_g1::g.49370::m.49370|DN237_c0_g1:1-1101(-)	11	11	39597	Unknown	--	--	--	--	--
Gene|122466::TRINITY_DN90258_c0_g1::g.122466::m.122466|DN90258_c0_g1:50-1468(+)	11	10	53685	Unknown	--	--	--	--	--
Gene|63545::TRINITY_DN321_c0_g1::g.63545::m.63545|DN321_c0_g1:2-217(+)	11	7	7152	Unknown	--	--	--	--	--
Gene|19684::TRINITY_DN132717_c0_g1::g.19684::m.19684|DN132717_c0_g1:156-602(-)	11	5	16887	Unknown	--	--	--	--	--
Gene|49972::TRINITY_DN24104_c1_g2::g.49972::m.49972|DN24104_c1_g2:1-699(-)	11	1	26154	ATP Binding – Actin	[Z]	K05692|1.4e-128|dpx:DAPPUDRAFT_228751|K05692 actin beta/gamma 1 | (RefSeq) ACT1B; cytoplasmatic actin	Actin	Actin, muscle-type A1 OS=Bombyx mori OX=7091 PE=3 SV=1	Z
Gene|5935::TRINITY_DN1091_c0_g1::g.5935::m.5935|DN1091_c0_g1:2-277(+)	10	10	9301	Unknown	--	--	--	--	--
Gene|43644::TRINITY_DN21145_c0_g1::g.43644::m.43644|DN21145_c0_g1:171-1568(+)	10	10	53195	Hydrolase Activity - Protease	[E]	K09645|6.1e-145|zne:110838721|K09645 vitellogenic carboxypeptidase-like protein [EC:3.4.16.-] | (RefSeq) venom serine carboxypeptidase	Serine carboxypeptidase	Venom serine carboxypeptidase OS=Apis mellifera OX=7460 PE=2 SV=1	O
Gene|37280::TRINITY_DN1828_c0_g1::g.37280::m.37280|DN1828_c0_g1:103-1872(+)	10	10	64305	Signal Transduction - Protease Inhibitor	--	--	--	--	T
Gene|17926::TRINITY_DN1299_c0_g1::g.17926::m.17926|DN1299_c0_g1:1726-3573(-)	10	10	68742	Posttranslational modification - protein turnover - chaperones	[O]	K04525|1.1e-88|clec:106669534|K04525 serpin A | (RefSeq) uncharacterized protein LOC106669534 isoform X1	Serpin (serine protease inhibitor)	Serine protease inhibitor 27A OS=Drosophila melanogaster OX=7227 GN=Spn27A PE=1 SV=1	V
Gene|9418::TRINITY_DN11605_c0_g1::g.9418::m.9418|DN11605_c0_g1:119-754(+)	10	10	22422	Apolipoprotein	--	--	--	--	--
Gene|96::TRINITY_DN10010_c0_g1::g.96::m.96|DN10010_c0_g1:103-1857(-)	10	10	65918	Hydrolase Activity - Lipid Metabolism	[I]	K03927|1.5e-92|tca:655171|K03927 carboxylesterase 2 [EC:3.1.1.1 3.1.1.84 3.1.1.56] | (RefSeq) uncharacterized LOC655171	Carboxylesterase family;; alpha/beta hydrolase fold	Esterase FE4 OS=Myzus persicae OX=13164 PE=1 SV=1	G
Gene|123335::TRINITY_DN9149_c2_g1::g.123335::m.123335|DN9149_c2_g1:348-920(-)	10	9	20859	Chitin Binding	--	K00771|1.8e-14|aag:5569146|K00771 protein xylosyltransferase [EC:2.4.2.26] | (RefSeq) xylosyltransferase oxt	WSC domain	--	--
Gene|14570::TRINITY_DN124584_c0_g1::g.14570::m.14570|DN124584_c0_g1:85-1530(-)	9	9	52312	Posttranslational Modification - Protein Turnover – Chaperones	--	--	--	--	O
Gene|86762::TRINITY_DN4946_c1_g1::g.86762::m.86762|DN4946_c1_g1:142-996(+)	9	9	31111	Hydrolase Activity - Protease	--	K01312|5.5e-21|bim:100747896|K01312 trypsin [EC:3.4.21.4] | (RefSeq) trypsin-4-like	Trypsin	Trypsin theta OS=Drosophila melanogaster OX=7227 GN=thetaTry PE=2 SV=2	O
Gene|75477::TRINITY_DN3_c1_g1::g.75477::m.75477|DN3_c1_g1:100-525(-)	9	9	16581	Unknown	--	--	--	--	--
Gene|25728::TRINITY_DN144938_c0_g1::g.25728::m.25728|DN144938_c0_g1:2-286(+)	9	9	10388	Protein Binding - Ubiquitin Family	--	--	Ubiquitin family	--	--
Gene|98956::TRINITY_DN61276_c0_g2::g.98956::m.98956|DN61276_c0_g2:25-1965(+)	9	9	71473	Oxidoreductase Activity	[IR]	K00108|8.9e-102|ame:552425|K00108 choline dehydrogenase [EC:1.1.99.1] | (RefSeq) glucose dehydrogenase [FAD, quinone]	GMC oxidoreductase;; GMC oxidoreductase	Glucose dehydrogenase [FAD, quinone] OS=Drosophila melanogaster OX=7227 GN=Gld PE=3 SV=3	E

Gene|939::TRINITY_DN101344_c0_g1::g.939::m.939|DN101344_c0_g1:3-548(-)	9	3	20881	Hydrolase activity - Lipid Metabolism	--	K01771|4.5e-21|fcd:110844986|K01771 1-phosphatidylinositol phosphodiesterase [EC:4.6.1.13] | (RefSeq) uncharacterized protein LOC110844986	--	--	S
Gene|30185::TRINITY_DN153313_c0_g1::g.30185::m.30185|DN153313_c0_g1:173-778(-)	8	8	23338	Unknown	--	--	--	--	--
Gene|63746::TRINITY_DN32320_c0_g1::g.63746::m.63746|DN32320_c0_g1:49-516(-)	8	8	17385	Unknown	--	--	--	--	--
Gene|28211::TRINITY_DN149759_c0_g1::g.28211::m.28211|DN149759_c0_g1:56-760(-)	8	8	26555	Unknown	--	--	--	--	--
Gene|34607::TRINITY_DN1712_c2_g1::g.34607::m.34607|DN1712_c2_g1:113-424(+)	8	8	11381	DNA Binding - Histone	--	K11254|1.3e-50|aag:110678205|K11254 histone H4 | (RefSeq) histone H4-like	Centromere kinetochore component CENP-T histone fold;; TATA box binding protein associated factor (TAF)	Histone H4 OS=Acrolepiopsis assectella OX=57686 GN=His4 PE=3 SV=2	B
Gene|87793::TRINITY_DN5050_c0_g2::g.87793::m.87793|DN5050_c0_g2:2-1132(+)	8	6	41880	Hydrolase Activity – Glycosidase	--	K00771|1.6e-07|ame:413854|K00771 protein xylosyltransferase [EC:2.4.2.26] | (RefSeq) xylosyltransferase oxt	WSC domain	--	S
Gene|90054::TRINITY_DN5257_c0_g1::g.90054::m.90054|DN5257_c0_g1:1-2115(+)	8	5	76651	Hydrolase Activity - Carbohydrate Metabolism	--	K00771|6.0e-13|cqu:CpipJ_CPIJ019812|K00771 protein xylosyltransferase [EC:2.4.2.26] | (RefSeq) xylosyltransferase oxt	WSC domain;; EGF-like domain	--	--
Gene|9962::TRINITY_DN116_c0_g1::g.9962::m.9962|DN116_c0_g1:695-2047(-)	7	7	49978	GTPase Activity – Tubulin	--	K07374|1.6e-266|ame:408388|K07374 tubulin alpha | (RefSeq) tubulin alpha-1 chain	Tubulin/FtsZ family, GTPase domain;; Tubulin C-terminal domain	Tubulin alpha-1 chain OS=Drosophila melanogaster OX=7227 GN=alphaTub84B PE=1 SV=1	Z
Gene|125314::TRINITY_DN94_c0_g1::g.125314::m.125314|DN94_c0_g1:125-1477(+)	7	7	50026	GTPase activity – Tubulin	--	K07374|5.7e-266|phu:Phum_PHUM420710|K07374 tubulin alpha | (RefSeq) tubulin alpha-1 chain	Tubulin/FtsZ family, GTPase domain;; Tubulin C-terminal domain	Tubulin alpha-1 chain OS=Drosophila melanogaster OX=7227 GN=alphaTub84B PE=1 SV=1	Z
Gene|24312::TRINITY_DN14154_c0_g1::g.24312::m.24312|DN14154_c0_g1:55-1125(-)	7	7	40879	Hyalurononglucosaminidase Activity - Carbohydrate Metabolism	--	K01197|3.0e-51|bter:100651053|K01197 hyaluronoglucosaminidase [EC:3.2.1.35] | (RefSeq) hyaluronidase	Hyaluronidase	Hyaluronidase OS=Apis mellifera OX=7460 PE=1 SV=1	S
Gene|50887::TRINITY_DN2460_c0_g1::g.50887::m.50887|DN2460_c0_g1:168-752(-)	7	7	21239	Protease Inhibidor	--	K23624|6.6e-35|dgr:Dgri_GH18720|K23624 papilin | (RefSeq) GH18720 gene product from transcript GH18720-RA	Kunitz/Bovine pancreatic trypsin inhibitor domain	Papilin OS=Drosophila melanogaster OX=7227 GN=Ppn PE=1 SV=2	O
Gene|57421::TRINITY_DN28334_c0_g1::g.57421::m.57421|DN28334_c0_g1:126-743(+)	7	7	23295	Mitigation of Host Defenses by Symbiont	--	--	--	--	--
Gene|50914::TRINITY_DN24623_c0_g1::g.50914::m.50914|DN24623_c0_g1:147-1601(-)	7	7	53802	Unknown	--	--	--	--	--
Gene|125317::TRINITY_DN94_c1_g1::g.125317::m.125317|DN94_c1_g1:1-1149(-)	7	7	42367	GTPase Activity – Tubulin	--	K07374|1.4e-225|api:100166930|K07374 tubulin alpha | (RefSeq) tubulin alpha-1C chain	Tubulin/FtsZ family, GTPase domain;; Tubulin C-terminal domain	Tubulin alpha-1 chain OS=Drosophila melanogaster OX=7227 GN=alphaTub84B PE=1 SV=1	Z
Gene|99484::TRINITY_DN6182_c1_g1::g.99484::m.99484|DN6182_c1_g1:1030-2628(-)	7	7	58478	Unknown	--	--	--	--	--
Gene|29301::TRINITY_DN151595_c0_g1::g.29301::m.29301|DN151595_c0_g1:3-1367(+)	7	7	50215	Unknown	--	--	--	--	--
Gene|26346::TRINITY_DN146260_c12_g1::g.26346::m.26346|DN146260_c12_g1:53-496(-)	6	6	16792	DNA Binding - Histone	--	K11253|1.4e-66|cqu:CpipJ_CPIJ012420|K11253 histone H3 | (RefSeq) Histone H3c	Core histone H2A/H2B/H3/H4	Histone H3 OS=Chironomus thummi OX=7155 PE=3 SV=2	B
Gene|96179::TRINITY_DN58605_c0_g1::g.96179::m.96179|DN58605_c0_g1:3-476(+)	6	6	17143	Instar Larval or Pupal Development - Lipid Metabolism	--	K13443|3.3e-28|haw:110373467|K13443 Niemann-Pick C2 protein | (RefSeq) ecdysteroid-regulated 16 kDa protein-like	ML domain	--	S
Gene|104521::TRINITY_DN66_c0_g1::g.104521::m.104521|DN66_c0_g1:101-1489(+)	6	6	50625	Structural - Ribosome	[J]	K03231|2.8e-259|zne:110832006|K03231 elongation factor 1-alpha | (RefSeq) elongation factor 1-alpha	Elongation factor Tu GTP binding domain;; Elongation factor Tu C-terminal domain;; Elongation factor Tu domain 2	Elongation factor 1-alpha 2 OS=Drosophila melanogaster OX=7227 GN=eEF1alpha2 PE=2 SV=2	J

Gene|34859::TRINITY_DN17269_c0_g1::g.34859::m.34859|DN17269_c0_g1:1-594(+)	6	6	22563	Unknown	--	--	--	--	--
Gene|93867::TRINITY_DN55_c0_g1::g.93867::m.93867|DN55_c0_g1:122-532(+)	6	6	15327	DNA Binding – Histone	--	K11253|8.3e-67|mdo:100011902|K11253 histone H3 | (RefSeq) uncharacterized LOC100011902	Core histone H2A/H2B/H3/H4	Histone H3.3A OS=Drosophila melanogaster OX=7227 GN=His3.3A PE=1 SV=1	B
Gene|25376::TRINITY_DN144491_c0_g1::g.25376::m.25376|DN144491_c0_g1:100-1434(-)	6	6	51556	Unknown	--	--	--	--	--
Gene|67281::TRINITY_DN34488_c0_g1::g.67281::m.67281|DN34488_c0_g1:136-786(+)	6	5	23919	Unknown	--	--	--	--	--
Gene|53847::TRINITY_DN26340_c1_g1::g.53847::m.53847|DN26340_c1_g1:2-1021(+)	6	5	39307	Protein Glycosylation	--	K00718|3.2e-28|zne:110826935|K00718 galactoside 2-L-fucosyltransferase 1/2 [EC:2.4.1.69] | (RefSeq) galactoside 2-alpha-L-fucosyltransferase 2-like	Glycosyl transferase family 11	--	G
Gene|89557::TRINITY_DN52028_c0_g1::g.89557::m.89557|DN52028_c0_g1:3-248(-)	6	4	9396	Unknown	--	--	--	--	--
Gene|48825::TRINITY_DN2360_c1_g1::g.48825::m.48825|DN2360_c1_g1:1-1716(+)	5	5	61671	ATP binding - ATP synthase	[C]	K02132|1.5e-287|zne:110840550|K02132 F-type H+-transporting ATPase subunit alpha | (RefSeq) ATP synthase subunit alpha, mitochondrial	ATP synthase alpha/beta family, nucleotide-binding domain;; ATP synthase alpha/beta chain, C terminal domain;; ATP synthase alpha/beta family, beta-barrel domain	ATP synthase subunit alpha, mitochondrial OS=Drosophila melanogaster OX=7227 GN=blw PE=1 SV=2	C
Gene|35274::TRINITY_DN17425_c0_g1::g.35274::m.35274|DN17425_c0_g1:1-1260(-)	5	5	44808	Unknown	--	--	--	--	--
Gene|68184::TRINITY_DN3505_c0_g1::g.68184::m.68184|DN3505_c0_g1:55-777(+)	5	5	26420	Unknown	--	--	--	--	--
Gene|115476::TRINITY_DN8048_c0_g1::g.115476::m.115476|DN8048_c0_g1:104-532(+)	5	5	15412	Transcription Activator	--	--	Transcription activator MBF2	--	--
Gene|123671::TRINITY_DN92162_c0_g1::g.123671::m.123671|DN92162_c0_g1:1-426(-)	5	3	16091	Hydrolase activity - Lipid Metabolism	--	K01771|2.9e-23|fcd:110844986|K01771 1-phosphatidylinositol phosphodiesterase [EC:4.6.1.13] | (RefSeq) uncharacterized protein LOC110844986	--	--	T
Gene|1053::TRINITY_DN1015_c0_g1::g.1053::m.1053|DN1015_c0_g1:3-368(-)	5	3	13472	Hydrolase activity - Carbohydrate metabolism	--	K00771|8.0e-06|prap:110992106|K00771 protein xylosyltransferase [EC:2.4.2.26] | (RefSeq) xylosyltransferase oxt	WSC domain	--	--
Gene|49970::TRINITY_DN24104_c0_g2::g.49970::m.49970|DN24104_c0_g2:2-313(+)	5	1	11664	ATP binding – Actin	[Z]	K05692|2.3e-54|aag:5574526|K05692 actin beta/gamma 1 | (RefSeq) actin-5C	Actin	Actin-5C OS=Drosophila melanogaster OX=7227 GN=Act5C PE=1 SV=4	Z
Gene|94739::TRINITY_DN5701_c0_g1::g.94739::m.94739|DN5701_c0_g1:2-427(+)	5	1	15918	Unknown	--	--	--	--	--
Gene|122583::TRINITY_DN90435_c0_g1::g.122583::m.122583|DN90435_c0_g1:123-1100(+)	4	4	35492	Hydrolase Activity - Protease	[O]	K09632|5.7e-40|dhe:111601089|K09632 chymotrypsin-like protease [EC:3.4.21.-] | (RefSeq) serine protease 3-like	Trypsin	Chymotrypsin BI OS=Penaeus vannamei OX=6689 PE=1 SV=1	O
Gene|63749::TRINITY_DN32320_c0_g2::g.63749::m.63749|DN32320_c0_g2:45-671(-)	4	4	23109	Unknown	--	--	--	--	--
Gene|124737::TRINITY_DN93_c0_g1::g.124737::m.124737|DN93_c0_g1:3-728(-)	4	4	26502	Xylosyltransferase - Gram-negative Bacteria Binding	--	K00771|1.5e-08|fcd:110848438|K00771 protein xylosyltransferase [EC:2.4.2.26] | (RefSeq) xylosyltransferase oxt-like	WSC domain	--	S
Gene|68129::TRINITY_DN35000_c0_g1::g.68129::m.68129|DN35000_c0_g1:36-683(+)	4	4	23244	Unknown	--	--	--	--	--
Gene|29116::TRINITY_DN151412_c7_g1::g.29116::m.29116|DN151412_c7_g1:3-425(+)	4	4	15292	DNA Binding – Histone	--	K11251|1.2e-53|aag:110678260|K11251 histone H2A | (RefSeq) histone H2A-like	C-terminus of histone H2A;; Core histone H2A/H2B/H3/H4;; Histone-like transcription factor (CBF/NF-Y) and archaeal histone	Histone H2A OS=Drosophila erecta OX=7220 GN=His2A PE=3 SV=2	B
Gene|2656::TRINITY_DN10399_c0_g2::g.2656::m.2656|DN10399_c0_g2:3-971(-)	4	4	36694	Unknown	--	--	--	--	--
Gene|11439::TRINITY_DN11924_c0_g1::g.11439::m.11439|DN11924_c0_g1:83-487(+)	4	4	14591	Transcription Activator - Probable Salivary Secreted Peptide	--	--	Transcription activator MBF2	--	S
Gene|123331::TRINITY_DN9149_c0_g1::g.123331::m.123331|DN9149_c0_g1:1-423(+)	4	3	14951	Hydrolase Activity -	--	--	Glycoside hydrolase family 5 C-terminal domain	--	--

			Carbohydrate metabolism						
Gene|16116::TRINITY_DN1274_c1_g3::g.16116::m.16116|DN1274_c1_g3:2-1642(+)	4	2	60103	Hydrolase activity - Carbohydrate metabolism	--	K00771|2.7e-13|aag:5569146|K00771 protein xylosyltransferase [EC:2.4.2.26] | (RefSeq) xylosyltransferase oxt	WSC domain;; EGF-like domain	--	--
Gene|8808::TRINITY_DN114795_c0_g1::g.8808::m.8808|DN114795_c0_g1:3-665(-)	3	3	23801	Structural - Ribosome	[J]	K03231|4.1e-117|pcf:106790711|K03231 elongation factor 1-alpha | (RefSeq) elongation factor 1-alpha	Elongation factor Tu C-terminal domain;; Elongation factor Tu domain 2	Elongation factor 1-alpha OS=Bombyx mori OX=7091 PE=2 SV=1	J
Gene|105675::TRINITY_DN68507_c0_g1::g.105675::m.105675|DN68507_c0_g1:2-814(-)	3	3	29612	Structural - Ribosome	[J]	K03231|5.2e-106|nai:NECAME_04258|K03231 elongation factor 1-alpha | (RefSeq) translation elongation factor EF-1, subunit alpha	Elongation factor Tu C-terminal domain;; Elongation factor Tu domain 2	Elongation factor 1-alpha OS=Tetrahymena pyriformis OX=5908 PE=2 SV=1	J
Gene|59666::TRINITY_DN2988_c0_g1::g.59666::m.59666|DN2988_c0_g1:1-540(+)	3	3	20391	Unknown	--	--	--	--	--
Gene|47672::TRINITY_DN2296_c0_g1::g.47672::m.47672|DN2296_c0_g1:92-850(-)	3	3	28086	Unknown	--	--	--	--	--
Gene|28090::TRINITY_DN149634_c0_g1::g.28090::m.28090|DN149634_c0_g1:393-2015(-)	3	3	57598	ATP binding - ATP synthase	[C]	K02133|8.5e-267|clec:106660842|K02133 F-type H+-transporting ATPase subunit beta [EC:7.1.2.2] | (RefSeq) ATP synthase subunit beta, mitochondrial	ATP synthase alpha/beta family, nucleotide-binding domain;; ATP synthase alpha/beta family, beta-barrel domain	ATP synthase subunit beta, mitochondrial OS=Drosophila melanogaster OX=7227 GN=ATPsynbeta PE=1 SV=3	C
Gene|54186::TRINITY_DN26585_c0_g1::g.54186::m.54186|DN26585_c0_g1:1-468(+)	3	3	17862	Metal Iron Binding	--	K16141|1.9e-84|hsa:213|K16141 serum albumin | (RefSeq) ALB, HSA, PRO0883, PRO0903, PRO1341; albumin	Serum albumin family	--	--
Gene|44311::TRINITY_DN2142_c0_g1::g.44311::m.44311|DN2142_c0_g1:3-269(+)	3	3	9217	unknown	--	--	--	--	--
Gene|30648::TRINITY_DN1541_c0_g1::g.30648::m.30648|DN1541_c0_g1:2-253(+)	3	2	8883	unknown	--	--	--	--	--
Gene|99411::TRINITY_DN617_c0_g1::g.99411::m.99411|DN617_c0_g1:464-12481(-)	2	2	451892	Calcium ion binding - Microtubule binding	--	K10382|0.0e+00|zne:110831493|K10382 dystonin | (RefSeq) dystonin isoform X1	Plectin repeat;; Calponin homology (CH) domain;; Spectrin repeat;; SH3 domain;; OCRE domain;; CAMSAP CH domain;; Spectrin repeat;; Growth-Arrest-Specific Protein 2 Domain;; EF-hand domain pair;; EF-hand domain;; EF hand	Spectrin beta chain OS=Drosophila melanogaster OX=7227 GN=beta-Spec PE=1 SV=2	Z
Gene|69793::TRINITY_DN36110_c0_g1::g.69793::m.69793|DN36110_c0_g1:283-720(-)	2	2	16223	DNA Binding	--	--	BESS motif	--	--
Gene|59599::TRINITY_DN2982_c0_g1::g.59599::m.59599|DN2982_c0_g1:2-370(-)	2	2	13605	Hydrolase Activity - Carbohydrate metabolism	--	K00771|2.5e-15|aag:5569146|K00771 protein xylosyltransferase [EC:2.4.2.26] | (RefSeq) xylosyltransferase oxt	WSC domain	--	--
Gene|41192::TRINITY_DN20062_c1_g3::g.41192::m.41192|DN20062_c1_g3:3-1508(+)	2	2	54241	ATP binding - ATP synthase	[C]	K02133|2.0e-173|dre:100038785|K02133 F-type H+-transporting ATPase subunit beta [EC:7.1.2.2] | (RefSeq) zgc:163069; F0F1 ATP synthase subunit beta	ATP synthase alpha/beta family, nucleotide-binding domain;; ATP synthase alpha/beta family, beta-barrel domain	ATP synthase subunit beta, mitochondrial OS=Drosophila melanogaster OX=7227 GN=ATPsynbeta PE=1 SV=3	C
Gene|109233::TRINITY_DN728_c2_g1::g.109233::m.109233|DN728_c2_g1:54-425(+)	2	2	13635	ATP Binding – Histone	--	K11252|4.6e-57|obo:105280750|K11252 histone H2B | (RefSeq) histone H2B	Core histone H2A/H2B/H3/H4;; Histone-like transcription factor (CBF/NF-Y) and archaeal histone	Histone H2B.1/H2B.2 OS=Tigriopus californicus OX=6832 PE=3 SV=2	B
Gene|39434::TRINITY_DN19253_c0_g1::g.39434::m.39434|DN19253_c0_g1:44-1282(+)	2	2	46993	Melanin Metabolic Process	--	K22203|1.6e-84|nvl:108566790|K22203 dopachrome tautomerase [EC:5.3.3.12] | (RefSeq) L-dopachrome tautomerase yellow-f2-like	Major royal jelly protein	Protein yellow OS=Drosophila subobscura OX=7241 GN=y PE=3 SV=1	G
Gene|111572::TRINITY_DN7532_c0_g1::g.111572::m.111572|DN7532_c0_g1:105-491(+)	2	2	13419	ATP Binding – Histone	--	K11251|2.2e-63|aec:105154673|K11251 histone H2A | (RefSeq) histone H2A.V	Core histone H2A/H2B/H3/H4;; C-terminus of histone H2A	Histone H2A.V (Fragment) OS=Strongylocentrotus purpuratus OX=7668	B

								GN=H2A.F/Z PE=1 SV=1	
Gene|97045::TRINITY_DN596_c5_g1::g.97045::m.97045|DN596_c5_g1:192-1394(-)	2	2	45281	Structural – Vesicle	--	K20367|6.1e-124|clec:106672770|K20367 endoplasmic reticulum-Golgi intermediate compartment protein 3 | (RefSeq) endoplasmic reticulum-Golgi intermediate compartment protein 3 isoform X1	Endoplasmic reticulum vesicle transporter;; Endoplasmic Reticulum-Golgi Intermediate Compartment (ERGIC)	Probable endoplasmic reticulum-Golgi intermediate compartment protein 3 OS=Dictyostelium discoideum OX=44689 GN=ergic3 PE=3 SV=1	U
Gene|41187::TRINITY_DN20062_c1_g1::g.41187::m.41187|DN20062_c1_g1:2-1018(+)	2	2	36125	ATP Binding - ATP Synthase	[C]	K02112|1.8e-108|isc:IscW_ISCW016070|K02112 F-type H+/Na+-transporting ATPase subunit beta [EC:7.1.2.2 7.2.2.1] | (RefSeq) ATP synthase subunit alpha, sodium ion specific, putative	ATP synthase alpha/beta family, nucleotide-binding domain;; ATP synthase alpha/beta family, beta-barrel domain	ATP synthase subunit beta, mitochondrial OS=Drosophila melanogaster OX=7227 GN=ATPsynbeta PE=1 SV=3	C
Gene|13620::TRINITY_DN1227_c0_g2::g.13620::m.13620|DN1227_c0_g2:1-312(-)	2	1	11606	Hydrolase Activity-Carbohydrate metabolism	--	K22144|8.6e-09|lgi:LOTGIDRAFT_160629|K22144 cell surface hyaluronidase [EC:3.2.1.35] | (RefSeq) hypothetical protein	WSC domain	--	U
Gene|101940::TRINITY_DN64294_c0_g1::g.101940::m.101940|DN64294_c0_g1:2-292(-)	2	1	11187	Hydrolase activity - Lipid Metabolism	--	K01771|6.3e-14|hmg:105846403|K01771 1-phosphatidylinositol phosphodiesterase [EC:4.6.1.13] | (RefSeq) uncharacterized LOC105846403	--	--	--
Gene|75398::TRINITY_DN39954_c0_g1::g.75398::m.75398|DN39954_c0_g1:2-475(-)	2	1	17964	Hydrolase activity - Lipid Metabolism	--	K01771|6.3e-14|hmg:105846403|K01771 1-phosphatidylinositol phosphodiesterase [EC:4.6.1.13] | (RefSeq) uncharacterized LOC105846403	--	--	--
Gene|44006::TRINITY_DN21291_c0_g1::g.44006::m.44006|DN21291_c0_g1:72-2246(-)	1	1	84109	Structural - Ribosome	--	K14856|2.1e-240|zne:110841523|K14856 protein SDA1 | (RefSeq) protein SDA1 homolog	SDA1;; NUC130/3NT domain	Protein SDA1 homolog OS=Drosophila melanogaster OX=7227 GN=Mys45A PE=1 SV=1	--
Gene|70051::TRINITY_DN36347_c0_g1::g.70051::m.70051|DN36347_c0_g1:3-362(-)	1	1	12931	Hydrolase activity - Lipid Metabolism	[I]	K03927|3.5e-38|api:100163376|K03927 carboxylesterase 2 [EC:3.1.1.1 3.1.1.84 3.1.1.56] | (RefSeq) esterase E4 isoform X1	Carboxylesterase family;; Prolyl oligopeptidase family;; alpha/beta hydrolase fold	Esterase FE4 OS=Myzus persicae OX=13164 PE=1 SV=1	G
Gene|97354::TRINITY_DN5_c3_g1::g.97354::m.97354|DN5_c3_g1:1-600(-)	1	1	23052	Amino acid metabolismo	[E]	--	Aspartate/ornithine carbamoyltransferase, carbamoyl-P binding domain	--	--
Gene|40922::TRINITY_DN1992_c0_g1::g.40922::m.40922|DN1992_c0_g1:598-5478(-)	1	1	182198	Unknown	--	--	Putative zinc-finger domain	--	--
Gene|71935::TRINITY_DN3788_c0_g1::g.71935::m.71935|DN3788_c0_g1:314-4681(+)	1	1	168594	Endonuclease	--	K06461|4.5e-86|sfm:114909219|K06461 integrin alpha M | (RefSeq) integrin alpha-M-like	RNase H-like domain found in reverse transcriptase;; RNase H-like domain found in reverse transcriptase;; Reverse transcriptase (RNA-dependent DNA polymerase);; Integrase zinc binding domain;; Integrase core domain;; Zinc knuckle	Retrovirus-related Pol polyprotein from transposon 412 OS=Drosophila melanogaster OX=7227 GN=POL PE=4 SV=1	E
Gene|116937::TRINITY_DN8265_c0_g3::g.116937::m.116937|DN8265_c0_g3:646-4407(+)	1	1	145514	Unknown	--	K20473|1.6e-26|hmg:100215633|K20473 neuroblastoma-amplified sequence | (RefSeq) nbas; neuroblastoma-amplified sequence	Recombination endonuclease VII	--	--
Gene|113908::TRINITY_DN782_c0_g1::g.113908::m.113908|DN782_c0_g1:278-2080(+)	1	1	67875	GTPase-Activating Protein	--	K16733|5.0e-128|ame:410144|K16733 Rac GTPase-activating protein 1 | (RefSeq) rac GTPase-activating protein 1	RhoGAP domain;; Phorbol esters/diacylglycerol binding domain (C1 domain)	GTPase-activating protein RacGAP84C OS=Drosophila melanogaster OX=7227 GN=RacGAP84C PE=2 SV=1	T
Gene|127197::TRINITY_DN9817_c0_g1::g.127197::m.127197|DN9817_c0_g1:418-2493(-)	1	1	79071	Hydrolase Activity-Carbohydrate metabolism	[G]	K12309|4.4e-96|ame:725756|K12309 beta-galactosidase [EC:3.2.1.23] | (RefSeq) beta-galactosidase	Glycosyl hydrolases family 35;; Beta-galactosidase	Beta-galactosidase 1 OS=Dictyostelium discoideum OX=44689 GN=glb1 PE=3 SV=1	G
Gene|30988::TRINITY_DN15579_c0_g2::g.30988::m.30988|DN15579_c0_g2:202-678(-)	1	1	17817	Monooxygenase activity- iron ion binding	[QV]	K15001|1.4e-35|zne:110831624|K15001 cytochrome P450 family 4 [EC:1.14.-.-] | (RefSeq) cytochrome P450 4C1-like	Cytochrome P450	Cytochrome P450 4c3 OS=Drosophila	Q

								melanogaster OX=7227 GN=Cyp4c3 PE=2 SV=1	
Gene|124425::TRINITY_DN9345_c0_g1::g.124425::m.124425|DN9345_c0_g1:87-2447(+)	1	1	91148	Vesicle docking involved in exocytosis	--	K19985|0.0e+00|zne:110839359|K19985 exocyst complex component 6 | (RefSeq) exocyst complex component 6B isoform X1	Exocyst complex subunit Sec15-like	Exocyst complex component 6 OS=Drosophila melanogaster OX=7227 GN=Sec15 PE=1 SV=1	U
Gene|14682::TRINITY_DN124807_c1_g1::g.14682::m.14682|DN124807_c1_g1:210-1736(-)	1	1	58492	Monooxygenase activity- iron ion binding	[QV]	K15001|5.5e-97|zne:110836931|K15001 cytochrome P450 family 4 [EC:1.14.-.-] | (RefSeq) cytochrome P450 4C1-like isoform X1	Cytochrome P450	Cytochrome P450 4d2 OS=Drosophila melanogaster OX=7227 GN=Cyp4d2 PE=2 SV=2	Q
Gene|122335::TRINITY_DN89953_c0_g2::g.122335::m.122335|DN89953_c0_g2:504-665(-)	1	1	6314	Unknown	--	--	--	--	--
Gene|82442::TRINITY_DN4561_c0_g1::g.82442::m.82442|DN4561_c0_g1:125-13252(+)	1	1	490627	actin binding	--	K14539|2.3e-29|egl:EGR_08670|K14539 large subunit GTPase 1 [EC:3.6.1.-] | (RefSeq) Large subunit GTPase	Smoothelin cytoskeleton protein;; Calponin homology (CH) domain;; CAMSAP CH domain	Interaptin OS=Dictyostelium discoideum OX=44689 GN=abpD PE=1 SV=1	Z
Gene|117495::TRINITY_DN83414_c0_g1::g.117495::m.117495|DN83414_c0_g1:742-2628(-)	1	1	72218	UV stimulated scaffold protein A	--	K23720|4.6e-154|zne:110830215|K23720 UV-stimulated scaffold protein A | (RefSeq) UV-stimulated scaffold protein A-like	Uncharacterized conserved protein (DUF2043)	--	S
Gene|60771::TRINITY_DN30373_c0_g1::g.60771::m.60771|DN30373_c0_g1:271-1686(-)	1	1	54668	Calcium ion binding	--	--	Protein of unknown function (DUF1682)	--	S
Gene|32169::TRINITY_DN16058_c0_g1::g.32169::m.32169|DN16058_c0_g1:228-4715(+)	1	1	169633	Unknown	--	K22909|5.7e-91|mpha:105836898|K22909 zinc finger CCCH domain-containing protein 13 | (RefSeq) uncharacterized protein CG7065	--	Uncharacterized protein CG7065 OS=Drosophila melanogaster OX=7227 GN=CG7065 PE=1 SV=1	--
Gene|109751::TRINITY_DN73490_c0_g3::g.109751::m.109751|DN73490_c0_g3:2-490(+)	1	1	18303	DNA binding - ATP binding	[L]	K02469|9.1e-13|spis:111345986|K02469 DNA gyrase subunit A [EC:5.6.2.2] | (RefSeq) uncharacterized protein LOC111345986	DNA gyrase/topoisomerase IV, subunit A	--	B
Gene|39794::TRINITY_DN19395_c0_g2::g.39794::m.39794|DN19395_c0_g2:113-4498(+)	1	1	159998	Catalytic activity cytoskeleton - tubulin binding	--	K10404|1.2e-303|zne:110834177|K10404 kinesin family member 26 | (RefSeq) kinesin-like protein GA13060 isoform X1	Kinesin motor domain;; Microtubule binding	Kinesin-like protein CG14535 OS=Drosophila melanogaster OX=7227 GN=CG14535 PE=2 SV=2	Z
Gene|69580::TRINITY_DN3595_c0_g1::g.69580::m.69580|DN3595_c0_g1:160-1338(+)	1	1	44434	Immune response	--	K16678|5.1e-06|zne:110828011|K16678 eiger | (RefSeq) uncharacterized protein LOC110828011 isoform X1	TNF(Tumour Necrosis Factor) family	--	--
Gene|30710::TRINITY_DN15445_c0_g1::g.30710::m.30710|DN15445_c0_g1:3-938(-)	1	1	36111	Monooxygenase activity- iron ion binding	--	K15001|1.8e-60|zne:110837214|K15001 cytochrome P450 family 4 [EC:1.14.-.-] | (RefSeq) cytochrome P450 4C1-like	Cytochrome P450	Cytochrome P450 4C1 OS=Blaberus discoidalis OX=6981 GN=CYP4C1 PE=2 SV=1	Q
Gene|96371::TRINITY_DN588_c0_g1::g.96371::m.96371|DN588_c0_g1:2-4906(-)	1	1	187158	Unknown	--	--	--	--	--
Gene|50713::TRINITY_DN24528_c0_g3::g.50713::m.50713|DN24528_c0_g3:2-1054(+)	1	1	39855	Integral component of membrane	--	--	Protein of unknown function (DUF1676)	--	S
Gene|24673::TRINITY_DN142552_c0_g1::g.24673::m.24673|DN142552_c0_g1:116-1726(-)	1	1	62004	Monooxygenase activity- iron ion binding	[QV]	K14999|7.3e-103|zne:110831538|K14999 cytochrome P450 family 6 [EC:1.14.-.-] | (RefSeq) uncharacterized protein LOC110831538	Cytochrome P450	Cytochrome P450 6a2 OS=Drosophila melanogaster OX=7227 GN=Cyp6a2 PE=2 SV=2	Q
Gene|74414::TRINITY_DN3947_c0_g1::g.74414::m.74414|DN3947_c0_g1:2819-4771(-)	1	1	73950	Glutamate-cysteine ligase activity	--	K11204|6.6e-300|zne:110834233|K11204 glutamate--cysteine ligase catalytic subunit [EC:6.3.2.2] | (RefSeq) glutamate--cysteine ligase catalytic subunit	Glutamate-cysteine ligase	Glutamate--cysteine ligase OS=Drosophila melanogaster OX=7227 GN=Gclc PE=2 SV=1	H
Gene|56104::TRINITY_DN27566_c0_g1::g.56104::m.56104|DN27566_c0_g1:222-2051(-)	1	1	69483	Nucleic acid binding - DNA metabolic process	--	K10884|1.2e-117|zne:110831579|K10884 ATP-dependent DNA helicase 2 subunit 1 | (RefSeq) X-ray repair cross-complementing protein 6	Ku70/Ku80 beta-barrel domain;; Ku70/Ku80 N-terminal alpha/beta domain;; Ku70/Ku80 C-terminal arm	ATP-dependent DNA helicase ku70 OS=Dictyostelium discoideum OX=44689 GN=ku70 PE=3 SV=1	L
Gene|22707::TRINITY_DN13810_c0_g1::g.22707::m.22707|DN13810_c0_g1:184-1599(-)	1	1	52550	Oxidase activity	[H]	K00231|9.2e-137|zne:110834755|K00231 protoporphyrinogen/coproporphyrinogen III oxidase [EC:1.3.3.4 1.3.3.15] | (RefSeq) protoporphyrinogen oxidase	Flavin containing amine oxidoreductase;; NAD(P)-binding Rossmann-like domain	--	H

Gene|33249::TRINITY_DN1644_c0_g1::g.33249::m.33249|DN1644_c0_g1:1138-4080(-)	1	1	113455	Peptidase	[E]	K11140|7.3e-103|soc:105205290|K11140 aminopeptidase N [EC:3.4.11.2] | (RefSeq) aminopeptidase N	Peptidase family M1 domain;; ERAP1-like C-terminal domain;; Peptidase M1 N-terminal domain	Membrane alanyl aminopeptidase OS=Heliothis virescens OX=7102 PE=1 SV=1	--
Gene|95308::TRINITY_DN57667_c0_g4::g.95308::m.95308|DN57667_c0_g4:112-1044(+)	1	1	36052	Lipid metabolism	--	K19625|9.2e-39|ags:114132133|K19625 retinaldehyde-binding protein 1 | (RefSeq) alpha-tocopherol transfer protein	CRAL/TRIO domain;; CRAL/TRIO, N-terminal domain;; Divergent CRAL/TRIO domain	--	I
Gene|118859::TRINITY_DN85186_c0_g1::g.118859::m.118859|DN85186_c0_g1:1-891(+)	1	1	34552	Unknown	--	--	--	--	--
Gene|115361::TRINITY_DN80237_c0_g1::g.115361::m.115361|DN80237_c0_g1:2-742(-)	1	1	25853	ATP binding - ATP Synthase	[C]	K02111|5.0e-68|isc:IscW_ISCW016068|K02111 F-type H+/Na+-transporting ATPase subunit alpha [EC:7.1.2.2 7.2.2.1] | (RefSeq) F0F1-type ATP synthase, alpha subunit, putative	ATP synthase alpha/beta family, nucleotide-binding domain;; ATP synthase alpha/beta family, beta-barrel domain;; ATP synthase A chain;; ATP synthase delta (OSCP) subunit;; ATP synthase B/B' CF(0);; ATP synthase subunit C	ATP synthase subunit alpha, mitochondrial OS=Drosophila melanogaster OX=7227 GN=blw PE=1 SV=2	C
Gene|20855::TRINITY_DN134540_c0_g1::g.20855::m.20855|DN134540_c0_g1:3-248(+)	1	1	9465	Amino acid metabolismo	[E]	--	--	--	--
Gene|29652::TRINITY_DN152210_c0_g1::g.29652::m.29652|DN152210_c0_g1:28-282(-)	1	1	10068	Structural - Ribosome	[J]	--	Ribosomal protein S15	--	--
Gene|47299::TRINITY_DN22748_c0_g1::g.47299::m.47299|DN22748_c0_g1:78-1163(+)	1	1	41140	RNA Synthase	--	K22537|4.3e-53|clec:106666541|K22537 mitochondrial RNA pseudouridine synthase RPUSD3 [EC:5.4.99.-] | (RefSeq) mitochondrial RNA pseudouridine synthase rpusd4-like	RNA pseudouridylate synthase	--	A
Gene|2383::TRINITY_DN10363_c0_g1::g.2383::m.2383|DN10363_c0_g1:1-936(+)	1	1	34435	Hydrolase Activity - Protease	[O]	K09640|3.9e-80|zne:110838471|K09640 transmembrane protease serine 9 [EC:3.4.21.-] | (RefSeq) transmembrane protease serine 9-like	Trypsin;; Trypsin-like peptidase domain	Proclotting enzyme OS=Tachypleus tridentatus OX=6853 PE=1 SV=1	O
Gene|125379::TRINITY_DN95101_c0_g1::g.125379::m.125379|DN95101_c0_g1:222-6155(+)	1	1	227365	GTPase binding - Cytoteskeleton	--	K11577|4.4e-221|zne:110827191|K11577 kinetochore-associated protein 1 | (RefSeq) kinetochore-associated protein 1 isoform X1	Rough deal protein C-terminal region	--	D
Gene|8242::TRINITY_DN11353_c0_g1::g.8242::m.8242|DN11353_c0_g1:583-4074(-)	1	1	129914	G protein-coupled receptor activity - protein-hormone receptor activity	--	K08399|0.0e+00|clec:106664139|K08399 leucine-rich repeat-containing G protein-coupled receptor 6 | (RefSeq) lutropin-choriogonadotropic hormone receptor isoform X1	Leucine rich repeat;; Leucine Rich repeats (2 copies);; 7 transmembrane receptor (rhodopsin family);; BspA type Leucine rich repeat region (6 copies);; Leucine-rich repeat;; Leucine Rich Repeat	Probable glycoprotein hormone G-protein coupled receptor OS=Anthopleura elegantissima OX=6110 PE=2 SV=1	T
Gene|15229::TRINITY_DN125777_c0_g1::g.15229::m.15229|DN125777_c0_g1:541-9144(-)	1	1	320634	Calcium ion binding	[S]	K16506|2.7e-304|pdam:113679474|K16506 protocadherin Fat 1/2/3 | (RefSeq) protocadherin-like protein	Cadherin domain;; Laminin G domain;; Cadherin cytoplasmic region;; Laminin G domain;; Cadherin-like;; EGF-like domain;; RET Cadherin like domain 1;; Cadherin prodomain like;; Human growth factor-like EGF;; Cadherin-like	Neural-cadherin OS=Drosophila melanogaster OX=7227 GN=CadN PE=1 SV=2	S
Gene|64061::TRINITY_DN3253_c2_g2::g.64061::m.64061|DN3253_c2_g2:310-2376(+)	1	1	77868	Unknown	--	K14548|4.3e-218|zne:110829122|K14548 U3 small nucleolar RNA-associated protein 4 | (RefSeq) U3 small nucleolar RNA-associated protein 4 homolog	WD domain, G-beta repeat;; Anaphase-promoting complex subunit 4 WD40 domain	--	S
Gene|34230::TRINITY_DN16908_c0_g1::g.34230::m.34230|DN16908_c0_g1:205-1614(+)	1	1	54581	Hydrolase Activity-Carbohydrate metabolism	[G]	K01229|6.2e-127|egz:104129535|K01229 lactase-phlorizin hydrolase [EC:3.2.1.108 3.2.1.62] | (RefSeq) LCT; lactase-phlorizin hydrolase	Glycosyl hydrolase family 1	Myrosinase 1 OS=Brevicoryne brassicae OX=69196 PE=1 SV=1	G
Gene|86414::TRINITY_DN491_c3_g1::g.86414::m.86414|DN491_c3_g1:289-912(-)	1	1	23444	DNA binding - Histone	--	K11253|1.5e-25|zne:110840599|K11253 histone H3 | (RefSeq) histone H3-like centromeric protein A	Core histone H2A/H2B/H3/H4	Histone H3.3 type c OS=Dictyostelium discoideum OX=44689 GN=H3c PE=1 SV=1	B

Supplementary Table S2. Differentially abundant (up-regulated) proteins identified in the foam produced by *Mahanarva spectabilis* in PIO (elephant grass cv. Pioneiro) versus ROXO (elephant grass cv. Roxo de Botucatu), including fold change (FC) values, statistical significance, GO functional annotations, and TrEMBL and nr descriptions.

Gene	FC	log2(FC)	raw.pval	-log10(p)	GO_annotation	TrEMBL_annotation	nr_annotation
Gene|97354::DN5_c3_g1::g.97354::m.97354|DN5_c3_g1:1-600(-)	2.4809	1.3109	0.078066	1.1075	Biological Process: cellular amino acid metabolic process (GO:0006520);; Molecular Function: amino acid binding (GO:0016597);; Molecular Function: carboxyl- or carbamoyltransferase activity (GO:0016743);;	Uncharacterized protein OS=Homalodisca liturata OX=320908 GN=g.3327 PE=3 SV=1	hypothetical protein FQR65_LT17659 [Abscondita terminalis]
Gene|20855::DN134540_c0_g1::g.20855::m.20855|DN134540_c0_g1:3-248(+)	2.4809	1.3109	0.078066	1.1075	Biological Process: cellular amino acid metabolic process (GO:0006520);; Molecular Function: amino acid binding (GO:0016597);; Molecular Function: carboxyl- or carbamoyltransferase activity (GO:0016743);;	Uncharacterized protein OS=Homalodisca liturata OX=320908 GN=g.3327 PE=3 SV=1	--
Gene|49975::DN24104_c2_g1::g.49975::m.49975|DN24104_c2_g1:139-1269(+)	1.3605	0.4441	0.03494	1.4567	Molecular Function: ATP binding (GO:0005524);; Cellular Component: cytoplasm (GO:0005737);; Cellular Component: cytoskeleton (GO:0005856);;	Uncharacterized protein OS=Cuerna arida OX=1464854 GN=g.14203 PE=3 SV=1	actin related protein 1 [Acyrthosiphon pisum]
Gene|48825::DN2360_c1_g1::g.48825::m.48825|DN2360_c1_g1:1-1716(+)	3.6182	1.8553	0.0027469	2.5612	Molecular Function: ATP binding (GO:0005524);; Biological Process: ATP synthesis coupled proton transport (GO:0015986);; Cellular Component: proton-transporting ATP synthase complex, catalytic core F(1) (GO:0045261);; Molecular Function: proton-transporting ATP synthase activity, rotational mechanism (GO:0046933);;	ATP synthase subunit alpha (Fragment) OS=Clastoptera arizonana OX=38151 GN=g.38453 PE=3 SV=1	ATP synthase subunit alpha, mitochondrial [Cryptotermes secundus]
Gene|8808::DN114795_c0_g1::g.8808::m.8808|DN114795_c0_g1:3-665(-)	3.2274	1.6904	0.00039816	3.3999	Molecular Function: translation elongation factor activity (GO:0003746);; Molecular Function: GTPase activity (GO:0003924);; Molecular Function: GTP binding (GO:0005525);;	Elongation factor 1-alpha OS=Oryctes borbonicus OX=1629725 GN=AMK59_4534 PE=3 SV=1	hypothetical protein AMK59_4534 [Oryctes borbonicus]
Gene|105675::DN68507_c0_g1::g.105675::m.105675|DN68507_c0_g1:2-814(-)	3.2274	1.6904	0.00039816	3.3999	Molecular Function: translation elongation factor activity (GO:0003746);; Molecular Function: GTPase activity (GO:0003924);; Molecular Function: GTP binding (GO:0005525);;	Elongation factor 1-alpha OS=Naegleria gruberi OX=5762 GN=NAEGRDRAFT_56672 PE=3 SV=1	predicted protein [Naegleria gruberi strain NEG-M]
Gene|104521::DN66_c0_g1::g.104521::m.104521|DN66_c0_g1:101-1489(+)	3.3866	1.7598	0.00021437	3.6688	Molecular Function: translation elongation factor activity (GO:0003746);; Molecular Function: GTPase activity (GO:0003924);; Molecular Function: GTP binding (GO:0005525);;	Elongation factor 1-alpha OS=Clastoptera arizonana OX=38151 GN=g.6817 PE=3 SV=1	Hypothetical predicted protein [Cloeon dipterum]
Gene|9962::DN116_c0_g1::g.9962::m.9962|DN116_c0_g1:695-2047(-)	4.5632	2.1901	0.0035848	2.4455	Molecular Function: GTPase activity (GO:0003924);; Molecular Function: structural constituent of cytoskeleton (GO:0005200);; Molecular Function: GTP binding (GO:0005525);; Cellular Component: cytoplasm (GO:0005737);; Cellular Component: microtubule (GO:0005874);; Biological Process: microtubule-based process (GO:0007017);;	Tubulin alpha chain OS=Anopheles atroparvus OX=41427 PE=3 SV=1	tubulin [Schistocerca americana]
Gene|125314::DN94_c0_g1::g.125314::m.125314|DN94_c0_g1:125-1477(+)	4.5632	2.1901	0.0035848	2.4455	Molecular Function: GTPase activity (GO:0003924);; Molecular Function: structural constituent of cytoskeleton (GO:0005200);; Molecular Function: GTP binding (GO:0005525);; Cellular Component: cytoplasm (GO:0005737);; Cellular Component: microtubule (GO:0005874);; Biological Process: microtubule-based process (GO:0007017);;	Tubulin alpha chain OS=Laodelphax striatellus OX=195883 GN=TUA2 PE=2 SV=1	tubulin alpha-1 chain [Nilaparvata lugens]
Gene|125317::DN94_c1_g1::g.125317::m.125317|DN94_c1_g1:1-1149(-)	4.5632	2.1901	0.0035848	2.4455	Molecular Function: GTPase activity (GO:0003924);; Molecular Function: structural constituent of cytoskeleton (GO:0005200);; Molecular Function: GTP binding (GO:0005525);; Cellular Component: microtubule (GO:0005874);; Biological Process: microtubule-based process (GO:0007017);;	Tubulin alpha chain (Fragment) OS=Homalodisca liturata OX=320908 GN=g.17932 PE=3 SV=1	PREDICTED: tubulin alpha-1C chain [Diuraphis noxia]
Gene|106927::DN702_c0_g1::g.106927::m.106927|DN702_c0_g1:258-1313(+)	1.2246	0.29235	0.065826	1.1816	Biological Process: lipid metabolic process (GO:0006629);; Molecular Function: phosphoric diester hydrolase activity (GO:0008081);;	PLCXc domain-containing protein OS=Graphocephala atropunctata OX=36148 GN=g.46366 PE=4 SV=1	uncharacterized protein LOC116340289 [Contarinia nasturtii]
Gene|96::DN10010_c0_g1::g.96::m.96|DN10010_c0_g1:103-1857(-)	1.5061	0.59086	0.014476	1.8393	Molecular Function: hydrolase activity (GO:0016787);;	Carboxylic ester hydrolase OS=Clastoptera arizonana OX=38151 GN=g.8428 PE=3 SV=1	carboxylesterase E2 [Subpsaltria yangi]
Gene|119663::DN86362_c0_g1::g.119663::m.119663|DN86362_c0_g1:95-1357(+)	1.3209	0.4015	0.086904	1061.0	Biological Process: melanin metabolic process (GO:0006582);; Biological Process: cellular aromatic compound metabolic process (GO:0006725);; Biological Process: multicellular organism development (GO:0007275);; Biological Process: biological_process (GO:0008150);; Biological Process:	Uncharacterized protein OS=Clastoptera arizonana OX=38151 GN=g.8539 PE=3 SV=1	protein yellow [Cimex lectularius]

					metabolic process (GO:0008152);; Biological Process: biosynthetic process (GO:0009058);; Biological Process: cellular process (GO:0009987);; Biological Process: phenol-containing compound metabolic process (GO:0018958);; Biological Process: aromatic compound biosynthetic process (GO:0019438);; Biological Process: secondary metabolic process (GO:0019748);; Biological Process: multicellular organismal process (GO:0032501);; Biological Process: developmental process (GO:0032502);; Biological Process: cuticle development (GO:0042335);; Biological Process: melanin biosynthetic process (GO:0042438);; Biological Process: pigment metabolic process (GO:0042440);; Biological Process: pigmentation (GO:0043473);; Biological Process: cellular metabolic process (GO:0044237);; Biological Process: cellular biosynthetic process (GO:0044249);; Biological Process: secondary metabolite biosynthetic process (GO:0044550);; Biological Process: pigment biosynthetic process (GO:0046148);; Biological Process: phenol-containing compound biosynthetic process (GO:0046189);; Biological Process: developmental pigmentation (GO:0048066);; Biological Process: cuticle pigmentation (GO:0048067);; Biological Process: anatomical structure development (GO:0048856);; Biological Process: organic substance metabolic process (GO:0071704);; Biological Process: organic cyclic compound metabolic process (GO:1901360);; Biological Process: organic cyclic compound biosynthetic process (GO:1901362);; Biological Process: organic substance biosynthetic process (GO:1901576);; Biological Process: organic hydroxy compound metabolic process (GO:1901615);; Biological Process: organic hydroxy compound biosynthetic process (GO:1901617);;		
Gene|63749::DN32320_c0_g2::g.63749::m.63749|DN32320_c0_g2:45-671(-)	2.0281	1.0201	0.068143	1.1666	--	--	--
Gene|29705::DN1523_c0_g1::g.29705::m.29705|DN1523_c0_g1:3-329(-)	1.3688	0.45287	0.012238	1.9123	--	--	--
Gene|15775::DN1266_c0_g1::g.15775::m.15775|DN1266_c0_g1:45-1472(+)	1.5251	0.60894	0.0073528	2.1335	--	Uncharacterized protein (Fragment) OS=Clastoptera arizonana OX=38151 GN=g.41232 PE=4 SV=1	--
Gene|30648::DN1541_c0_g1::g.30648::m.30648|DN1541_c0_g1:2-253(+)	1.7771	0.82955	0.075124	1.1242	--	--	--
Gene|91264::DN5351_c1_g2::g.91264::m.91264|DN5351_c1_g2:3-857(+)	1783.0	0.83433	0.022862	1.6409	--	--	--
Gene|11439::DN11924_c0_g1::g.11439::m.11439|DN11924_c0_g1:83-487(+)	1.8526	0.88956	0.0053537	2.2713	--	Uncharacterized protein OS=Clastoptera arizonana OX=38151 GN=g.4397 PE=4 SV=1	probable salivary secreted peptide [Zootermopsis nevadensis]

Supplementary Table S3. Differentially abundant (up-regulated) proteins identified in the foam produced by *Mahanarva spectabilis* in BRI (*Urochloa brizantha* cv. Marandu) versus DEC (*Urochloa decumbens* cv Basilisk), including fold change (FC) values, statistical significance, GO and eggNOG functional annotations, and nr description.

Gene	FC	log2(FC)	raw.pval	-log10(p)	GO_annotation	eggNOG_class_annotation	nr_annotation
Gene|103088::DN6538_c0_g1::g.103088::m.103088|DN6538_c0_g1:68-2533(-)	2.0873	1.0616	0.00039811	3.4	--	Function unknown	inter-alpha-trypsin inhibitor heavy chain H4-like isoform X2 [Rhopalosiphum maidis]
Gene|11151::DN118823_c0_g1::g.11151::m.11151|DN118823_c0_g1:3-311(+)	2.2494	1.1695	0.09648	1.0156	--	--	--
Gene|75477::DN3_c1_g1::g.75477::m.75477|DN3_c1_g1:100-525(-)	2.375	1.2479	2.7593e-06	5.5592	--	--	--
Gene|113908::DN782_c0_g1::g.113908::m.113908|DN782_c0_g1:278-2080(+)	2.7509	1.4599	1.0544e-05	4977.0	Biological Process: intracellular signal transduction (GO:0035556);; Molecular Function: metal ion binding (GO:0046872);;	Signal transduction mechanisms	rac GTPase-activating protein 1 [Megalopta genalis]
Gene|49972::DN24104_c1_g2::g.49972::m.49972|DN24104_c1_g2:1-699(-)	3.8467	1.9436	2.168e-06	5.6639	Molecular Function: ATP binding (GO:0005524);; Cellular Component: cytoplasm (GO:0005737);; Cellular Component: cytoskeleton (GO:0005856);;	Cytoskeleton	actin, muscle [Onthophagus taurus]
Gene|48825::DN2360_c1_g1::g.48825::m.48825|DN2360_c1_g1:1-1716(+)	7.6945	2.9438	2.4002e-06	5.6198	Molecular Function: ATP binding (GO:0005524);; Biological Process: ATP synthesis coupled proton transport (GO:0015986);; Cellular Component: proton-transporting ATP synthase complex, catalytic core F(1) (GO:0045261);; Molecular Function: proton-transporting ATP synthase activity, rotational mechanism (GO:0046933);;	Energy production and conversion	ATP synthase subunit alpha, mitochondrial [Cryptotermes secundus]
Gene|49975::DN24104_c2_g1::g.49975::m.49975|DN24104_c2_g1:139-1269(+)	8.029	3.0052	1.559e-08	7.8071	Molecular Function: ATP binding (GO:0005524);; Cellular Component: cytoplasm (GO:0005737);; Cellular Component: cytoskeleton (GO:0005856);;	Cytoskeleton	actin related protein 1 [Acyrthosiphon pisum]
Gene|29652::DN152210_c0_g1::g.29652::m.29652|DN152210_c0_g1:28-282(-)	1.1479	0.19901	0.078687	1.1041	Molecular Function: structural constituent of ribosome (GO:0003735);; Cellular Component: ribosome (GO:0005840);; Biological Process: translation (GO:0006412);;	--	hypothetical protein FQR65_LT20260 [Abscondita terminalis]
Gene|124738::DN93_c0_g2::g.124738::m.124738|DN93_c0_g2:118-1986(-)	1.1644	0.21954	0.043698	1.3595	Cellular Component: extracellular region (GO:0005576);; Molecular Function: chitin binding (GO:0008061);;	--	hemocytin isoform X1 [Photinus pyralis]
Gene|91261::DN5351_c1_g1::g.91261::m.91261|DN5351_c1_g1:1-1473(-)	1.1671	0.22298	0.090872	1.0416	--	--	--
Gene|99484::DN6182_c1_g1::g.99484::m.99484|DN6182_c1_g1:1030-2628(-)	1.2317	0.3006	0.010486	1.9794	--	--	--
Gene|59666::DN2988_c0_g1::g.59666::m.59666|DN2988_c0_g1:1-540(+)	1.3016	0.38033	0.049148	1.3085	--	--	--
Gene|115205::DN79_c0_g1::g.115205::m.115205|DN79_c0_g1:285-5393(+)	1.3064	0.38563	0.097576	1.0107	--	--	--
Gene|37280::DN1828_c0_g1::g.37280::m.37280|DN1828_c0_g1:103-1872(+)	1.3231	0.40388	0.026421	1.5781	Molecular Function: molecular_function (GO:0003674);; Molecular Function: binding (GO:0005488);; Molecular Function: protein binding (GO:0005515);;	Signal transduction mechanisms	Protease inhibitor I8 domain containing protein [Haemonchus contortus]
Gene|34859::DN17269_c0_g1::g.34859::m.34859|DN17269_c0_g1:1-594(+)	1.3231	0.41904	0.0086591	2.0625	--	--	--
Gene|80872::DN443_c4_g1::g.80872::m.80872|DN443_c4_g1:133-1854(-)	1.4392	0.51402	0.0028652	2.5428	Cellular Component: extracellular region (GO:0005576);; Molecular Function: chitin binding (GO:0008061);;	--	hemocytin isoform X1 [Photinus pyralis]
Gene|72613::DN381_c1_g1::g.72613::m.72613|DN381_c1_g1:1-864(+)	1.4392	0.52528	0.00059668	3.2243	--	Function unknown	gram-negative bacteria binding protein 1 [Plautia stali]
Gene|68184::DN3505_c0_g1::g.68184::m.68184|DN3505_c0_g1:55-777(+)	1.4444	0.53051	0.00011794	3.9283	--	--	--
Gene|27604::DN148298_c1_g1::g.27604::m.27604|DN148298_c1_g1:168-1202(-)	1.4553	0.54133	0.0076475	2.1165	Biological Process: lipid metabolic process (GO:0006629);; Molecular Function: phosphoric diester hydrolase activity (GO:0008081);;	Function unknown	uncharacterized protein LOC116340289 [Contarinia nasturtii]
Gene|26346::DN146260_c12_g1::g.26346::m.26346|DN146260_c12_g1:53-496(-)	1.4876	0.57297	0.022514	1.6476	Cellular Component: nucleosome (GO:0000786);; Molecular Function: DNA binding (GO:0003677);; Cellular Component: nucleus (GO:0005634);; Molecular Function: protein heterodimerization activity (GO:0046982);;	Chromatin structure and dynamics	histone H3 [Armadillidium vulgare]
Gene|93867::DN55_c0_g1::g.93867::m.93867|DN55_c0_g1:122-532(+)	1.4876	0.57297	0.022514	1.6476	Cellular Component: nucleosome (GO:0000786);; Molecular Function: DNA binding (GO:0003677);; Cellular Component: nucleus (GO:0005634);; Molecular Function: protein heterodimerization activity (GO:0046982);;	Chromatin structure and dynamics	core histone H2A/H2B/H3/H4

							[Dictyocaulus viviparus]
Gene|111572::DN7532_c0_g1::g.111572::m.111572|DN7532_c0_g1:105-491(+)	1.5698	0.65062	0.00027931	3.5539	Cellular Component: nucleosome (GO:0000786);; Molecular Function: DNA binding (GO:0003677);; Cellular Component: nucleus (GO:0005634);; Molecular Function: protein heterodimerization activity (GO:0046982);;	Chromatin structure and dynamics	Histone H2A.V, partial [Ooceraea biroi]
Gene|29116::DN151412_c7_g1::g.29116::m.29116|DN151412_c7_g1:3-425(+)	1.5767	0.65692	0.00027684	3.5578	Cellular Component: nucleosome (GO:0000786);; Molecular Function: DNA binding (GO:0003677);;	Chromatin structure and dynamics	histone H2A-like [Aedes aegypti]
Gene|109233::DN728_c2_g1::g.109233::m.109233|DN728_c2_g1:54-425(+)	1.6509	0.72324	0.00047795	3.3206	Cellular Component: nucleosome (GO:0000786);; Molecular Function: DNA binding (GO:0003677);; Cellular Component: nucleus (GO:0005634);; Molecular Function: protein heterodimerization activity (GO:0046982);;	Chromatin structure and dynamics	histone H2B-like [Diachasma alloeum]
Gene|105688::DN6854_c0_g1::g.105688::m.105688|DN6854_c0_g1:124-1113(-)	1.7752	0.82795	0.0057483	2.2405	Molecular Function: triglyceride lipase activity (GO:0004806);; Cellular Component: extracellular region (GO:0005576);;	Posttranslational modification, protein turnover, chaperones	pancreatic triacylglycerol lipase isoform X2 [Orussus abietinus]
Gene|49970::DN24104_c0_g2::g.49970::m.49970|DN24104_c0_g2:2-313(+)	1.8483	0.88617	0.054609	1.2627	Molecular Function: ATP binding (GO:0005524);; Cellular Component: cytoplasm (GO:0005737);; Cellular Component: cytoskeleton (GO:0005856);;	Cytoskeleton	actin-4 [Bombyx mori]
Gene|47672::DN2296_c0_g1::g.47672::m.47672|DN2296_c0_g1:92-850(-)	1.8648	0.89901	7.5841e-06	5.1201	--	--	--
Gene|59599::DN2982_c0_g1::g.59599::m.59599|DN2982_c0_g1:2-370(-)	1.9063	0.93076	6.8082e-05	4167.0	Molecular Function: hydrolase activity, hydrolyzing O-glycosyl compounds (GO:0004553);; Biological Process: carbohydrate metabolic process (GO:0005975);;	--	uncharacterized protein LOC106688707 [Halyomorpha halys]
Gene|61710::DN30_c0_g1::g.61710::m.61710|DN30_c0_g1:353-1483(+)	1.9658	0.97514	0.04244	1.3722	Molecular Function: ATP binding (GO:0005524);; Cellular Component: cytoplasm (GO:0005737);; Cellular Component: cytoskeleton (GO:0005856);;	Cytoskeleton	hypothetical protein DOY81_000649, partial [Sarcophaga bullata]
Gene|1053::DN1015_c0_g1::g.1053::m.1053|DN1015_c0_g1:3-368(-)	1.9925	0.99456	2.2284e-05	4652.0	Molecular Function: hydrolase activity, hydrolyzing O-glycosyl compounds (GO:0004553);; Biological Process: carbohydrate metabolic process (GO:0005975);;	--	uncharacterized protein LOC106662269 isoform X2 [Cimex lectularius]

Supplementary Table S4. Differentially reduced (down-regulated) proteins identified in the foam produced by *Mahanarva spectabilis* in PIO (elephant grass cv. Pioneiro) versus ROXO (elephant grass cv. Roxo de Botucatu), including fold change (FC) values, statistical significance, GO functional annotations, and TrEMBL and nr descriptions.

Gene	FC	log2(FC)	raw.pval	-log10(p)	GO_annotation	TrEMBL_annotation	nr_annotation
Gene|103088::DN6538_c0_g1::g.103088::m.103088|DN6538_c0_g1:68-2533(-)	0.15521	-2.6877	3.7379e-07	6.4274	--	Uncharacterized protein OS=Graphocephala atropunctata OX=36148 GN=g.13618 PE=4 SV=1	inter-alpha-trypsin inhibitor heavy chain H4-like isoform X2 [Rhopalosiphum maidis]
Gene|16116::DN1274_c1_g3::g.16116::m.16116|DN1274_c1_g3:2-1642(+)	0.16091	-2.6356	0.027606	1559	Molecular Function: hydrolase activity, hydrolyzing O-glycosyl compounds (GO:0004553);; Biological Process: carbohydrate metabolic process (GO:0005975);;	Uncharacterized protein OS=Homalodisca liturata OX=320908 GN=g.34826 PE=4 SV=1	hypothetical protein BOX15_Mlig000253g6, partial [Macrostomum lignano]
Gene|32429::DN1614_c0_g1::g.32429::m.32429|DN1614_c0_g1:3-347(+)	0.18045	-2.4703	0.068344	1.1653			
Gene|34859::DN17269_c0_g1::g.34859::m.34859|DN17269_c0_g1:1-594(+)	0.184	-2.4422	0.0013608	2.8662	--	Uncharacterized protein OS=Clastoptera arizonana OX=38151 GN=g.10101 PE=4 SV=1	--
Gene|17926::DN1299_c0_g1::g.17926::m.17926|DN1299_c0_g1:1726-3573(-)	0.20785	-2.2664	0.058178	1.2352	Cellular Component: extracellular space (GO:0005615);;	SERPIN domain-containing protein (Fragment) OS=Clastoptera arizonana OX=38151 GN=g.35749 PE=3 SV=1	secreted Serpin protein [Pristhesancus plagipennis]
Gene|75477::DN3_c1_g1::g.75477::m.75477|DN3_c1_g1:100-525(-)	0.24257	-2.0435	1.7351e-05	4.7607			
Gene|59666::DN2988_c0_g1::g.59666::m.59666|DN2988_c0_g1:1-540(+)	0.24755	-2.0142	0.0043124	2.3653			
Gene|27604::DN148298_c1_g1::g.27604::m.27604|DN148298_c1_g1:168-1202(-)	0.26037	-1.9414	0.0032017	2.4946	Biological Process: lipid metabolic process (GO:0006629);; Molecular Function: phosphoric diester hydrolase activity (GO:0008081);;	PLCXc domain-containing protein OS=Graphocephala atropunctata OX=36148 GN=g.46366 PE=4 SV=1	uncharacterized protein LOC116340289 [Contarinia nasturtii]
Gene|18854::DN1311_c0_g1::g.18854::m.18854|DN1311_c0_g1:1-330(+)	0.27969	-1.8381	1.3099e-05	4.8828			
Gene|105688::DN6854_c0_g1::g.105688::m.105688|DN6854_c0_g1:124-1113(-)	0.28038	-1.8345	4.0754e-07	6.3898	Molecular Function: triglyceride lipase activity (GO:0004806);; Cellular Component: extracellular region (GO:0005576);;	Lipase domain-containing protein OS=Clastoptera arizonana OX=38151 GN=g.5203 PE=3 SV=1	pancreatic triacylglycerol lipase isoform X2 [Orussus abietinus]
Gene|87793::DN5050_c0_g2::g.87793::m.87793|DN5050_c0_g2:2-1132(+)	0.28357	-1.8182	0.042771	1.3688	--	Uncharacterized protein (Fragment) OS=Clastoptera arizonana OX=38151 GN=g.31411 PE=4 SV=1	uncharacterized protein LOC106662269 isoform X1 [Cimex lectularius]
Gene|90054::DN5257_c0_g1::g.90054::m.90054|DN5257_c0_g1:1-2115(+)	0.31091	-1.6854	0.014729	1.8318	Molecular Function: hydrolase activity, hydrolyzing O-glycosyl compounds (GO:0004553);; Biological Process: carbohydrate metabolic process (GO:0005975);;	Uncharacterized protein OS=Laodelphax striatellus OX=195883 GN=LSTR_LSTR011620 PE=4 SV=1	uncharacterized protein LOC111050988 [Nilaparvata lugens]
Gene|113908::DN782_c0_g1::g.113908::m.113908|DN782_c0_g1:278-2080(+)	0.31481	-1.6674	0.0090827	2.0418	Biological Process: intracellular signal transduction (GO:0035556);; Molecular Function: metal ion binding (GO:0046872);;	Uncharacterized protein OS=Clastoptera arizonana OX=38151 GN=g.42869 PE=4 SV=1	rac GTPase-activating protein 1 [Megalopta genalis]
Gene|63545::DN321_c0_g1::g.63545::m.63545|DN321_c0_g1:2-217(+)	0.3187	-1.6497	0.03416	1.4665			
Gene|68184::DN3505_c0_g1::g.68184::m.68184|DN3505_c0_g1:55-777(+)	0.32317	-1.6296	0.073588	1.1332			
Gene|23440::DN13974_c0_g1::g.23440::m.23440|DN13974_c0_g1:3-1604(-)	0.47996	-1.5729	3.396e-05	4469	Molecular Function: hydrolase activity, hydrolyzing O-glycosyl compounds (GO:0004553);; Biological Process: carbohydrate metabolic process (GO:0005975);;	Uncharacterized protein OS=Homalodisca liturata OX=320908 GN=g.34826 PE=4 SV=1	uncharacterized protein LOC111129223 [Crassostrea virginica]
Gene|120316::DN8712_c0_g1::g.120316::m.120316|DN8712_c0_g1:2-1453(+)	0.34245	-1.5729	0.044625	1.3504	--	Uncharacterized protein OS=Clastoptera arizonana OX=38151 GN=g.32872 PE=4 SV=1	--
Gene|87287::DN49_c0_g1::g.87287::m.87287|DN49_c0_g1:303-857(-)	0.33614	-1.5729	0.059045	1.2288	Molecular Function: superoxide dismutase activity (GO:0004784);; Biological Process: superoxide metabolic process (GO:0006801);; Molecular Function: metal ion binding (GO:0046872);;	Sod_Cu domain-containing protein (Fragment) OS=Clastoptera arizonana	Superoxide dismutase [Cu-Zn] [Araneus ventricosus]

						OX=38151 GN=g.3125 PE=4 SV=1	
Gene|123335::DN9149_c2_g1::g.123335::m.123335|DN9149_c2_g1:348-920(-)	0.33629	-1.5722	0.0093219	2.0305	Cellular Component: extracellular region (GO:0005576);; Molecular Function: chitin binding (GO:0008061);; Cellular Component: integral component of membrane (GO:0016021);;	Uncharacterized protein (Fragment) OS=Clastoptera arizonana OX=38151 GN=g.41880 PE=3 SV=1	hypothetical protein LSTR_LSTR001930 [Laodelphax striatellus]
Gene|124742::DN93_c1_g1::g.124742::m.124742|DN93_c1_g1:3-1805(-)	0.36615	-1.4495	0.00087666	3.0572	Molecular Function: hydrolase activity, hydrolyzing O-glycosyl compounds (GO:0004553);; Biological Process: carbohydrate metabolic process (GO:0005975);;	Uncharacterized protein OS=Homalodisca liturata OX=320908 GN=g.34826 PE=4 SV=1	unnamed protein product [Mytilus coruscus]
Gene|8964::DN115271_c1_g1::g.8964::m.8964|DN115271_c1_g1:9-455(+)	0.38004	-1.3958	0.00022482	3.6482			
Gene|1053::DN1015_c0_g1::g.1053::m.1053|DN1015_c0_g1:3-368(-)	0.38384	-1.3814	0.015727	1.8033	Molecular Function: hydrolase activity, hydrolyzing O-glycosyl compounds (GO:0004553);; Biological Process: carbohydrate metabolic process (GO:0005975);;	Uncharacterized protein (Fragment) OS=Clastoptera arizonana OX=38151 GN=g.31402 PE=4 SV=1	uncharacterized protein LOC106662269 isoform X2 [Cimex lectularius]
Gene|8967::DN115271_c2_g1::g.8967::m.8967|DN115271_c2_g1:2-295(+)	0.38661	-1.3711	13	1886			
Gene|100642::DN628_c0_g1::g.100642::m.100642|DN628_c0_g1:2-310(-)	0.42687	-1.2281	0.039262	1406			
Gene|91256::DN5351_c0_g1::g.91256::m.91256|DN5351_c0_g1:1-2286(+)	0.81159	-0.30118	0.02614	1.5827			
Gene|123673::DN92162_c1_g1::g.123673::m.123673|DN92162_c1_g1:133-1203(-)	0.78452	-0.35012	0.012043	1.9193	Biological Process: lipid metabolic process (GO:0006629);; Molecular Function: phosphoric diester hydrolase activity (GO:0008081);;	PLCXc domain-containing protein OS=Graphocephala atropunctata OX=36148 GN=g.46366 PE=4 SV=1	uncharacterized protein LOC116340289 [Contarinia nasturtii]
Gene|122583::DN90435_c0_g1::g.122583::m.122583|DN90435_c0_g1:123-1100(+)	0.75613	-0.40329	0.059623	1.2246	Molecular Function: serine-type endopeptidase activity (GO:0004252);; Biological Process: proteolysis (GO:0006508);;	Peptidase S1 domain-containing protein OS=Clastoptera arizonana OX=38151 GN=g.9423 PE=4 SV=1	PREDICTED: brachyurin-like [Musca domestica]
Gene|101940::DN64294_c0_g1::g.101940::m.101940|DN64294_c0_g1:2-292(-)	1.4703	0.5561	0.065902	1.1811	Biological Process: lipid metabolic process (GO:0006629);; Molecular Function: phosphoric diester hydrolase activity (GO:0008081);;	Uncharacterized protein (Fragment) OS=Homalodisca liturata OX=320908 GN=g.5341 PE=4 SV=1	uncharacterized protein LOC116340289 [Contarinia nasturtii]
Gene|105156::DN677_c0_g1::g.105156::m.105156|DN677_c0_g1:73-2916(+)	0.69339	-0.52827	0.00055331	3257	--	Uncharacterized protein (Fragment) OS=Clastoptera arizonana OX=38151 GN=g.8020 PE=4 SV=1	--
Gene|25728::DN144938_c0_g1::g.25728::m.25728|DN144938_c0_g1:2-286(+)	0.6766	-0.56362	0.011332	1.9457	Molecular Function: protein binding (GO:0005515);;	--	--
Gene|80872::DN443_c4_g1::g.80872::m.80872|DN443_c4_g1:133-1854(-)	0.65221	-0.6166	0.0081234	2.0903	Cellular Component: extracellular region (GO:0005576);; Molecular Function: chitin binding (GO:0008061);;	Uncharacterized protein OS=Photinus pyralis OX=7054 GN=PPYR_11652 PE=3 SV=1	hemocytin isoform X1 [Photinus pyralis]
Gene|49370::DN237_c0_g1::g.49370::m.49370|DN237_c0_g1:1-1101(-)	0.64345	-0.6361	0.081221	1.0903	--	Uncharacterized protein (Fragment) OS=Clastoptera arizonana OX=38151 GN=g.41232 PE=4 SV=1	--
Gene|124738::DN93_c0_g2::g.124738::m.124738|DN93_c0_g2:118-1986(-)	0.63941	-0.64518	0.0068206	2.1662	Cellular Component: extracellular region (GO:0005576);; Molecular Function: chitin binding (GO:0008061);;	Uncharacterized protein OS=Photinus pyralis OX=7054 GN=PPYR_11652 PE=3 SV=1	hemocytin isoform X1 [Photinus pyralis]
Gene|65412::DN334_c1_g1::g.65412::m.65412|DN334_c1_g1:1-282(-)	0.6369	-0.65086	0.033916	1.4696			
Gene|16112::DN1274_c0_g1::g.16112::m.16112|DN1274_c0_g1:1-1236(+)	0.53112	-0.91289	0.0099731	2.0012	--	Uncharacterized protein (Fragment) OS=Clastoptera arizonana OX=38151 GN=g.31411 PE=4 SV=1	uncharacterized protein LOC111129223 [Crassostrea virginica]
Gene|99484::DN6182_c1_g1::g.99484::m.99484|DN6182_c1_g1:1030-2628(-)	0.52019	-0.9429	0.0088331	2.0539			
Gene|105416::DN680_c0_g1::g.105416::m.105416|DN680_c0_g1:115-1419(-)	0.55019	-0.86199	0.032853	1.4834			
Gene|89557::DN52028_c0_g1::g.89557::m.89557|DN52028_c0_g1:3-248(-)	0.54718	-0.8699	0.043939	1.3572			

Supplementary Table S5. Differentially reduced (down-regulated) proteins identified in the foam produced by *Mahanarva spectabilis* in BRI (*Urochloa brizantha* cv. Marandu) versus DEC (*Urochloa decumbens* cv Basilisk), including fold change (FC) values, statistical significance, GO and eggNOG functional annotations, and nr description.

Gene	FC	log2(FC)	raw.pval	-log10(p)	GO_annotation	eggNOG_class_annotation	nr_annotation
Gene|105031::DN675_c1_g1::g.105031::m.105031|DN675_c1_g1:3-431(-)	0.12688	-2.9784	0.0071565	2.1453	--	--	--
Gene|39434::DN19253_c0_g1::g.39434::m.39434|DN19253_c0_g1:44-1282(+)	0.17395	-2.5232	0.0038665	2.4127	Biological Process: melanin metabolic process (GO:0006582);; Biological Process: cellular aromatic compound metabolic process (GO:0006725);; Biological Process: multicellular organism development (GO:0007275);; Biological Process: biological_process (GO:0008150);; Biological Process: metabolic process (GO:0008152);; Biological Process: biosynthetic process (GO:0009058);; Biological Process: cellular process (GO:0009987);; Biological Process: phenol-containing compound metabolic process (GO:0018958);; Biological Process: aromatic compound biosynthetic process (GO:0019438);; Biological Process: secondary metabolic process (GO:0019748);; Biological Process: multicellular organismal process (GO:0032501);; Biological Process: developmental process (GO:0032502);; Biological Process: cuticle development (GO:0042335);; Biological Process: melanin biosynthetic process (GO:0042438);; Biological Process: pigment metabolic process (GO:0042440);; Biological Process: pigmentation (GO:0043473);; Biological Process: cellular metabolic process (GO:0044237);; Biological Process: cellular biosynthetic process (GO:0044249);; Biological Process: secondary metabolite biosynthetic process (GO:0044550);; Biological Process: pigment biosynthetic process (GO:0046148);; Biological Process: phenol-containing compound biosynthetic process (GO:0046189);; Biological Process: developmental pigmentation (GO:0048066);; Biological Process: cuticle pigmentation (GO:0048067);; Biological Process: anatomical structure development (GO:0048856);; Biological Process: organic substance metabolic process (GO:0071704);; Biological Process: organic cyclic compound metabolic process (GO:1901360);; Biological Process: organic cyclic compound biosynthetic process (GO:1901362);; Biological Process: organic substance biosynthetic process (GO:1901576);; Biological Process: organic hydroxy compound metabolic process (GO:1901615);; Biological Process: organic hydroxy compound biosynthetic process (GO:1901617);;	Carbohydrate transport and metabolism	hypothetical protein LSTR_LSTR012017 [Laodelphax striatellus]
Gene|11439::DN11924_c0_g1::g.11439::m.11439|DN11924_c0_g1:83-487(+)	0.22686	-2.1401	7.7199e-06	5.1124	--	Function unknown	probable salivary secreted peptide [Zootermopsis nevadensis]
Gene|9596::DN116380_c0_g1::g.9596::m.9596|DN116380_c0_g1:2-3931(+)	0.23256	-2.1043	5.7236e-12	11242	--	--	--
Gene|47299::DN22748_c0_g1::g.47299::m.47299|DN22748_c0_g1:78-1163(+)	0.3011	-1.7317	0.053674	1.2702	Biological Process: pseudouridine synthesis (GO:0001522);; Molecular Function: RNA binding (GO:0003723);; Molecular Function: pseudouridine synthase activity (GO:0009982);;	RNA processing and modification	mitochondrial RNA pseudouridine synthase rpusd4-like [Cimex lectularius]
Gene|90054::DN5257_c0_g1::g.90054::m.90054|DN5257_c0_g1:1-2115(+)	0.32667	-1.6141	0.058902	1.2299	Molecular Function: hydrolase activity, hydrolyzing O-glycosyl compounds (GO:0004553);; Biological Process: carbohydrate metabolic process (GO:0005975);;	--	uncharacterized protein LOC111050988 [Nilaparvata lugens]
Gene|30185::DN153313_c0_g1::g.30185::m.30185|DN153313_c0_g1:173-778(-)	0.36636	-1.4487	0.0016617	2.7794	--	--	--
Gene|939::DN101344_c0_g1::g.939::m.939|DN101344_c0_g1:3-548(-)	0.37229	-1.4255	0.0013919	2.8564	Biological Process: lipid metabolic process (GO:0006629);; Molecular Function: phosphoric diester hydrolase activity (GO:0008081);;	Function unknown	uncharacterized protein LOC116340289 [Contarinia nasturtii]
Gene|17025::DN128683_c0_g2::g.17025::m.17025|DN128683_c0_g2:518-2014(-)	0.37907	-1.3994	4.164e-05	4.3805	--	--	--
Gene|43644::DN21145_c0_g1::g.43644::m.43644|DN21145_c0_g1:171-1568(+)	0.39582	-1.3371	0.00091608	3.0381	Molecular Function: serine-type carboxypeptidase activity (GO:0004185);;	Posttranslational modification, protein turnover, chaperones	venom serine carboxypeptidase [Cryptotermes secundus]
Gene|68129::DN35000_c0_g1::g.68129::m.68129|DN35000_c0_g1:36-683(+)	0.39662	-1.3342	0.0056064	2.2513	--	--	--
Gene|2656::DN10399_c0_g2::g.2656::m.2656|DN10399_c0_g2:3-971(-)	0.41833	-1.2573	5.4216e-05	4.2659	--	--	--

Gene|119663::DN86362_c0_g1::g.119663::m.119663|DN86362_c0_g1:95-1357(+)	0.41891	-1.2553	2.1494e-08	7.6677	Biological Process: melanin metabolic process (GO:0006582);; Biological Process: cellular aromatic compound metabolic process (GO:0006725);; Biological Process: multicellular organism development (GO:0007275);; Biological Process: biological_process (GO:0008150);; Biological Process: metabolic process (GO:0008152);; Biological Process: biosynthetic process (GO:0009058);; Biological Process: cellular process (GO:0009987);; Biological Process: phenol-containing compound metabolic process (GO:0018958);; Biological Process: aromatic compound biosynthetic process (GO:0019438);; Biological Process: secondary metabolic process (GO:0019748);; Biological Process: multicellular organismal process (GO:0032501);; Biological Process: developmental process (GO:0032502);; Biological Process: cuticle development (GO:0042335);; Biological Process: melanin biosynthetic process (GO:0042438);; Biological Process: pigment metabolic process (GO:0042440);; Biological Process: pigmentation (GO:0043473);; Biological Process: cellular metabolic process (GO:0044237);; Biological Process: cellular biosynthetic process (GO:0044249);; Biological Process: secondary metabolite biosynthetic process (GO:0044550);; Biological Process: pigment biosynthetic process (GO:0046148);; Biological Process: phenol-containing compound biosynthetic process (GO:0046189);; Biological Process: developmental pigmentation (GO:0048066);; Biological Process: cuticle pigmentation (GO:0048067);; Biological Process: anatomical structure development (GO:0048856);; Biological Process: organic substance metabolic process (GO:0071704);; Biological Process: organic cyclic compound metabolic process (GO:1901360);; Biological Process: organic cyclic compound biosynthetic process (GO:1901362);; Biological Process: organic substance biosynthetic process (GO:1901576);; Biological Process: organic hydroxy compound metabolic process (GO:1901615);; Biological Process: organic hydroxy compound biosynthetic process (GO:1901617);;	Carbohydrate transport and metabolism	protein yellow [Cimex lectularius]
Gene|28211::DN149759_c0_g1::g.28211::m.28211|DN149759_c0_g1:56-760(-)	0.43512	-1.2005	6.3447e-06	5.1976	--	--	Apolipoprotein D [Eumeta japonica]
Gene|9962::DN116_c0_g1::g.9962::m.9962|DN116_c0_g1:695-2047(-)	0.43985	-1.1849	0.0091213	2.0399	Molecular Function: GTPase activity (GO:0003924);; Molecular Function: structural constituent of cytoskeleton (GO:0005200);; Molecular Function: GTP binding (GO:0005525);; Cellular Component: cytoplasm (GO:0005737);; Cellular Component: microtubule (GO:0005874);; Biological Process: microtubule-based process (GO:0007017);;	Cytoskeleton	tubulin [Schistocerca americana]
Gene|125314::DN94_c0_g1::g.125314::m.125314|DN94_c0_g1:125-1477(+)	0.43985	-1.1849	0.0091213	2.0399	Molecular Function: GTPase activity (GO:0003924);; Molecular Function: structural constituent of cytoskeleton (GO:0005200);; Molecular Function: GTP binding (GO:0005525);; Cellular Component: cytoplasm (GO:0005737);; Cellular Component: microtubule (GO:0005874);; Biological Process: microtubule-based process (GO:0007017);;	Cytoskeleton	tubulin alpha-1 chain [Nilaparvata lugens]
Gene|125317::DN94_c1_g1::g.125317::m.125317|DN94_c1_g1:1-1149(-)	0.43985	-1.1849	0.0091213	2.0399	Molecular Function: GTPase activity (GO:0003924);; Molecular Function: structural constituent of cytoskeleton (GO:0005200);; Molecular Function: GTP binding (GO:0005525);; Cellular Component: microtubule (GO:0005874);; Biological Process: microtubule-based process (GO:0007017);;	Cytoskeleton	PREDICTED: tubulin alpha-1C chain [Diuraphis noxia]
Gene|44311::DN2142_c0_g1::g.44311::m.44311|DN2142_c0_g1:3-269(+)	0.47754	-1.0663	0.0055053	2.2592	--	--	--
Gene|86624::DN49332_c0_g1::g.86624::m.86624|DN49332_c0_g1:195-2168(-)	0.47502	-1.0585	3.6758e-05	4.4347	Molecular Function: copper ion binding (GO:0005507);; Molecular Function: oxidoreductase activity (GO:0016491);; Biological Process: oxidation-reduction process (GO:0055114);;	Secondary metabolites biosynthesis, transport and catabolism	multicopper oxidase [Nephotettix cincticeps]
Gene|69767::DN36093_c0_g1::g.69767::m.69767|DN36093_c0_g1:2-2098(+)	0.48012	-1.0585	0.00018357	3.7362	--	--	--
Gene|29301::DN151595_c0_g1::g.29301::m.29301|DN151595_c0_g1:3-1367(+)	0.4922	-1.0227	0.04724	1.3257	--	--	--
Gene|101940::DN64294_c0_g1::g.101940::m.101940|DN64294_c0_g1:2-292(-)	0.49372	-1.0182	0.0022234	2653	Biological Process: lipid metabolic process (GO:0006629);; Molecular Function: phosphoric diester hydrolase activity (GO:0008081);;	--	uncharacterized protein LOC116340289 [Contarinia nasturtii]
Gene|122372::DN8_c0_g1::g.122372::m.122372|DN8_c0_g1:66-1559(-)	0.13726	-2.865	0.010081	1.9965	--	--	--
Gene|34230::DN16908_c0_g1::g.34230::m.34230|DN16908_c0_g1:205-1614(+)	0.36882	-1439	0.018944	1.7225	Molecular Function: hydrolase activity, hydrolyzing O-glycosyl compounds (GO:0004553);; Biological Process: carbohydrate metabolic process (GO:0005975);;	Carbohydrate transport and metabolism	hypothetical protein LSTR_LSTR007242 [Laodelphax striatellus]
Gene|124742::DN93_c1_g1::g.124742::m.124742|DN93_c1_g1:3-1805(-)	0.8776	-0.18836	0.052363	1281	Molecular Function: hydrolase activity, hydrolyzing O-glycosyl compounds (GO:0004553);; Biological Process: carbohydrate metabolic process (GO:0005975);;	--	unnamed protein product [Mytilus coruscus]

Gene|48420::DN2337_c1_g1::g.48420::m.48420|DN2337_c1_g1:2206-4263(+)	0.74283	-0.4289	0.05872	1.2312	--	--	--
Gene|50887::DN2460_c0_g1::g.50887::m.50887|DN2460_c0_g1:168-752(-)	1.3538	0.43705	0.003934	2.4052	Molecular Function: molecular_function (GO:0003674);; Molecular Function: serine-type endopeptidase inhibitor activity (GO:0004867);; Molecular Function: structural molecule activity (GO:0005198);; Molecular Function: extracellular matrix structural constituent (GO:0005201);; Cellular Component: cellular_component (GO:0005575);; Cellular Component: extracellular region (GO:0005576);; Cellular Component: basement membrane (GO:0005604);; Biological Process: biological_process (GO:0008150);; Molecular Function: peptidase activity (GO:0008233);; Biological Process: cellular process (GO:0009987);; Biological Process: cellular component organization (GO:0016043);; Biological Process: extracellular matrix organization (GO:0030198);; Cellular Component: extracellular matrix (GO:0031012);; Biological Process: extracellular structure organization (GO:0043062);; Cellular Component: obsolete extracellular region part (GO:0044421);; Cellular Component: collagen-containing extracellular matrix (GO:0062023);; Biological Process: cellular component organization or biogenesis (GO:0071840);;	Posttranslational modification, protein turnover, chaperones	papilin isoform X1 [Drosophila hydei]
Gene|107961::DN713_c0_g1::g.107961::m.107961|DN713_c0_g1:185-1354(-)	0.73737	-0.43953	0.001745	2.7582	Biological Process: reproduction (GO:0000003);; Molecular Function: molecular_function (GO:0003674);; Molecular Function: enzyme inhibitor activity (GO:0004857);; Molecular Function: endopeptidase inhibitor activity (GO:0004866);; Molecular Function: serine-type endopeptidase inhibitor activity (GO:0004867);; Cellular Component: cellular_component (GO:0005575);; Cellular Component: extracellular region (GO:0005576);; Cellular Component: extracellular space (GO:0005615);; Biological Process: biological_process (GO:0008150);; Biological Process: negative regulation of metabolic process (GO:0009892);; Biological Process: negative regulation of peptidase activity (GO:0010466);; Biological Process: negative regulation of macromolecule metabolic process (GO:0010605);; Biological Process: negative regulation of endopeptidase activity (GO:0010951);; Biological Process: regulation of metabolic process (GO:0019222);; Biological Process: regulation of proteolysis (GO:0030162);; Molecular Function: enzyme regulator activity (GO:0030234);; Molecular Function: peptidase inhibitor activity (GO:0030414);; Biological Process: regulation of cellular metabolic process (GO:0031323);; Biological Process: negative regulation of cellular metabolic process (GO:0031324);; Biological Process: regulation of cellular protein metabolic process (GO:0032268);; Biological Process: negative regulation of cellular protein metabolic process (GO:0032269);; Biological Process: multicellular organismal process (GO:0032501);; Biological Process: multicellular organism reproduction (GO:0032504);; Biological Process: negative regulation of catalytic activity (GO:0043086);; Biological Process: negative regulation of molecular function (GO:0044092);; Cellular Component: obsolete extracellular region part (GO:0044421);; Biological Process: negative regulation of proteolysis (GO:0045861);; Biological Process: negative regulation of biological process (GO:0048519);; Biological Process: negative regulation of cellular process (GO:0048523);; Biological Process: regulation of biological process (GO:0050789);; Biological Process: regulation of catalytic activity (GO:0050790);; Biological Process: regulation of cellular process (GO:0050794);; Biological Process: regulation of nitrogen compound metabolic process (GO:0051171);; Biological Process: negative regulation of nitrogen compound metabolic process (GO:0051172);; Biological Process: regulation of protein metabolic process (GO:0051246);; Biological Process: negative regulation of protein metabolic process (GO:0051248);; Biological Process: regulation of hydrolase activity (GO:0051336);; Biological Process: negative regulation of hydrolase activity (GO:0051346);; Biological Process: regulation of peptidase activity (GO:0052547);; Biological Process: regulation of endopeptidase activity (GO:0052548);; Biological Process: regulation of macromolecule metabolic process (GO:0060255);; Molecular Function: peptidase regulator activity (GO:0061134);; Molecular Function: endopeptidase regulator activity (GO:0061135);; Biological Process: biological regulation (GO:0065007);; Biological Process: regulation of molecular function (GO:0065009);; Biological Process: regulation of primary metabolic process (GO:0080090);; Molecular Function: molecular function regulator (GO:0098772);;	Defense mechanisms	antichymotrypsin-2-like isoform X6 [Diabrotica virgifera virgifera]
Gene|51413::DN249_c0_g1::g.51413::m.51413|DN249_c0_g1:109-3054(-)	0.73449	-0.44519	0.042435	1.3723	--	--	--

Gene|105156::DN677_c0_g1::g.105156::m.105156|DN677_c0_g1:73-2916(+)	1.3636	0.44744	0.011996	1921	--	--	--
Gene|31020::DN155_c0_g1::g.31020::m.31020|DN155_c0_g1:69-344(-)	0.73276	-0.44859	0.023151	1.6354	--	--	--
Gene|96371::DN588_c0_g1::g.96371::m.96371|DN588_c0_g1:2-4906(-)	0.71983	-0.47427	0.01805	1.7435	--	--	--
Gene|49576::DN238_c0_g1::g.49576::m.49576|DN238_c0_g1:93-2378(-)	0.71653	-0.4809	0.0069994	2.1549	--	--	--
Gene|39089::DN19110_c0_g2::g.39089::m.39089|DN19110_c0_g2:3-1280(+)	0.70149	-0.51152	0.097426	1.0113	--	--	--
Gene|103375::DN656_c0_g1::g.103375::m.103375|DN656_c0_g1:1-426(+)	0.85601	-0.2243	0.085334	1.0689	--	--	--
Gene|100644::DN628_c2_g1::g.100644::m.100644|DN628_c2_g1:2-283(-)	0.85601	-0.2243	0.085334	1.0689	--	--	--
Gene|14174::DN1237_c0_g1::g.14174::m.14174|DN1237_c0_g1:78-2423(-)	0.82344	-0.28027	0.0066272	2.1787	--	--	--
Gene|25728::DN144938_c0_g1::g.25728::m.25728|DN144938_c0_g1:2-286(+)	0.81912	-0.28785	0.057678	1239	Molecular Function: protein binding (GO:0005515);;	--	--
Gene|29705::DN1523_c0_g1::g.29705::m.29705|DN1523_c0_g1:3-329(-)	0.80049	-0.32104	0.0083621	2.0777	--	--	--
Gene|55109::DN2699_c0_g1::g.55109::m.55109|DN2699_c0_g1:2-11059(+)	0.79584	-0.32945	0.046632	1.3313	--	--	--
Gene|9657::DN1164_c0_g1::g.9657::m.9657|DN1164_c0_g1:72-1391(+)	0.79233	-0.33582	0.021011	1.6776	--	--	--
Gene|28910::DN1508_c0_g1::g.28910::m.28910|DN1508_c0_g1:102-1517(+)	0.79197	-0.33649	0.020363	1.6912	--	--	--
Gene|41141::DN2003_c0_g1::g.41141::m.41141|DN2003_c0_g1:2070-3482(-)	0.7781	-0.36197	0.055911	1.2525	--	--	--
Gene|60882::DN3043_c0_g1::g.60882::m.60882|DN3043_c0_g1:111-4721(-)	0.76927	-0.37843	0.00025514	3.5932	--	--	--
Gene|25376::DN144491_c0_g1::g.25376::m.25376|DN144491_c0_g1:100-1434(-)	0.75288	-0.4095	0.088675	1.0522	--	--	--
Gene|25773::DN144_c0_g1::g.25773::m.25773|DN144_c0_g1:220-1704(-)	0.75166	-0.41184	0.0010595	2.9749	--	--	--
Gene|44043::DN21301_c0_g1::g.44043::m.44043|DN21301_c0_g1:17-1510(-)	0.69888	-0.51688	0.0011103	2.9546	Molecular Function: hydrolase activity, hydrolyzing O-glycosyl compounds (GO:0004553);; Biological Process: carbohydrate metabolic process (GO:0005975);;	Carbohydrate transport and metabolism	PREDICTED: myrosinase 1 [Bemisia tabaci]
Gene|17926::DN1299_c0_g1::g.17926::m.17926|DN1299_c0_g1:1726-3573(-)	0.69785	-0.51902	0.054442	1.2641	Cellular Component: extracellular space (GO:0005615);;	Defense mechanisms	secreted Serpin protein [Pristhesancus plagipennis]
Gene|106927::DN702_c0_g1::g.106927::m.106927|DN702_c0_g1:258-1313(+)	0.6698	-0.57819	0.0039433	2.4041	Biological Process: lipid metabolic process (GO:0006629);; Molecular Function: phosphoric diester hydrolase activity (GO:0008081);;	Signal transduction mechanisms	uncharacterized protein LOC116340289 [Contarinia nasturtii]
Gene|97354::DN5_c3_g1::g.97354::m.97354|DN5_c3_g1:1-600(-)	0.66179	-0.59555	0.07749	1.1108	Biological Process: cellular amino acid metabolic process (GO:0006520);; Molecular Function: amino acid binding (GO:0016597);; Molecular Function: carboxyl- or carbamoyltransferase activity (GO:0016743);;	--	hypothetical protein FQR65_LT17659 [Abscondita terminalis]
Gene|20855::DN134540_c0_g1::g.20855::m.20855|DN134540_c0_g1:3-248(+)	0.66179	-0.59555	0.07749	1.1108	Biological Process: cellular amino acid metabolic process (GO:0006520);; Molecular Function: amino acid binding (GO:0016597);; Molecular Function: carboxyl- or carbamoyltransferase activity (GO:0016743);;	--	--
Gene|5935::DN1091_c0_g1::g.5935::m.5935|DN1091_c0_g1:2-277(+)	0.66016	-0.59911	0.012609	1.8993	--	--	--
Gene|73191::DN38649_c0_g1::g.73191::m.73191|DN38649_c0_g1:698-2857(-)	0.65483	-0.61081	0.00010545	3977	--	--	--
Gene|9418::DN11605_c0_g1::g.9418::m.9418|DN11605_c0_g1:119-754(+)	0.64438	-0.63401	0.00024772	3606	--	--	apolipoprotein D-like [Eurytemora affinis]
Gene|40922::DN1992_c0_g1::g.40922::m.40922|DN1992_c0_g1:598-5478(-)	0.63619	-0.65248	0.002404	2.6191	--	--	hypothetical protein B7P43_G08554 [Cryptotermes secundus]
Gene|30710::DN15445_c0_g1::g.30710::m.30710|DN15445_c0_g1:3-938(-)	0.63619	-0.65248	0.002404	2.6191	Molecular Function: monooxygenase activity (GO:0004497);; Molecular Function: iron ion binding (GO:0005506);; Cellular Component: integral component of membrane (GO:0016021);; Molecular Function: oxidoreductase activity, acting on paired donors, with incorporation or reduction of molecular oxygen (GO:0016705);; Molecular Function: heme binding (GO:0020037);;	Secondary metabolites biosynthesis, transport and catabolism	cytochrome P450 [Locusta migratoria]
Gene|74414::DN3947_c0_g1::g.74414::m.74414|DN3947_c0_g1:2819-4771(-)	0.63619	-0.65248	0.002404	2.6191	Molecular Function: glutamate-cysteine ligase activity (GO:0004357);; Biological Process: glutathione biosynthetic process (GO:0006750);;	Coenzyme transport and metabolism	glutamate--cysteine ligase catalytic subunit [Zootermopsis nevadensis]
Gene|125379::DN95101_c0_g1::g.125379::m.125379|DN95101_c0_g1:222-6155(+)	0.63619	-0.65248	0.002404	2.6191	Biological Process: mitotic sister chromatid segregation (GO:0000070);; Biological Process: cell cycle checkpoint (GO:0000075);; Biological Process: mitotic cell cycle (GO:0000278);; Biological Process: nuclear division (GO:0000280);; Cellular Component: chromosome, centromeric region (GO:0000775);; Cellular Component: kinetochore (GO:0000776);; Cellular Component: condensed chromosome kinetochore (GO:0000777);; Cellular Component: condensed chromosome, centromeric region (GO:0000779);;	Cell cycle control, cell division, chromosome partitioning	kinetochore-associated protein 1 isoform X1 [Cryptotermes secundus]

Cellular Component: condensed chromosome (GO:0000793);; Biological Process: sister chromatid segregation (GO:0000819);; Cellular Component: condensed chromosome outer kinetochore (GO:0000940);; Molecular Function: molecular_function (GO:0003674);; Molecular Function: binding (GO:0005488);; Molecular Function: protein binding (GO:0005515);; Cellular Component: cellular_component (GO:0005575);; Cellular Component: intracellular (GO:0005622);; Cellular Component: obsolete cell (GO:0005623);; Cellular Component: chromosome (GO:0005694);; Cellular Component: cytoplasm (GO:0005737);; Cellular Component: Golgi apparatus (GO:0005794);; Cellular Component: Golgi stack (GO:0005795);; Cellular Component: spindle (GO:0005819);; Cellular Component: kinetochore microtubule (GO:0005828);; Cellular Component: cytoskeleton (GO:0005856);; Cellular Component: microtubule (GO:0005874);; Cellular Component: spindle microtubule (GO:0005876);; Biological Process: organelle organization (GO:0006996);; Biological Process: Golgi organization (GO:0007030);; Biological Process: cell cycle (GO:0007049);; Biological Process: chromosome segregation (GO:0007059);; Biological Process: regulation of mitotic nuclear division (GO:0007088);; Biological Process: mitotic cell cycle checkpoint (GO:0007093);; Biological Process: mitotic spindle assembly checkpoint (GO:0007094);; Biological Process: regulation of mitotic cell cycle (GO:0007346);; Biological Process: protein localization (GO:0008104);; Biological Process: biological_process (GO:0008150);; Biological Process: cellular process (GO:0009987);; Biological Process: endomembrane system organization (GO:0010256);; Biological Process: regulation of cell cycle process (GO:0010564);; Biological Process: negative regulation of organelle organization (GO:0010639);; Biological Process: negative regulation of cell cycle process (GO:0010948);; Biological Process: regulation of mitotic sister chromatid separation (GO:0010965);; Cellular Component: endomembrane system (GO:0012505);; Cellular Component: microtubule cytoskeleton (GO:0015630);; Biological Process: cellular component organization (GO:0016043);; Molecular Function: Ras GTPase binding (GO:0017016);; Molecular Function: Rab GTPase binding (GO:0017137);; Molecular Function: enzyme binding (GO:0019899);; Biological Process: cell cycle process (GO:0022402);; Biological Process: regulation of mitotic metaphase/anaphase transition (GO:0030071);; Molecular Function: small GTPase binding (GO:0031267);; Biological Process: spindle checkpoint (GO:0031577);; Cellular Component: organelle subcompartment (GO:0031984);; Cellular Component: protein-containing complex (GO:0032991);; Biological Process: macromolecule localization (GO:0033036);; Biological Process: regulation of organelle organization (GO:0033043);; Biological Process: regulation of chromosome organization (GO:0033044);; Biological Process: regulation of sister chromatid segregation (GO:0033045);; Biological Process: negative regulation of sister chromatid segregation (GO:0033046);; Biological Process: regulation of mitotic sister chromatid segregation (GO:0033047);; Biological Process: negative regulation of mitotic sister chromatid segregation (GO:0033048);; Biological Process: protein localization to organelle (GO:0033365);; Biological Process: protein localization to kinetochore (GO:0034501);; Biological Process: protein localization to chromosome (GO:0034502);; Biological Process: cellular protein localization (GO:0034613);; Cellular Component: acroblast (GO:0036063);; Cellular Component: organelle (GO:0043226);; Cellular Component: membrane-bounded organelle (GO:0043227);; Cellular Component: non-membrane-bounded organelle (GO:0043228);; Cellular Component: intracellular organelle (GO:0043229);; Cellular Component: intracellular membrane-bounded organelle (GO:0043231);; Cellular Component: intracellular non-membrane-bounded organelle (GO:0043232);; Cellular Component: obsolete organelle part (GO:0044422);; Cellular Component: obsolete intracellular part (GO:0044424);; Cellular Component: obsolete chromosomal part (GO:0044427);; Cellular Component: obsolete cytoskeletal part (GO:0044430);; Cellular Component: obsolete Golgi apparatus part (GO:0044431);; Cellular Component: obsolete cytoplasmic part (GO:0044444);; Cellular Component: obsolete intracellular organelle part (GO:0044446);; Cellular Component: obsolete cell part (GO:0044464);; Biological Process: negative regulation of cell cycle (GO:0045786);; Biological Process: negative regulation of mitotic nuclear division (GO:0045839);; Biological Process: negative regulation of mitotic

					metaphase/anaphase transition (GO:0045841);; Biological Process: negative regulation of mitotic cell cycle (GO:0045930);; Biological Process: organelle fission (GO:0048285);; Biological Process: negative regulation of biological process (GO:0048519);; Biological Process: negative regulation of cellular process (GO:0048523);; Biological Process: regulation of biological process (GO:0050789);; Biological Process: regulation of cellular process (GO:0050794);; Molecular Function: GTPase binding (GO:0051020);; Biological Process: regulation of cellular component organization (GO:0051128);; Biological Process: negative regulation of cellular component organization (GO:0051129);; Biological Process: localization (GO:0051179);; Cellular Component: spindle midzone (GO:0051233);; Biological Process: chromosome organization (GO:0051276);; Biological Process: cellular localization (GO:0051641);; Biological Process: regulation of cell cycle (GO:0051726);; Biological Process: regulation of nuclear division (GO:0051783);; Biological Process: negative regulation of nuclear division (GO:0051784);; Biological Process: regulation of chromosome segregation (GO:0051983);; Biological Process: negative regulation of chromosome segregation (GO:0051985);; Biological Process: biological regulation (GO:0065007);; Biological Process: cellular macromolecule localization (GO:0070727);; Biological Process: spindle assembly checkpoint (GO:0071173);; Biological Process: mitotic spindle checkpoint (GO:0071174);; Biological Process: protein localization to chromosome, centromeric region (GO:0071459);; Biological Process: cellular component organization or biogenesis (GO:0071840);; Cellular Component: chromosomal region (GO:0098687);; Cellular Component: Golgi apparatus subcompartment (GO:0098791);; Biological Process: nuclear chromosome segregation (GO:0098813);; Cellular Component: supramolecular complex (GO:0099080);; Cellular Component: supramolecular polymer (GO:0099081);; Cellular Component: supramolecular fiber (GO:0099512);; Cellular Component: polymeric cytoskeletal fiber (GO:0099513);; Biological Process: mitotic nuclear division (GO:0140014);; Biological Process: regulation of cell cycle phase transition (GO:1901987);; Biological Process: negative regulation of cell cycle phase transition (GO:1901988);; Biological Process: regulation of mitotic cell cycle phase transition (GO:1901990);; Biological Process: negative regulation of mitotic cell cycle phase transition (GO:1901991);; Biological Process: regulation of metaphase/anaphase transition of cell cycle (GO:1902099);; Biological Process: negative regulation of metaphase/anaphase transition of cell cycle (GO:1902100);; Biological Process: mitotic cell cycle process (GO:1903047);; Biological Process: regulation of chromosome separation (GO:1905818);; Biological Process: negative regulation of chromosome separation (GO:1905819);; Cellular Component: RZZ complex (GO:1990423);; Biological Process: negative regulation of mitotic sister chromatid separation (GO:2000816);; Biological Process: negative regulation of chromosome organization (GO:2001251);;		
Gene|64061::DN3253_c2_g2::g.64061::m.64061|DN3253_c2_g2:310-2376(+)	0.63619	-0.65248	0.002404	2.6191	--	Function unknown	U3 small nucleolar RNA-associated protein 4 homolog [Cryptotermes secundus]
Gene|50914::DN24623_c0_g1::g.50914::m.50914|DN24623_c0_g1:147-1601(-)	0.67448	-0.56816	0.061441	1.2115	--	--	--
Gene|96::DN10010_c0_g1::g.96::m.96|DN10010_c0_g1:103-1857(-)	0.68758	-0.54041	0.00084298	3.0742	Molecular Function: hydrolase activity (GO:0016787);;	Carbohydrate transport and metabolism	carboxylesterase E2 [Subpsaltria yangi]
Gene|122335::DN89953_c0_g2::g.122335::m.122335|DN89953_c0_g2:504-665(-)	0.60496	-0.72509	0.081088	1091	--	--	--
Gene|98956::DN61276_c0_g2::g.98956::m.98956|DN61276_c0_g2:25-1965(+)	0.60206	-0.73202	9.5219e-05	4.0213	Molecular Function: oxidoreductase activity, acting on CH-OH group of donors (GO:0016614);; Molecular Function: flavin adenine dinucleotide binding (GO:0050660);; Biological Process: oxidation-reduction process (GO:0055114);;	Amino acid transport and metabolism	Glucose dehydrogenase [FAD, quinone] [Blattella germanica]
Gene|105416::DN680_c0_g1::g.105416::m.105416|DN680_c0_g1:115-1419(-)	0.58931	-0.76289	1.5013e-07	6.8235	--	--	--
Gene|15775::DN1266_c0_g1::g.15775::m.15775|DN1266_c0_g1:45-1472(+)	0.57889	-0.78865	0.00016753	3.7759	--	--	--
Gene|1606::DN102479_c0_g1::g.1606::m.1606|DN102479_c0_g1:1355-4060(-)	0.62383	-0.68077	0.0063949	2.1942	--	--	--

Gene|94739::DN5701_c0_g1::g.94739::m.94739|DN5701_c0_g1:2-427(+)	0.54486	-0.87605	2.7826e-05	4.5555	--	--	--
Gene|122466::DN90258_c0_g1::g.122466::m.122466|DN90258_c0_g1:50-1468(+)	0.53386	-0.90547	0.0032118	2.4933	--	--	--
Gene|69580::DN3595_c0_g1::g.69580::m.69580|DN3595_c0_g1:160-1338(+)	0.51715	-0.95134	0.042654	1.37	Molecular Function: tumor necrosis factor receptor binding (GO:0005164);; Biological Process: immune response (GO:0006955);; Cellular Component: integral component of membrane (GO:0016021);;	--	uncharacterized protein LOC110828012 isoform X4 [Zootermopsis nevadensis]
Gene|122583::DN90435_c0_g1::g.122583::m.122583|DN90435_c0_g1:123-1100(+)	0.51093	-0.96881	3.2306e-06	5.4907	Molecular Function: serine-type endopeptidase activity (GO:0004252);; Biological Process: proteolysis (GO:0006508);;	Posttranslational modification, protein turnover, chaperones	PREDICTED: brachyurin-like [Musca domestica]
Gene|104495::DN6696_c1_g1::g.104495::m.104495|DN6696_c1_g1:54-710(+)	0.56401	-0.82622	0.00013106	3.8825	--	--	--
